# A global database of net primary production of terrestrial ecosystems

**DOI:** 10.1038/s41597-025-05773-4

**Published:** 2025-09-02

**Authors:** Marie Rodal, Sebastiaan Luyssaert, Manuela Balzarolo, Matteo Campioli

**Affiliations:** 1 PLECO, Department of Biology, Universiteit Antwerpen, Universiteitsplein 1, 2610 Antwerpen, Belgium; 2 Systems Ecology, A-LIFE, Faculty of Science, Van der Boechorstraat 3, 1081 BT Amsterdam, Netherlands; 3 Foundation — Euro-Mediterranean Center on Climate Change (CMCC), Via Marco Biagi 5, 73100 Lecce, Italy

**Keywords:** Ecosystem ecology, Ecological modelling

## Abstract

Net primary production (NPP) is a fundamental measure of biomass production in ecosystems. In terrestrial biomes, NPP lacks standard measuring protocols and is difficult to measure. Thus, despite decades of research efforts, NPP data are limited and heterogenous. Moreover, there continues to be a lack of global NPP databases containing harmonized estimates for all major ecosystem types and which account for both above- and belowground production. We present a global database containing records for both above- and belowground production for forests, grasslands, arid shrublands, northern peatlands and tundra at 456 sites. The records are reported as annual production (g m^−2^yr^−1^). The NPP data are complemented with detailed site and methodological information, including a method specific estimate for the measurement uncertainty, as well as ancillary data on climatic conditions, soil fertility and management status. This database provides a basis for comparative studies on local, regional and global scales, and may serve as an important benchmarking dataset for the development of DGVMs.

## Background & Summary

The net primary production (NPP) of an ecosystem is the remainder of the photosynthetic gain, or gross primary production (GPP), after accounting for the autotrophic respiration. The NPP represents the carbon available for other plant processes, in particular the production of structural biomass such as foliage, reproductive material, branch and stemwood, fine and coarse roots, and non-structural biomass such as volatile organic compounds, exudates and transfer to symbionts. The NPP is a key ecosystem service, primarily due to the provisioning of foodstuffs, fibres, fuel and construction material, but also owing to its pivotal role in the global carbon cycle and the resulting impact on the Earth’s climate.

Direct assessments of ecosystem NPP started in the 19^th^ century^1,2^ and intensified almost a century later with the International Biological Program (IBP) of the 1960s–70s^2^.This was followed by the publication of the first global synthesis of the net primary production of the biosphere in 1973^3^ and by the development of regression models linking NPP to easy-to-measure environmental variables like precipitation and temperature^4^. While the older studies were driven primarily by a concern about the Earth’s ability to sustain the growing human population, in the past few decades the focus has shifted in response to the threat posed by climate change towards the role terrestrial ecosystems play in the global carbon cycle^5^.

Since the early synthesis efforts began in the 1970s, the methods for estimating ecosystem NPP have improved significantly, and new biomes, ranging from tropical forests^6^ to arctic tundra^7^, have been investigated. However, despite decades of research accumulating in a large body of data, a consistent global NPP database is still lacking. This lack is thought to be in part due to methodological differences leading to poor harmonization of global data, but also due to a lack of synthesis effort in the decades following the IBP. The latter becomes evident when considering that the most recent global NPP database comprising both forested and non-forested biomes, i.e. the Osnabruck dataset^8^, was updated only until the 1980s. More recent databases comprise only one ecosystem type (e.g. refs. ^9–11^) and some only specific climatic zones (e.g. refs. ^12,13^). Additionally, databases (with the exception of e.g. refs. ^9,10^) rarely account for the measurement methodology and thus quality of the data, and often do not include estimates for belowground production. The latter is particularly problematic, as belowground production is estimated to account for over 30% of global annual NPP^14^, a percentage which is expected to be even higher in water limited ecosystems^15^.

Since the 1990s, the empirical estimates of NPP have been complemented by indirect production estimates stemming from the development of increasingly complex process-based vegetation models and remote sensing products derived from satellite imagery^2^. This has made it possible to study the temporal variability of NPP at a high resolution on both short and long timescales, something that remains impracticable based on field measurements. However, both process-based models and remote sensing products rely on empirically derived NPP relationships for construction, calibration and validation, which means that their reliability is ultimately constrained by the accuracy and precision of the underlying empirical data. Guidelines for benchmarking global vegetation models emphasize the need for consistent global NPP databases based on field measurements^16^, as currently modelers may have to rely too heavily on single site measurements or remote sensing products (e.g. MODIS NPP product^17^) to calibrate and evaluate their models. In view of the above, it becomes clear that there is a need for a consistent, global NPP database based on field measurements which (1) accounts for the production of the major aboveground (e.g. stemwood and foliage) and belowground (e.g. fine roots) organs, (2) accounts for methodological differences between NPP estimates, (3) includes estimates spanning all major biomes, specifically forests, grasslands, croplands, peatland, tundra and dry shrublands, and climate regions, and which (4) has sufficient geographical heterogeneity as to be globally representative.

Here, we describe such a database consisting of 456 sites that encompass the major terrestrial biomes. The database is structured by site, each given its own unique identifier and representing a known location and ecosystem type. For any site multiple NPP measurements may be available, either from multiple years or based on different methods. Each NPP entry includes the year in which the measurement was made, and is linked to a description of the methodology and the reference to the original publication. The large majority of the NPP estimates are obtained using biometric methods (ca. 95%), with the rest obtained indirectly from, e.g. flux measurements or site-specific, process-based models. The database can, for example, be used to study the environmental drivers of NPP, explore patterns in terrestrial NPP, in particular differences in above and belowground production, as well as serve as a benchmark for the calibration and validation of process-based vegetation models and remote-sensing products.

The database focuses on the NPP components that refer to the production of structural biomass, not the production of non-structural biomass (e.g. VOC, leachates from roots, transfer to symbionts), as estimates of the latter are extremely rare at the ecosystem level. However, as mentioned above, it is important to note that production of non-structural biomass is also part of the ecosystem NPP.

## Methods

The database comprises data on NPP gathered from the peer-reviewed literature, as well as ancillary data gathered from the peer-reviewed literature and global databases. The ancillary data consists of site data (e.g. geographical location, elevation, vegetation type, information on management and disturbances, information on soil texture and nutrient status, nitrogen deposition rates) and climatic data (e.g. mean monthly and annual temperature, total monthly and annual precipitation). In what follows, we first describe in detail how the NPP data was collected, what steps have been taken to ensure the consistency and quality of the data, and how a uniform data uncertainty is estimated based on the original measurements. Thereafter, we describe the ancillary data.

### NPP data

#### Data compilation

Central to the database are the NPP data which were collected through an extensive literature search. The literature search was conducted by entering selected combinations of keywords into the Web of Science including a term about the process (e.g. “net primary production”; “biomass production”), a term about the biomes (e.g. forest, grassland, peatland) and, when appropriate, keywords indicating the climate (e.g. temperate, boreal) or the ecosystem components (e.g. fine roots). A first extensive search was performed in 2013–2014. In 2022–2023, a second search was done with the aim to better balance the climate distribution as well as the representation of the biomes, paying particular attention to unrepresented climate zones (e.g. very arid or very cold regions) and biomes (e.g. tundra and dry shrublands).

**Fig. 1:**
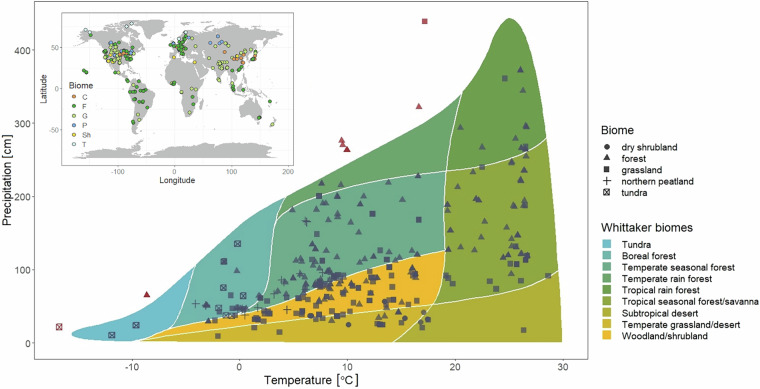
Whittaker plot of the sites in the database, with outliers marked in red.

The search resulted in peer-reviewed NPP databases, of which individual datapoints were added to our database, and individual site studies. The NPP data was then extracted from the text, tables or figures (using e.g. PlotDigitizer) contained therein. The first search period (2013–2014) resulted in 398 sites, while the second (2022–2023) yielded another 58 sites. In Fig. 1, a Whittaker plot^18^ illustrates the distribution of the selected sites according to long-term mean annual temperature and total precipitation, extracted from the WorldClim V.2 database^19^.

#### Selection criteria and quality control

The NPP data included in the database was selected based on stringent methodological criteria:

1. The NPP estimate must be for a specific site with a known geographical location. This criterion excludes regional averages.
2. The data must be from direct assessments. These were primarily from biometric methods (94%), though estimates derived from site-specific flux assessments or estimates from process-based models constructed or calibrated with site-specific data, were also accepted. Conversely, remote-sensing data as well as model studies which were not calibrated for and evaluated at a specific site were excluded.
3. The measurement methodology must be described in sufficient detail to estimate the uncertainty, using the method-specific uncertainty estimate described below.
4. The methods to measure above- and belowground production (ANPP and BNPP, respectively) must meet stringent criteria (Table 1). The inclusion or exclusion of any given method is based on previous analyses (see refs. ^14,20–22^). The advantages and disadvantages of the selected methods are listed in Table 1; any method not listed has been excluded from consideration.

**Table 1:** Measurement methods for NPP by component and the associated uncertainty reduction factor (RF).

Component	Method	Advantages and Disadvantages	RF
Aboveground production	Isotope turnover	Most accurate but also complex^35^.	0.1
	Sum of increments in live biomass adjusted for decomposition	Time-consuming but accurate. Accounts for mortality and decomposition between measurements; however, for certain species it can be difficult to distinguish live and dead biomass^21^.	0.2
	Sum of increments in live biomass	Time-consuming but relatively accurate. Assumes that no plant mortality occurs between measurements^21^.	0.3
	Peak standing crop	Relatively easy to apply. Assumes that all standing biomass is new growth, i.e. that all growth occurs in the measurement year and that there is no plant mortality. Only suitable for annual plants^21^.	0.4
	Maximum minus minimum live biomass	Relatively less time consuming (in comparison to similar approaches). Does not account for decomposition and assumes that there is no plant mortality between measurements^21^.	0.4
	Allometric/biometric methods	A non-invasive and non-labor-intensive method, but indirect and potentially inaccurate when site- and species specific allometric relations are not available^22^.	0.4
	Fixed proportion of other NPP component	Very easy to apply but inaccurate, only appropriate when applied to minor components (see Table 2)^36^.	0.8
Belowground production	Isotope turnover	Most accurate but also complex^20^.	0.2
	Minirhizotron or root window (fine roots)	Accurate. Allows for continuous observation of root growth but is labor intensive and indirect (root length or surface needs to be translated into biomass). May disturb root-growth^14,20^	0.3
	Ingrowth core (fine roots)	Relatively little time consuming and accurate. Assumes no mortality during measurement. Therefore, accuracy depends on length of measurement interval^14,20^	0.3
	Allometric relations (coarse roots)	Only appropriate for coarse roots. A non-invasive and non-labor-intensive method, but indirect and potentially inaccurate when site- and species specific allometric relations are not available^14^.	0.6
	Carbon budget and mass balance (all roots)	Indirect method. Its accuracy depends on the degree the assumptions (e.g. steady state) are met and the quality of the measurements from which the total root NPP is indirectly derived^14,20^	0.6
	Sequential coring, with sum of increments in live and dead biomass (fine roots)	Time consuming. Accounts for mortality and decomposition, but distinguishing live from dead roots is potentially difficult, leading to erroneous estimates^14,20^	0.7
	Sequential biomass coring (fine roots)	Time consuming but relatively simple. Does not account for mortality and decomposition^14,20^	0.8
	Fixed proportion of other NPP component (coarse roots)	Only appropriate for coarse roots, that can be estimated as a fraction of aboveground wood production. Very easy to apply but potentially inaccurate when site- and species specific allometric relations are not available^14^.	0.9
Total net primary production	Radiocarbon	Indirect method. Assumes that the soil is in a steady state, and therefore not suitable in areas with active soil erosion^37,38^	0.6
	Peat formation in peatlands	Accuracy depends on the species present at site. E.g. *Sphagnum* mosses form a litter more recalcitrant than vascular plants^39^.	0.6
	Process-based model	Allows for the study of biomass production dynamics at long- and short-time scales, but requires extensive parametrization based on independent experimental data for the specific site^40^.	0.6
	Flux components, incl. eddy covariance measurements	Indirect method. Its accuracy depends on the degree the assumptions (e.g. steady state) are met and the quality of the measurements from which the total NPP is indirectly derived^41^.	1
5. Estimates for the main components of ANPP (e.g. stemwood, foliage) and BNPP (e.g. fine roots) must be available for the site (for a definition of the main components of above- and belowground production, see Table 2). Thus, sites where either of these components were missing were discarded. Coarse root production is typically estimated using allometric relationships^22^. However, coarse wood NPP can also be calculated as a percentage of some other wood NPP component (e.g. aboveground wood; see ref. ^23^). As this method is also widely applied, coarse root production estimates have been included also when derived from other wood NPP component in the original study. For non-forested biomes, we have made the assumption that all roots are accounted for in the fine root estimate.

**Table 2:** Main and minor components of aboveground NPP by biome and climate region.

Ecosystem type	Main NPP components	Minor NPP components
Boreal forests	Stem, foliage, understory, fine roots	Reproductive material, herbivory, coarse roots^(a)^
Tropical forests	Stem, foliage, fine roots	Understory, reproductive material, herbivory, decomposed leaves, coarse roots^(a)^
Forests (excl. tropical and boreal forests, and plantations managed for fruit production)	Stem, foliage, fine roots	Understory, reproductive material, herbivory, coarse roots^(a)^
Plantations (forests) managed for fruit production (e.g. apple orchards and palm oil plantations)	Stem, foliage, reproductive material, fine roots	Understory, herbivory, coarse roots^(a)^
Grassland	Herbaceous component, fine roots	Overstory (trees), herbivory
Cropland (herbaceous)	Herbaceous component, fine roots	Overstory (trees), herbivory
Northern peatland/tundra *without* substantial non-vascular vegetation	Herbaceous component, fine roots, stem increment and foliage (for shrubs)	Overstory (trees), non-vascular component, reproductive material, herbivory, coarse roots
Northern peatland/tundra *with* substantial non-vascular vegetation	Herbaceous component, non-vascular component, fine roots, stem increment and foliage (for shrubs)	Overstory (trees), reproductive material, herbivory, coarse roots
Woodland savanna/forested dry shrubland	Herbaceous component, stem increment, foliage, fine roots	Herbivory, coarse roots

^(a)^For woody biomes, coarse roots are classified as a minor component of NPP, even when significant, as lack of a coarse root production estimates was not deemed sufficient for exclusion from database. This is because coarse root production is very rarely measured directly, but usually estimated indirectly using allometric relations or as a percentage of the aboveground components. Thus, provided the aboveground components are properly accounted for, a coarse root estimate can be obtained from these components (e.g. ref. ^23^).
6. The NPP data must be integrated over a year, comprising the growing season. We did, however, not require that the annual estimates for the different components of NPP (i.e. ANPP and BNPP) be given for *the same* year. A mismatch between the measurement year for the main above and belowground components only occurs for 5% of the sites.

While most of the NPP estimates in the database are derived from field assessments, we did not categorically exclude data derived from control plots or manipulation experiments. In total, they comprise 7% of the sites, and are marked as such in the database. In some cases, when appropriate (e.g. fertilization experiments in grasslands), NPP data of treatment plots were also retained (5% of sites; see section “Management”).

#### Consistency of the NPP data

**Fig. 2:**
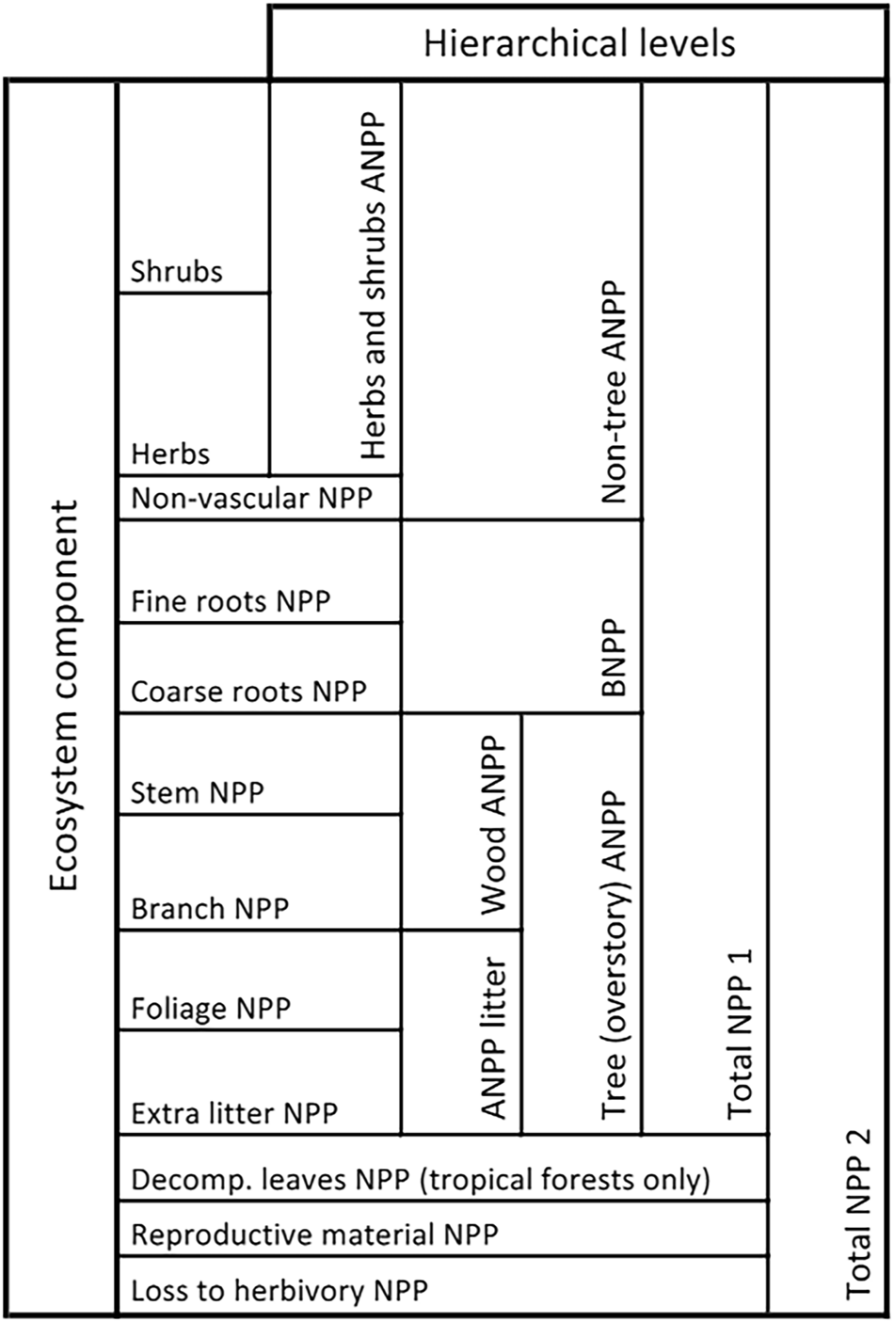
Hierarchical framework for net primary production.

In spite of the aforementioned selection criteria, the different ways of reporting NPP estimates (e.g. each component separately versus sum total) and the missing minor components resulted in inconsistencies. To account for this issue and make it easier for the user of the database to identify missing minor components, eight intermediary summation levels of ANPP, BNPP and total NPP (sum of ANPP and BNPP) were calculated (Fig. 2) to indicate whenever a given component estimate can be considered complete^9^.

1. *Herb and shrub ANPP* includes the sum of herbs and shrubs aboveground NPP and is only reported when NPP of both herbs and shrubs has been appropriately accounted for, or is identified as not present at site.
2. *Non-tree ANPP* is the sum of the ANPP of herbs, shrubs and non-vascular vegetation, i.e. all the components not considered to be trees.
3. *Wood ANPP* is the sum of branch and stem increments (only relevant for biomes with trees).
4. *Total litterfall ANPP* is the sum of leaf litter biomass and extra litter biomass (e.g. small branches or reproductive material whenever these are reported together), which are generally assumed to be equal to the annual NPP of these compartments.
5. *Tree ANPP*, or overstory vegetation ANPP, is the sum of *wood ANPP* and *total litterfall ANPP*.
6. *BNPP* is the production of coarse and fine roots.
7. *Total NPP 1* is the sum of *tree ANPP* (overstory), *non-tree ANPP* and *BNPP*.
8. *Total NPP 2* is the sum of *Total NPP 1* and NPP lost to herbivory, reproductive material (when not considered in *total litterfall ANPP*) and, for tropical forests, the unaccounted leaf litter due to the rapid decomposition of leaves^22^.

For sites where estimates for different components of NPP are given as separate entries in the NPP estimates table contained in the database, as would be the case whenever the measurements were done in different years or using different methods, no summation of these components is made. However, the sites can be considered complete, depending on the application, and are therefore included. When available (for 3 sites), estimates for VOC, leaching from fine roots and transfer to symbionts were included in above- and belowground production, respectively.

#### Uncertainty estimates for the NPP data

To have a consistent estimate for the uncertainty of the reported data, we have opted to follow refs. ^9,24^. and establish a method-dependent uncertainty for each site, as opposed to reporting the uncertainty (e.g. standard deviation of measured values) as given in the source material. The reason for this is that it is not possible to integrate the uncertainty values calculated in single studies because the applied methodologies differ and different methodologies come with different biases both in direction and magnitude^20–22^. The uncertainty estimation accounts for biome-specific and method-specific uncertainties, weighted by the monitoring time span. Thus, to account for the variability in accuracy between different NPP estimates, we introduce the site-specific uncertainty S_ij_^k^:

1 $${S}_{{ij}}^{k}=\frac{\left({P}_{i}^{k}\times R{F}_{j}^{k}\right)}{{\left({l}_{{ij}}^{k}\right)}^{\frac{1}{2}}}$$

where ${P}_{i}^{k}$ is the biome specific variance of the NPP component $k$ for the biome $i$, $R{F}_{j}^{k}$ is the method specific reduction factor for method $j$ and ${l}_{{ij}}^{k}$ is the length of the time series included to account for the reduction in uncertainty stemming from taking a multiyear average. Each NPP component and biome type has its own ${P}_{i}^{k}$ (see Supplementary Information), which is derived from the data in the database based on the following formula:

2 $${P}_{i}^{k}=\frac{{D}_{9i}^{k}-{D}_{1i}^{k}}{2}$$

where ${D}_{9i}^{k}$ and ${D}_{1i}^{k}$ refer to the 90^th^ and 10^th^ percentiles for biome $i$ and component $k$, respectively. The method specific reduction factor $R{F}_{j}^{k}$ is in the range of 0.1 to 1.0, and accounts for the difference in method accuracy for the NPP estimate. In practice, the more accurate the method, the more the uncertainty is reduced, thus the smaller $R{F}_{j}^{k}$ is. We provide $R{F}_{j}^{k}$ for seven aboveground methods, eight belowground methods and four total NPP methods, where the latter refers to methods (e.g. Eddy covariance) where above- and belowground production cannot be distinguished (see Table 1).Broadly the measurement methods are “ranked” based on (1) directness of approach (e.g. direct measurement of new biomass or indirect measurements of proxies such as plant height) and assumptions (e.g. steady conditions), (2) number of seasonal measurements (e.g. max-min methods in perennial grasslands are less accurate than sum of multiple increments) and (3) the extent to which mortality and decomposition is counted for (e.g. not at whole, partly or in full). Our classification was based on previous relevant comparative methodological analyses (e.g. ^14,20–22^). As seen in Table 1, the $R{F}_{j}^{k}$ of aboveground methods are generally lower than the $R{F}_{j}^{k}$ of belowground methods, resulting in a stronger error reduction for above ground NPP estimates. This is because belowground methods are in general considered to be less precise, as it is not generally possible to get a pre-measurement overview of the spatial distribution of belowground biomass, which leads to errors when the estimate is scaled for the whole ecosystem.

To compute the uncertainty for the total NPP estimate for any given site one should ideally estimate the uncertainty for each component separately and then use error propagation to obtain the total uncertainty. However, this would require a more careful division of the method-specific error reduction factor than we have chosen to do here. Thus, for each measurement, we report a separate uncertainty reduction factor for the main above- and belowground parts only, and not for every component contained in these estimates. If multiple methods, corresponding to different uncertainty factors, have been employed to measure the main (either above- or belowground) components, the mean rounded to one decimal place is reported. As an example, consider a site where fine roots are measured using minirhizotrons (${RF}$ = 0.3) and the coarse roots are measured using allometric relations (${RF}$ = 0.6) (Table 1). For this site the ${RF}$ for the total BNPP estimate is given as 0.4, which is the mean averaged to one decimal point in favour of the fine roots estimate, since the fine roots with their high turnover rate are in general responsible for the majority of BNPP, and thus the largest source of uncertainty for the BNPP estimate. Thus, for each measurement entry in the NPP estimates table, one can compute the uncertainty of the above- and belowground estimates (${S}_{{in}}^{{ANPP}}$ and ${S}_{{im}}^{{BNPP}}$, respectively), and then use error propagation to compute the error for the total NPP (TNPP) estimate, ${S}_{{inm}}^{{TNPP}}$:

3 $${S}_{{inm}}^{{TNPP}}=\sqrt{{{(S}_{{in}}^{{ANPP}})}^{2}+{{(S}_{{im}}^{{BNPP}})}^{2}}$$

where *n* and *m* refer to the methods used for measuring above- and belowground production, respectively. Given that multiple measurements may be available for each site *i*, the total uncertainty for site ${i}$ and component ${k}$ is

4 $${S}_{i}^{k}=\frac{1}{{N}_{i}^{k}}\sqrt{\mathop{\sum }\limits_{j=1}^{{N}_{i}^{k}}{\left({S}_{{ij}}^{k}\right)}^{2}}$$

where ${N}_{i}^{k}$ denotes the total number of measurements available for component $k$ at site $i$. The resulting uncertainty values can e.g. be used as weights when constructing regression models to account for the varying quality of the data. To illustrate the impact of including the uncertainty values as weights in regression analysis, we include an example (see Supplementary Information for the details) using the Miami Model^4^. When fitting a non-linear relationship between NPP, temperature and precipitation (see Supplementary Information), accounting for uncertainty could result in a 40% lower NPP estimate than fitting the exact same relationship while ignoring measurement uncertainties.

### Ancillary data

#### Data compilation

For each site included in the database, a follow-up literature search was performed to obtain as much additional information about the site as possible e.g. elevation, vegetation type, information on management and disturbances, information on soil texture, pH and nutrient status. Ancillary data could come from three sources: (1) data extracted from the original study, (2) if missing from the original study, the corresponding author was contacted who occasionally provided ancillary data, and (3) data extracted from global databases e.g. nitrogen deposition rate and climate reconstructions including, but not limited to, temperature, precipitation, potential-evapotranspiration and vapor-pressure for the measurement years as well as a data describing the long-term climate (1970–2000 aggregate).

#### Biome and management classification

The sites were classified into six biomes: forest, grassland, cropland, tundra, (northern) peatland and dry shrubland (see Table 3).

1. *Forest* encompasses all forested biomes including plantations intensively managed for wood, fruit, or rubber production. The sites are further divided into managed and unmanaged. Planted forests with limited anthropogenic impact after planting are also considered as natural, while managed forests are forests which have been thinned or harvested in the last 50 years, fertilized in the last 25 years, or are newly established plantations.
2. *Grassland* includes prairies, non-forested savannas, herbaceous dominated wetlands and marshes, steppes and meadows. In case of transition zones (woody grasslands), we defined the biomes (forest or grassland) based on whether the component with the largest productivity was trees (forest) or herbs (grasslands). Unmanaged grasslands include grasslands experiencing yearly burns and grasslands which are lightly to moderately grazed or mowed. Managed grasslands are primarily fertilized and irrigated grasslands, but also include grasslands which were established in the same year as the measurements were made.
3. *Cropland* refers to agricultural land not dominated by woody vegetation and are all managed.
4. *Tundra* includes arctic and alpine tundra and is considered to be unmanaged unless fertilized as part of manipulation experiments.
5. *Peatland* only refers to northern peatland, including boreal mires and heathland (tropical peatlands are typically dominated by woody vegetation and therefore included in forests). Peatlands are considered to be unmanaged unless fertilized as part of manipulation experiments.
6. *Dry shrubland* refers primarily to arid or semi-desert ecosystems, but also some dry savannas are included in this category provided the vegetation cover is reduced and patchy and are considered to be unmanaged unless fertilized as part of manipulation experiments.

**Table 3:** Biome and management classification.

Biome classes^(a)^	Biome definition	Management status	
		Natural	Managed
*Forests (forests, woodlands and plantations)*	Ecosystem dominated (>50% production) by woody vegetation not in arid climate.	*Old-growth with minimal disturbance *Natural succession due to fire/windthrow and at least 10 years after disturbance *Unmanaged or with low human impact (e.g. understory grazing) in the last 50 years *Planted forests without any intervention after planting and at least 10 years old	*Thinning/harvest in the last 50 years *Newly (<10 years) established plantation *Fertilization in the last 25 years *Managed for fruit/rubber production
*Grasslands*	Ecosystem dominated ( > 50% production) by herbs but below the treeline	*Pristine *Natural succession due to fire and at least 10 years after disturbance *Minor human impact in the past and protected for at least 15 years *Low-moderate grazing and/or annual burning *Established/restored grassland (>10 years before measurements) *Mowing (non-intensive)	*Established same year of measurements on agricultural land *Fertilization and/or irrigation
*Peatlands*	Ecosystems co-dominated by shrubs and herbs (each accounting < 50% production) in cold climate (boreal, hemiboreal).	*Pristine *Minimal disturbance (grazing)	*Fertilized as part of fertilization experiments^*(b)*^
*Tundra*	Any terrestrial ecosystem above the treeline	Pristine	Fertilized as part of fertilization experiments^*(b)*^
*Croplands*	Grain or tuber bearing annual plants	*(only managed)*	*Fertilization *Established same year of measurements on agricultural land *Established same year of measurements on grasslands
*Dry shrubland*	Dry ecosystems co-dominated by herbs and shrubs with a low degree of vegetation cover	Pristine	Fertilized as part of fertilization experiments^*(b)*^

(a) Biome class as provided in the source papers (b) We have included some sites which have been fertilized or irrigated as part of manipulative experiments, and these are, because of the coarse management categorization, classified as managed sites.

The biome and management classification is summarized in Table 3.Urban green spaces were not considered. Seldom represented biomes (e.g. temperate heathlands, few sites) were classified within the major six biome types based on biome similarities for climate and vegetation properties (e.g. a temperate heathland was classified as grassland when herbaceous vegetation was as important as shrubs). The management classification “managed” and “unmanaged” for forests and grasslands is a very rough distinction. However, ref. ^24^ showed that within a three-level division (managed, semi-natural and natural) the production dynamics of semi-natural ecosystems were similar to the ones of natural ecosystems and that a two-level category system can capture the main effect of management.

#### Soil data and site fertility estimate

In addition to site observations of soil characteristics and site fertility, information on soil texture and nutrient status was extracted from the Harmonized World Soil Database (HWSD) version 2.0^25^. The HWSD dataset has a resolution of 30 by 30 arc seconds (approximately 1 km^2^), and contains 20 numerical variables, including but not limited to pH, C:N ratio, soil texture, available water capacity, nitrogen and organic carbon content, bulk density and electric conductivity. For any given site only the characteristics of the topsoil layer (0–20 cm) were extracted.

Nitrogen deposition estimates were extracted from a model-derived dataset presented in ref. ^26^. The dataset contains global estimates of dry and wet nitrogen deposition at a spatial resolution of 2.0° by 2.5°, and was obtained from the GEOS-Chem Chemical transport model. The dataset contains estimates for selected years in the period 1984 to 2016, and thus we extracted for each site the total nitrogen deposition (the sum of wet and dry deposition) for the year closest to the NPP measurement year.

Based on the soil characteristics, the nitrogen deposition data, and the information on soil fertility and nutrient status collected from the literature, a subset of the sites (78%) was divided into low (L), medium (M) and high (H) fertility sites. The classification is based on all information available from the site, in particular physical and chemical properties (e.g. soil type, soil texture, carbon and nitrogen content, pH), but also species composition at the site, presence of permafrost, vegetation growth relative to other similar sites, statements from the PIs or others with expert knowledge of the site, and any additional information which can be used to discern the fertility status of the site. The information used to discern the soil fertility is provided in its own table in the database. Some of this data were already presented in ref. ^24^.

#### Climate data and koppen-geiger climate classification

The climatic variables were divided into three groups: (i) climatic variables for the years in which NPP measurements were reported; hereafter referred to as *short-term climatic variables*, (ii) 1970–2000 aggregate climatic variables, hereafter referred to as *long-term climatic variables* and (iii) bioclimatic variables.

*Short-term climatic variables* were extracted from the CRU TS (Climatic Research Unit gridded Time Series) dataset^27^. The dataset has a resolution of 0.5° by 0.5°, and contains monthly estimates from 1951 to 2022. The extracted variables include (1) primary variables such as mean temperature and precipitation, (2) secondary variables such as number of wet days and vapor pressure, and (3) derived variables such as number of frost days and potential evapo-transpiration (PET). Additionally, the aridity index SPEI^28^ derived from the precipitation and potential-evapotranspiration data contained in the CRU-TS dataset is included.

*Long-term climatic variables* (1970–2000 aggregate) were extracted from the WorldClim2 database^19^ with a resolution of 30 by 30 arc seconds (approximately 1 km^2^). Variables include long-term monthly values of temperature (mean, min and max), radiation, wind velocity, vapor pressure and precipitation. From this data, we determined the climate region for any given site based on the Koppen-Geiger climate classification as defined in ref. ^29^. The WorldClim2 database does not report PET, thus we also extracted long-term monthly PET from the Global Aridity Index and Potential Evapo-Transpiration Database (Global AI PET V.3)^30^, where PET is estimated from the WorldClim2 time series data using the same procedure, namely the FAO-56 Penman-Monteith method^31^, as for the CRU dataset. Additionally, the monthly values for the aridity index AI were extracted from the Global AI PET database, where AI was defined as

5 $${AI}\,=\,\frac{{PRE}}{{PET}}$$

where ${PRE}$ denotes precipitation and ${PET}$ is the potential evapo-transpiration (note that the index is only defined whenever ${PET}$ is not zero). ${AI}$ gives an indication of the degree to which there is a water deficit, as a value of much less than 1 indicates that the amount of potential evapo-transpiration is not matched by an equal amount of precipitation.

*The bioclimatic variables* are derived from the long-term climate data contained in the WorldClim datasets^19^. The bioclimatic variables are variables which are expected to be of particular ecological importance, and include, among others, mean temperature of the coldest quarter, total precipitation in the warmest quarter, temperature seasonality and mean diurnal range. In addition to the 19 variables reported by ref. ^19^, yearly averaged PET and ${AI}$ from ref. ^30^ were also included. Both datasets have a resolution of 30 by 30 arc seconds (approximately 1 km^2^). Following ref. ^30^, we can, based on the yearly values of ${AI}$ for each site, divide the sites into humid (${AI}$ > 0.65), dry sub-humid (0.5 < ${AI}$ < 0.65), semi-arid (0.2 < ${AI}$ <0.5), arid (0.03 < ${AI}$ < 0.2) and hyper arid (<0.03) sites.

#### Other ancillary data

Also included is additional site information such as geographical location, elevation, species composition, functional type of dominant species and type of study, where the latter indicates whether the data collected for the given site comes from a field assessment or a manipulation experiment and can be used to e.g. exclude data collected from control experiments whenever necessary.

## Data Records

The database is structured by site, with each site given a unique identifier, and consists of a total of 12 distinct tables, of which six exclusively contain ancillary data such as soil characteristics and climate data. Of the remaining six tables, one table contains the site information data (e.g. geographical coordinates, altitude, vegetation characteristics such as species composition and functional type of dominant species, biome, management status, climate classification), four contain information on the methodology (two for above- and two for belowground), and one contains the NPP data. The database together with a reference list for all the published and unpublished data included in the database are available at the Figshare repository^32^.

### Distribution according to biome type, climate region and management status

The current version of the database contains 456 distinct sites; 206 forest sites, 145 grasslands, 34 croplands, 34 peatlands, 21 tundra sites and 16 dry shrublands. The sites, when divided into climate regions according to the Koppen-Geiger climate classification, distribute as follows: 191 cold sites, 125 temperate sites, 80 tropical sites, 45 arid sites and 14 polar sites.

Of the 456 sites in the database, 303 are considered to be unmanaged, while 152 are managed, of which 34 are croplands.

### NPP data records and uncertainty estimate

**Fig. 3:**
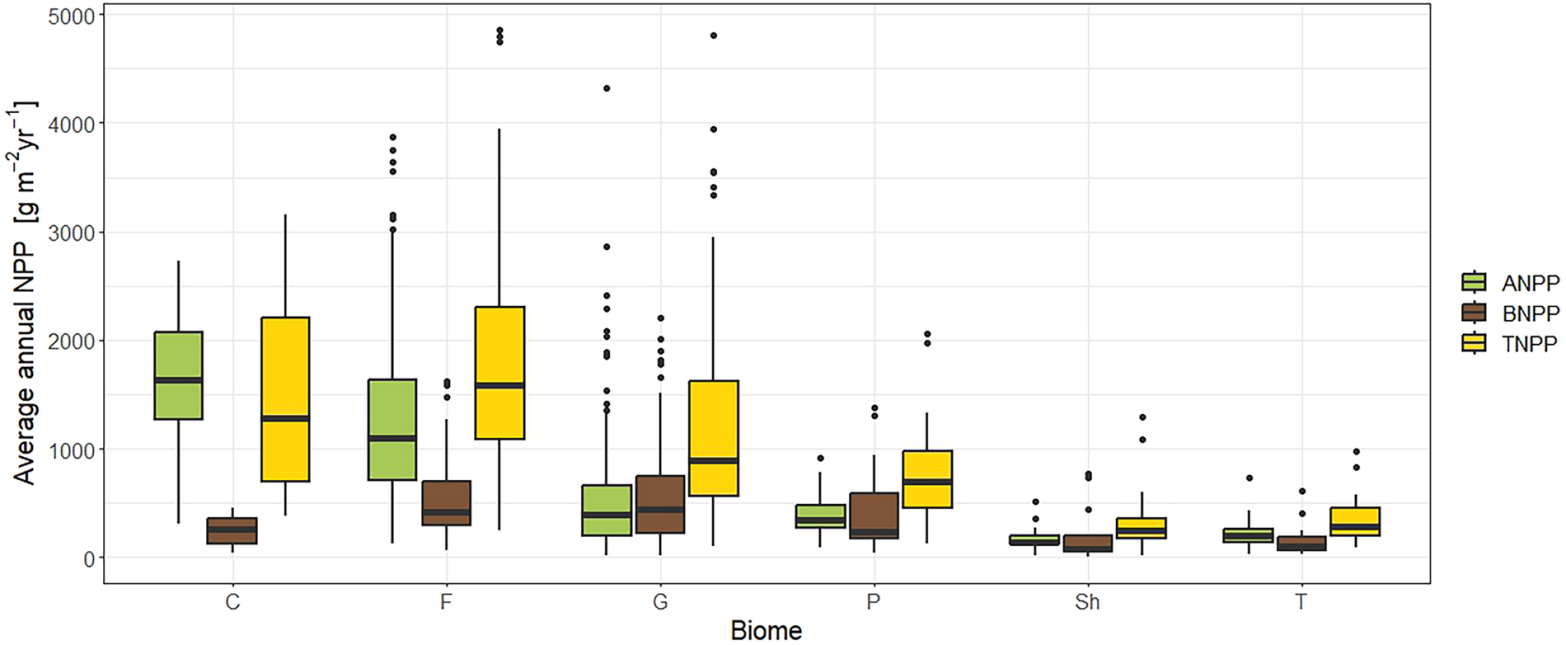
Boxplots of NPP values by biome, where the biomes are croplands (C), forests (F), grassland (G), northern peatland (P), dry shrubland (Sh) and tundra (T).

**Fig. 4:**
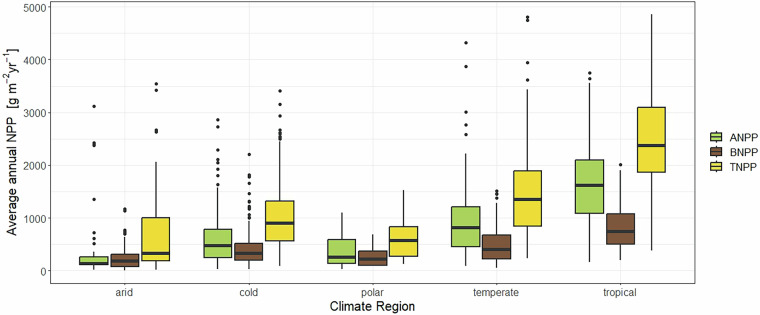
Boxplots of NPP values by climate region.

For each site at least one, but possibly more NPP observations are recorded. The *mean annual* NPP data is given in grams of dry mass per square meter per year (g m^−2^ yr^−1^); if converted from grams of carbon (C g m^−2^ yr^−1^), then the conversion factor is given in the column *carbon content*. In this manuscript, to aggregate the data by site, we took the mean of each column containing NPP estimates for a given site, and summed over all components to get total NPP. The uncertainty of the NPP estimate was derived through error propagation by first computing the uncertainty of the above- and belowground components using the method described previously, and then using error propagation to compute the total uncertainty of the sum of these components. Boxplots showing the distribution of the mean annual NPP according to biome type and climate region data is shown in Figs. 3, 4, respectively.

### Spatial and temporal coverage

**Fig. 5:**
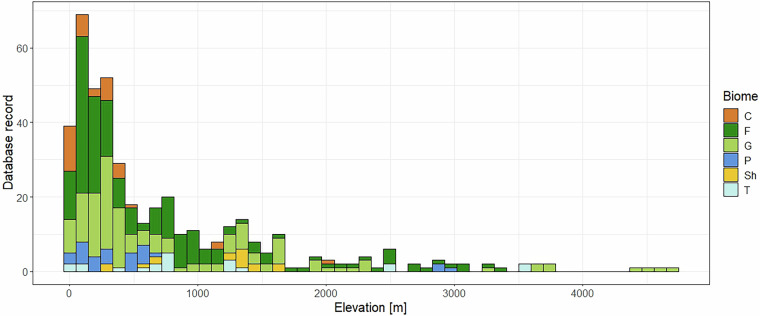
Site elevation distribution plot.

In terms of its geographical extent, the database contains sites from every continent distributed over 50 countries (Fig. 1). North America (including Mexico) accounts for over one-third of the sites (175 sites), while Asia (including Russia past 50 degrees longitude) accounts for another quarter of the sites (112 sites). The remaining sites are distributed in Europe (74 sites), South America (54 sites), Africa (32 sites), Oceania (8 sites) and the Middle East (1 site). The majority of the sites are found at an elevation below 1,000 meters altitude. However, high altitude sites are also represented, with 70 sites falling in the 1,000 to 2,000 meter range, and 39 sites falling in the 2,000 to 5,000 meter range (Fig. 5).

**Fig. 6:**
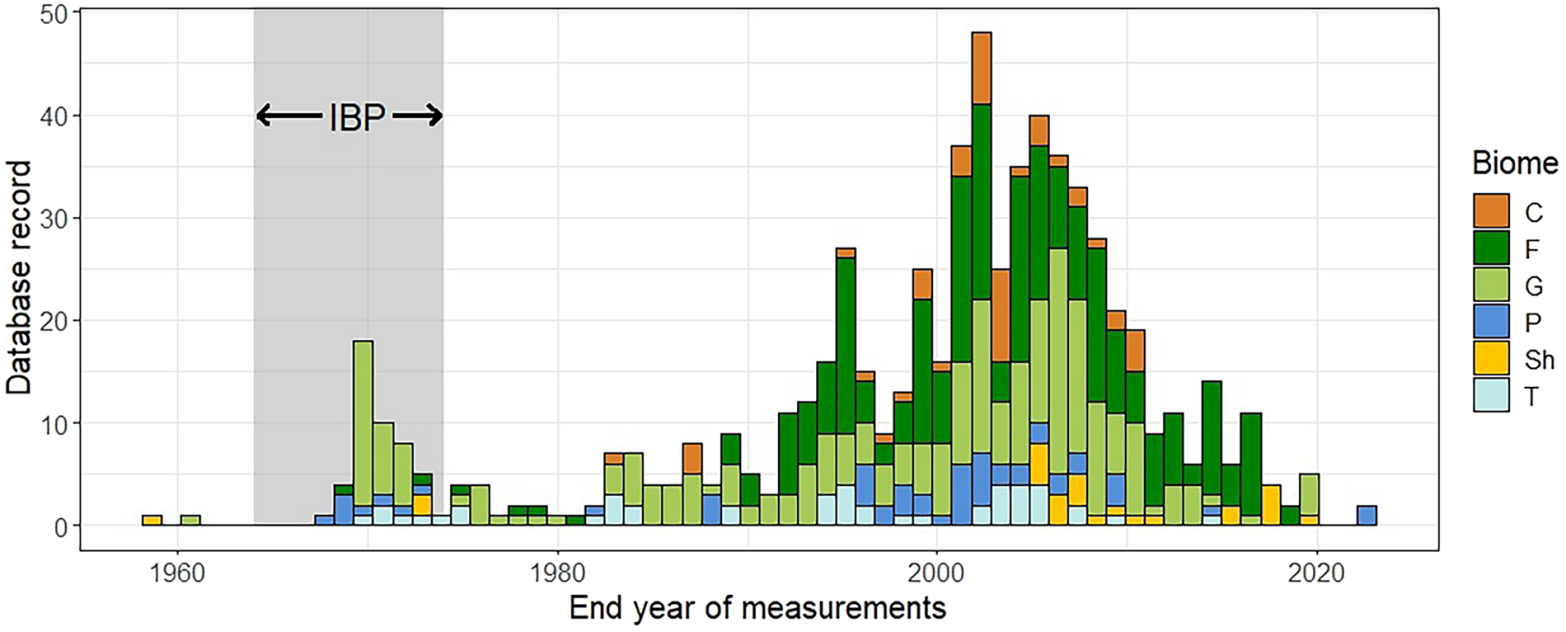
Temporal coverage plot with the years of the International Biological Programme (IBP) shaded in grey.

The database records cover the period from 1959 to 2023, with more than 70% of recorded measurements falling between 1995 and 2015 (Fig. 6). In this manuscript, measurement year was defined as either the last year of the measurements reported in the source material, or as the year of publication in the few cases where the measurement period was not known. At 42% of the sites, NPP was measured for a single year, while 50% of the sites have measurement periods between 2 and 5 years, leaving less than 8% having a measurement period of between 6 and 16 years. Note that the measurement period gives the interval between the first and the last measurements, but does not necessarily indicate that all components of NPP were measured consistently during the whole measurement period. Most sites (406) have separated estimates for ANPP and BNPP. The ANPP estimates have a coverage of 1,122 site-years, and the BNPP estimates have a coverage of 1,103 site-years, yielding an average of approximately 3 years per site. For sites (50) for which only total NPP is available, the coverage is 99 site-years, with an average of approximately 2 years per site. Hence, an approximate estimate for the total number of site-years for the database is 1,200.

### Database sources and methods

The majority (67%) of the NPP records are from single studies published in the peer reviewed literature, while the remainder are from global NPP databases^9^ (20%), smaller datasets gathered for meta-analysis^10,24,33^ (12%) and other sources including PhD thesis’ and books (<2%).

## Technical Validation

After compiling the database, the following checks were applied to the NPP records:

1. The geographical locations of the sites were plotted to check whether the sites were on land and whether the location corresponded with the qualitative description in the source material. For six sites, discrepancies were double checked and site coordinates amended whenever necessary.
2. Summation of the NPP components in the database were checked against the reported values for consistency. Inconsistencies were found for six sites and the entry was double checked.
3. Boxplots by biome or climate region were used to identify site-level outliers for average annual ANPP and BNPP. The 68 sites identified as outliers were then double checked: for 57 sites the value was confirmed, while for 11 sites the typo was corrected.

## Usage Notes

The database is available both as an Excel file and as a SQLite file. Extensive documentation on the structure of the database (including description of tables and column headers) is found in the supplementary material accompanying this manuscript and in the database repository.

Despite making attempts to balance the database with respect to biome type and geographical location, the database is still heavily skewed towards forests and grassland biomes and towards Eurasia and North America. The user should be aware of this bias when attempting to draw conclusions from the database.

The database is intended to be globally representative in terms of biome distribution, climatic regions and geographical extent, as opposed to attempting to collect *all* of the sites pertaining to a given biome type and climate region. Therefore, it is conceivable that some otherwise valid data has not been included in the database. This is particularly likely for the most frequently studied biome types (e.g. temperate forests and grasslands), as more effort was invested in finding valid sites and additional measurements for underrepresented biomes and climate regions.

Data from treatment experiments have in certain cases (36) been included. The treatments concerned are predominantly fertilization, irrigation, grazing and controlled burning of grasslands situated in North America. Corresponding control plots of the treatments were available for 22 experiments; these were also included. If the user desires to exclude these sites, this can be done simply based on the column “type study” in the table “site information”. For more information the user is referred to the documentation file accompanying the database and this manuscript.

Finally, the database has not been gapfilled to account for missing minor components of NPP (e.g. herbivory, understory in non-boreal forests and reproductive material). Users interested in doing so are referred to refs. ^24,34^. for an explanation on how this can be done and how one can account for the resulting increase in uncertainty. Missing minor components can be identified by referring to the methodology tables contained in the database.

## Supplementary information

Supplementary material / Appendix

Plotting routine for manuscript figures

Documentation file NPP DB

## Data Availability

The R code describing how the environmental data was extracted, how the Koppen-Geiger climate classification was determined, as well as the R code for the figures found in this manuscript are available at the Figshare repository^32^.
