# Supplementary material for: A global database of net primary production of terrestrial ecosystems
Source: Sci Data. 2025 Sep 2;12:1534. doi: 10.1038/s41597-025-05773-4 (PMC12405561; doi:10.1038/s41597-025-05773-4)
Supplement: Supplementary file 1 — Supplementary material / Appendix [file 41597_2025_5773_MOESM1_ESM.pdf]

Supplementary Material

A. Accounting for measurement uncertainty in the Miami Model

The Miami model was developed in ref. [1], and involves fitting the following two non-linear regression models:

$$NPP_P = 3000\,(1 - e^{-c\,P}) \qquad (A.1)$$

$$NPP_T = \frac{3000}{1 - e^{a - b\,T}} \qquad (A.2)$$

where T and P denote the mean annual temperature and total annual precipitation, respectively, and a, b and c are fitting parameters (see Table A.1). The pre-factor of 3000 denotes the maximum achievable mean annual NPP value and was inferred by ref. [1] from experimental data. The model can be used to estimate NPP from temperature and precipitation data by taking

$$NPP = \min(\,NPP_T\,, NPP_P\,) \qquad (A.3)$$

The fit of NPP_T is shown in Figure A.2, and the fit of NPP_P is shown in Figure A.1, with the red line denoting the un-weighted fit and the blue line denoting the weighted fit. For the fit only NPP data from unmanaged sites was used implying that croplands are entirely excluded. The uncertainty estimate was calculated as described in the main text, using the P_i^k values for ANPP and BNPP as listed in Table A.1, and Eqns. (1), (3) and (4) (main manuscript).

Figure A.1: The Miami Model. A non-linear fit of NPP vs. precipitation fitted employing a weighted (blue) and un-weighted (red) non-linear regression. The fitting parameter, c, for the two fits are given in Table A.1.

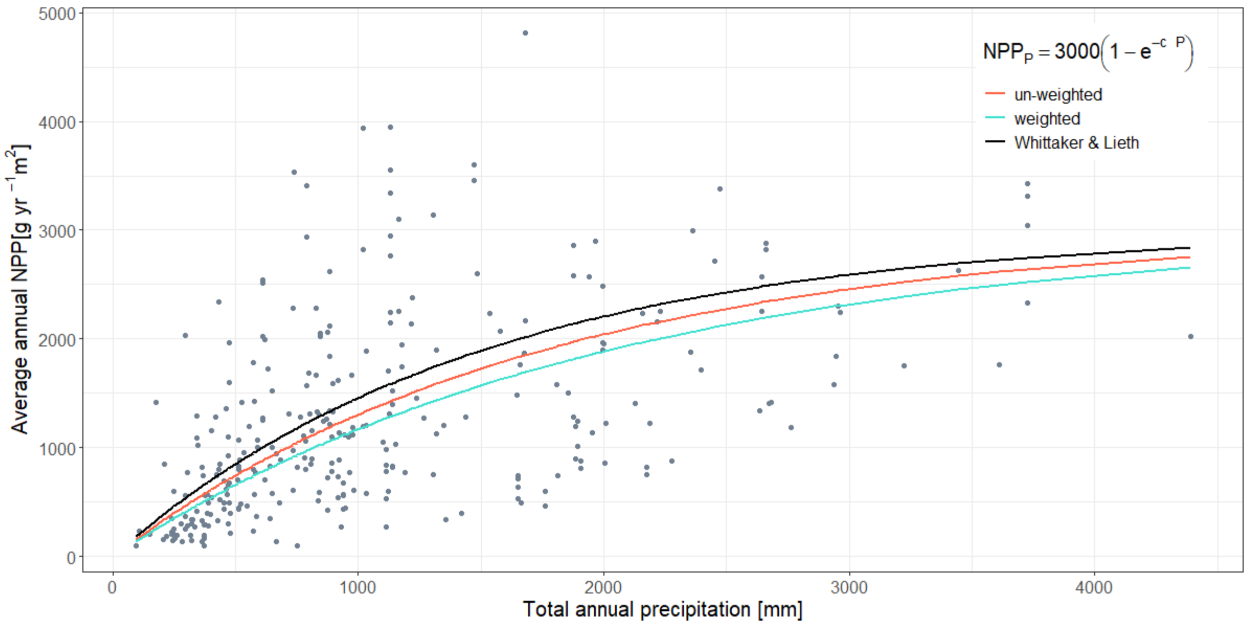

Figure A.2: The Miami Model. A non-linear fit of NPP vs. temperature fitted employing a weighted (blue) and un-weighted (red) non-linear regression. The fitting parameters, a and b, for the two fits are given in Table A.1.

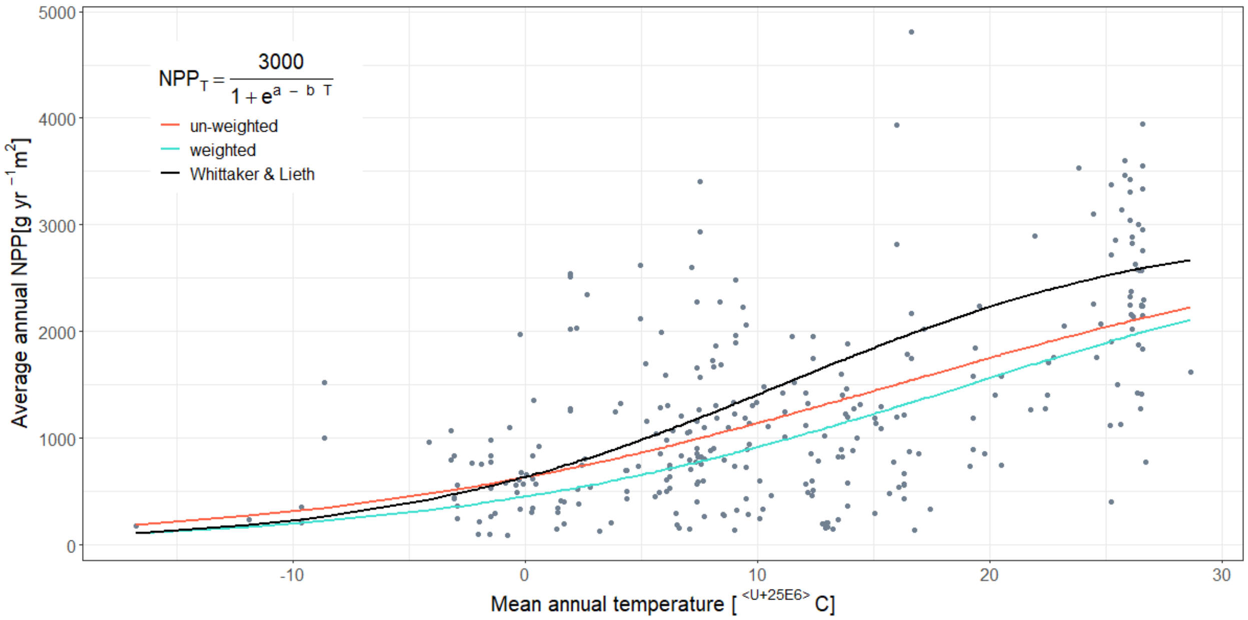

Table A.1: Fitting Parameters for the Miami Model based on our data and as given in ref. [1].

Parameter	Un-weighted	Weighted	Whittaker & Lieth
a	1.322244083	1.731620994	1.315
b	0.083074541	0.090813950	0.119
c	0.000570108	0.000494064	0.000664

Figure A.3: Plot of the percentage difference of $\boldsymbol{NPP_T}$ (**A**) and $\boldsymbol{NPP_P}$(**B**) as functions of mean annual temperature and total annual precipitation, respectively. $\boldsymbol{NPP_T}$ generally shows a greater difference between the two fits, with the error ranging from 5 to 40 %.

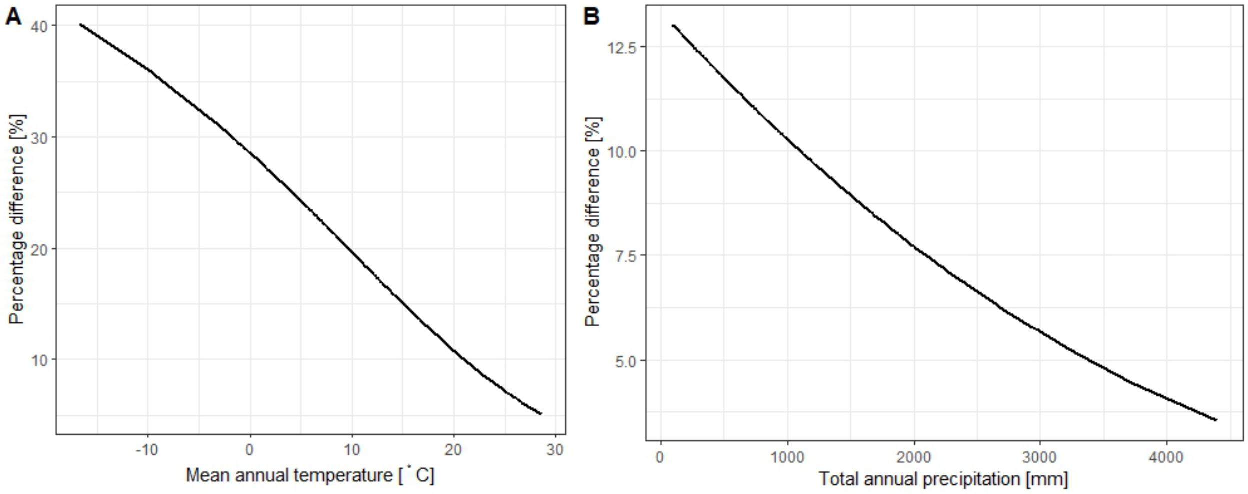

A.2 The biome specific variance

Table A.2: Absolute range of NPP ($gm^{-2}y^{-1}$), P_i^k, under the assumption that measurements are absent. Derived from the NPP estimates in the database using Eqn. (2), and rounded to nearest 10 grams.

Ecosystem type	P_i^{TNPP} [$gm^{-2}y^{-1}$]	P_i^{ANPP} [$gm^{-2}y^{-1}$]	P_i^{BNPP} [$gm^{-2}y^{-1}$]
Forest	1170	930	380
Grassland	970	550	600
Dry shrubland	380	120	300
Cropland	1000	830	180
Tundra	360	160	110
Peatland	500	240	370

References

1. Lieth, H (1975), 'Modeling the primary productivity of the world' in Lieth, H., and R. H. Whittaker [Eds.]. 1975. 'Primary productivity of the biosphere'. Ecological Studies, v. 14. Springer-Verlag, New York.
