# Supplementary material for: A global database of net primary production of terrestrial ecosystems
Source: Sci Data. 2025 Sep 2;12:1534. doi: 10.1038/s41597-025-05773-4 (PMC12405561; doi:10.1038/s41597-025-05773-4)
Supplement: Supplementary file 2 — Plotting routine for manuscript figures [file 41597_2025_5773_MOESM2_ESM.zip › Figures_plot.html]

Manuscript figures for ‘A global database of net primary production for the major terrestrial biomes’


Code 

- Show All Code
- Hide All Code
- Download Rmd

# Manuscript figures for ‘A global database of net primary production for the major terrestrial biomes’


```
#---- Load necessary libraries
library(readxl)
library(tidyverse)
library(ggplot2)
library(maps)
library(rnaturalearth)
library(latex2exp)
library(scales)
library(ggpubr)
library(grid)
library(nls2) 
library(gridExtra)
library(nlme)
library(plotbiomes)
```


```
site_info <- read_excel(paste(DB_file_path,'BiomassProduction_DB_V6.1.1_copy.xlsx', sep = ""), sheet = 1)
AG_methodology <- read_excel(paste(DB_file_path, 'BiomassProduction_DB_V6.1.1_copy.xlsx', sep = ""), sheet = 2)
AG_methods <- read_excel(paste(DB_file_path, 'BiomassProduction_DB_V6.1.1_copy.xlsx', sep = ""), sheet = 3)
BG_methodology <- read_excel(paste(DB_file_path, 'BiomassProduction_DB_V6.1.1_copy.xlsx', sep = ""), sheet = 4)
BG_methods <-  read_excel(paste(DB_file_path, 'BiomassProduction_DB_V6.1.1_copy.xlsx', sep = ""), sheet = 5)
NPP_estimates <- read_excel(paste(DB_file_path, 'BiomassProduction_DB_V6.1.1_copy.xlsx', sep =""), sheet = 6) %>%
  dplyr::select(-c(carbon_content, reference_1, reference_2, reference_3)) %>%
  left_join(AG_methodology[, c('AG_methodology_ID', 'RF')], by = 'AG_methodology_ID') %>%
  rename(RF_ANPP = RF) %>%
  left_join(BG_methodology[, c('BG_methodology_ID', 'RF')], by = 'BG_methodology_ID') %>%
  rename(RF_BNPP = RF) %>%
  left_join(site_info[,c('site_ID', 'biome_code')], by = 'site_ID') %>%
  distinct() %>%
  rename(biome = biome_code)

climate_data <- read_excel(paste(DB_file_path, 'BiomassProduction_DB_V6.1.1_copy.xlsx', sep =""), sheet = 12) %>%
  dplyr::select(c('site_ID', 'bio1', 'bio12')) %>%
  distinct()
```


```
#---- Define the color scheme
color_scheme <-   scale_color_manual(values = c("#db7c26", "#008000", "#a7c957","#5390d9" ,"#fdc500", '#bee9e8'))
```


## Whittaker-diagram


```
Whittaker_plot_data <- site_info[, c('site_ID', 'biome_code', 'climate_region', 'management_code')] %>%
  left_join(climate_data[, c('site_ID', 'bio1', 'bio12')], by = 'site_ID')%>%
  rename(temperature = bio1, precipitation = bio12) %>%
  rowwise() %>%
  mutate(precipitation =  precipitation/10) %>%
  mutate(biome_code = ifelse(biome_code == 'G', 'grassland', 
                             ifelse(biome_code =='F', 'forest', 
                                    ifelse(biome_code =='C', 'cropland',
                                           ifelse(biome_code == 'Sh', 'dry shrubland', 
                                                  ifelse(biome_code =='T', 'tundra',
                                                         ifelse(biome_code =='P', 'northern peatland', NA))))))) %>%
  mutate(Ecosystem_type = paste(climate_region, biome_code, sep = " ")) %>%
  mutate(Ecosystem_type = replace(Ecosystem_type,  biome_code == "northern peatland", "northern peatland")) %>%
  mutate(Ecosystem_type = replace(Ecosystem_type,  biome_code == "cropland", "cropland"))%>%
  mutate(Ecosystem_type = replace(Ecosystem_type,  biome_code == "dry shrubland", "dry shrubland"))%>%
  mutate(Ecosystem_type = replace(Ecosystem_type,  biome_code == "tundra", "tundra")) %>%
  filter(!is.na(climate_region)) %>%
  rename(`Biome` = 'biome_code') 

my_outliers <- get_outliers(tp = Whittaker_plot_data[, 5:6]) 

my_outliers_site_ID <- c('CA-tru-D01',
                         'IN-na2-D',
                         'IN-na3-D',
                         'IN-na3-D',
                         'IN-rlu-D01',
                         'IN-ush-D01',
                         'US-cas-D01',
                         'US-cas-D02',
                         'US-cas-D03',
                         'US-cas-D04'
)  

Whittaker_plot_data <- Whittaker_plot_data %>%
  mutate(status = ifelse(site_ID %in% my_outliers_site_ID, '#9b2226', '#4a4e69'))


my_palette <- colorRampPalette(colors = c("#62b6cb",  "#6a994e", "#ffba08"))(9)
```


```
whittaker_base_plot(color_palette = my_palette) +
  # add the temperature - precipitation data points
  geom_point(data = Whittaker_plot_data[Whittaker_plot_data$Biome != 'cropland',], 
             aes(x = temperature, 
                 y = precipitation,
                 shape = `Biome`), 
             size  = 3,
             #shape = 21,
             colour = Whittaker_plot_data[Whittaker_plot_data$Biome != 'cropland',]$status,
             stroke = 1.5,
             alpha = 0.75) + xlab("Temperature []") + ylab("Precipitation [cm]")  +
  theme_bw()  +
  theme(
    legend.justification = c(0, 1), # pick the upper left corner of the legend box and
    legend.position = c(0, 1), # adjust the position of the corner as relative to axis
    legend.background = element_rect(fill = NA), # transparent legend background
    legend.box = "horizontal", # horizontal arrangement of multiple legends
    legend.spacing.x = unit(0.5, units = "cm"), # horizontal spacing between legends
    panel.grid = element_blank(), # eliminate grids
    text=element_text(size=18)
  )
```


```
G3;Names for 'color_palette' either not specified or too few were specified. Using names from 'Ricklefs_colors'.
g
```

## Site location (geographical) plot


```
site_location <- site_info[, c('site_ID', 'latitude', 'north_south', 'longitude', 'east_west', 'climate_region', 'biome_code', 'elevation', 'management_code')] %>%
  rowwise() %>%
  mutate(management_code = replace(management_code, management_code == 'M', 'managed')) %>%
  mutate(management_code = replace(management_code, management_code == 'N', 'unmanaged')) %>%
  mutate(latitude = replace(latitude, north_south == 'S', -latitude)) %>%
  mutate(longitude = replace(longitude, east_west == 'W', -longitude)) %>%
  rename(Biome = biome_code, Climate = climate_region) 

world_map <- ggplot(maps::map('world',interior=FALSE,col='gray', fill=TRUE,lty=0), aes(x = long, y = lat, group = group)) +
  geom_polygon(fill='darkgray', colour = "darkgray") + xlab("Longitude") + ylab("Latitude") +
  geom_point(data = site_location, mapping = aes(x = longitude, y = latitude, fill = Biome), colour="black",pch=21, inherit.aes = FALSE, size = 3, )  +
  theme_bw() +
  scale_fill_manual(values = c("#db7c26", "#008000", "#a7c957","#5390d9" ,"#fdc500", '#bee9e8')) + theme(text=element_text(size=15))
```


```
plot(world_map)
```

## Measurement year distribution plot


```
measurement_year <- NPP_estimates %>%
  dplyr::select(site_ID, begin_year, end_year) %>%
  rowwise()%>%
  mutate(begin_year = replace(begin_year, begin_year == 9999, end_year)) %>% 
  mutate(year = map2(begin_year, end_year, ~seq(from = .x, to = .y, by = 1))) %>%
  unnest() %>%
  dplyr::select(c('site_ID', 'year')) %>%
  left_join(site_info[, c('site_ID', 'biome_code', 'climate_region')]) %>%
  distinct() %>%
  rename(Biome = biome_code)
```


```
G2;H2;Warningh: `cols` is now required when using `unnest()`.
ℹ Please use `cols = c(year)`.g
Joining with `by = join_by(site_ID)`
```


```
end_measurement_year <- NPP_estimates %>%
  dplyr::select(site_ID, end_year) %>%
  left_join(site_info[, c('site_ID', 'biome_code', 'climate_region')]) %>%
  distinct() %>%
  rename(Biome = biome_code)
```


```
Joining with `by = join_by(site_ID)`
```


```
ggplot(end_measurement_year, aes(x = end_year, fill = Biome)) + 
  annotate('rect', xmin=1964, xmax=1974, ymin=0, ymax=Inf, alpha=.5, fill='darkgray')+
  annotate("text", x=1969.2, y=40, label= "IBP",size = unit(6, "pt")) + geom_histogram(bins = 64, color = 'black') + 
  labs(y = 'Database record', x = 'End year of measurements')+
  theme_bw() +  scale_fill_manual(values = c("#db7c26", "#008000", "#a7c957","#5390d9" ,"#fdc500", '#bee9e8')) + theme(text=element_text(size=15)) + geom_segment(aes(x = 1966.7, y = 40, xend = 1964, yend = 40),
                  arrow = arrow(length = unit(0.2, "cm")), size = 1)+ theme(text=element_text(size=15)) + geom_segment(aes(x = 1971.7, y = 40, xend = 1974, yend = 40),
                  arrow = arrow(length = unit(0.2, "cm")), size = 1)
```


```
ggplot(site_info, aes(x=elevation, fill = biome_code))+ geom_histogram(bins = 50, color = 'black') + 
  labs(y = 'Database record', x = 'Elevation [m]', fill = 'Biome') +
  theme_bw() +  scale_fill_manual(values = c("#db7c26", "#008000", "#a7c957","#5390d9" ,"#fdc500", '#bee9e8')) + theme(text=element_text(size=15))
```

## Boxplots of NPP vs.biome and climate

### Compute site averaged ANPP, BNPP and TNPP and uncertainty


```
# Define PBik for each biome and component

PB_C_AG <- 830
PB_C_BG <- 180
PB_G_AG <- 550
PB_G_BG <- 600
PB_F_AG <- 930
PB_F_BG <- 380
PB_Sh_AG <- 120
PB_Sh_BG <- 300
PB_P_AG <- 240
PB_P_BG <- 370
PB_T_AG <- 160
PB_T_BG <- 110

PB_C_TNPP <- 1000
PB_G_TNPP <- 970
PB_F_TNPP <- 1170
PB_Sh_TNPP <- 380
PB_P_TNPP <- 500
PB_T_TNPP <- 360


#ANPP ----
## Cropland and Grassland
ANPP_crop_grass <- NPP_estimates %>%
  filter(biome %in% c('G', 'C')) %>%
  dplyr::select('biome', 'site_ID', 'begin_year', 'end_year', 'herbs', 'shrubs', 
                'ANPP_herbs_shrubs','ANPP_non_tree' ,'ANPP_tree', 'herbivory', 'reproductive_material',
                'RF_ANPP') %>%
  mutate(ts_length = 1 + (end_year - begin_year)) %>%
  mutate(ts_length = replace(ts_length, begin_year == 9999, 1)) %>%
  rowwise() %>%
  mutate(ANPP_non_tree = replace(ANPP_non_tree,is.na(ANPP_non_tree) & !(is.na(shrubs) & is.na(herbs)), sum(herbs, shrubs, na.rm = T))) %>%
  mutate(ANPP = ifelse(!(is.na(ANPP_non_tree) & is.na(ANPP_tree)), sum(ANPP_non_tree, ANPP_tree, herbivory, na.rm = T), NA)) %>%
  mutate(uncertainty_AG = ifelse(biome == 'C', PB_C_AG*RF_ANPP/sqrt(ts_length),
                                 ifelse(biome == 'G', PB_G_AG*RF_ANPP/sqrt(ts_length), NA))) %>%
  filter(!is.na(ANPP)) %>%
  dplyr::left_join(data.frame(table(NPP_estimates = .[,c('site_ID')]))) %>%
  rename(ANPP_count = Freq) %>%
  group_by(site_ID) %>%
  reframe(ANPP = mean(ANPP, na.rm = T), 
          uncertainty_AG = sqrt(sum((uncertainty_AG)^2, na.rm = T))/ANPP_count) %>%
  distinct()
```


```
Joining with `by = join_by(site_ID)`
```


```
## Peatland and Tundra
ANPP_peat_tund <- NPP_estimates %>%
  filter(biome %in% c('P', 'T')) %>%
  dplyr::select('biome', 'site_ID', 'begin_year', 'end_year', 'ANPP_herbs_shrubs', 'non_vascular' ,'ANPP_non_tree' ,'ANPP_tree',
                'RF_ANPP') %>%
  filter(!(is.na(ANPP_herbs_shrubs) & is.na(non_vascular) & is.na(ANPP_non_tree) & is.na(ANPP_tree))) %>%
  filter(!is.na(RF_ANPP)) %>%
  dplyr::left_join(data.frame(table(NPP_estimates = .[,c('site_ID')]))) %>%
  rename(ANPP_count = Freq) %>%
  mutate(ts_length = 1 + (end_year - begin_year)) %>%
  mutate(ts_length = replace(ts_length, begin_year == 9999, 1)) %>%
  rowwise() %>%
  mutate(ANPP_non_tree = replace(ANPP_non_tree, is.na(ANPP_non_tree) & !(is.na(ANPP_herbs_shrubs) | is.na(non_vascular)), sum(ANPP_herbs_shrubs, non_vascular, na.rm = T))) %>%
  mutate(ANPP_non_tree = replace(ANPP_non_tree, is.na(ANPP_non_tree) & site_ID %in% c('SE-kar-D01_F', 'SE-kar-D01_C'),
                                 sum(ANPP_herbs_shrubs, non_vascular, na.rm = T))) %>%
  mutate(ANPP = ifelse(!is.na(ANPP_non_tree), sum(ANPP_non_tree, ANPP_tree, na.rm = T), 
                       ifelse(is.na(ANPP_non_tree) & ANPP_count == 1 , sum(ANPP_herbs_shrubs, non_vascular, na.rm = T), NA))) %>%
  mutate(uncertainty_AG = ifelse(biome == 'P', PB_P_AG*RF_ANPP/sqrt(ts_length),
                                 ifelse(biome == 'T', PB_T_AG*RF_ANPP/sqrt(ts_length), NA))) %>%
  mutate(uncertainty_herbs_shrubs =  ifelse(is.na(ANPP) &
                                              !is.na(ANPP_herbs_shrubs), uncertainty_AG , NA)) %>%
  group_by(site_ID) %>%
  reframe(ANPP = mean(ANPP, na.rm = T), 
          ANPP_non_vascular = mean(non_vascular, na.rm = T), 
          ANPP_herbs_shrubs = mean(ANPP_herbs_shrubs, na.rm = T), 
          uncertainty_AG = sqrt(sum((uncertainty_AG)^2, na.rm = T))/ANPP_count,
          uncertainty_herbs_shrubs = sqrt(sum((uncertainty_herbs_shrubs)^2, na.rm = T))) %>%
  distinct() %>%
  mutate_if(is.numeric, list(~na_if(., 0.0000)))%>%
  mutate_if(is.numeric, list(~na_if(., NaN))) %>%
  rowwise() %>%
  mutate(uncertainty_AG = replace(uncertainty_AG, is.na(ANPP), uncertainty_herbs_shrubs)) %>%
  mutate(ANPP = replace(ANPP, is.na(ANPP), sum(ANPP_non_vascular, ANPP_herbs_shrubs, na.rm = T))) %>%
  distinct()
```


```
Joining with `by = join_by(site_ID)`
```


```
## Forest and Shrubland 
ANPP_fore_shrub <- NPP_estimates %>%
  filter(biome %in% c('F', 'Sh')) %>%
  dplyr::select('biome', 'site_ID', 'begin_year', 'end_year', 'ANPP_non_tree', 'stem', 'branch', 
                'ANPP_wood', 'ANPP_tot_litter', 'ANPP_tree', 'herbivory', 'reproductive_material',
                'RF_ANPP') %>%
  filter(!(is.na(ANPP_non_tree) & is.na(stem) & is.na(ANPP_wood) & is.na(ANPP_tot_litter) & 
             is.na(ANPP_tree) & is.na(herbivory) & is.na(reproductive_material))) %>%
  dplyr::left_join(data.frame(table(NPP_estimates = .[,c('site_ID')]))) %>%
  rename(ANPP_count = Freq) %>%
  mutate(ANPP_count = replace(ANPP_count, site_ID == 'JP-Ta1-D01', 7)) %>%
  mutate(ANPP_count = replace(ANPP_count, site_ID == 'US-Dk3-F01', 1)) %>%
  rowwise() %>%
  mutate(ts_length = 1 + (end_year - begin_year)) %>%
  mutate(ts_length = replace(ts_length, begin_year == 9999, 1)) %>%
  mutate(ANPP_wood = replace(ANPP_wood, is.na(ANPP_wood) & !is.na(stem), sum(stem, branch, na.rm = T))) %>%
  mutate(ANPP_tree = replace(ANPP_tree, is.na(ANPP_tree) & !is.na(ANPP_wood), sum(ANPP_wood, ANPP_tot_litter, na.rm = T))) %>%
  mutate(uncertainty_AG = ifelse(biome == 'F', PB_F_AG*RF_ANPP/sqrt(ts_length),
                                 ifelse(biome == 'S', PB_Sh_AG*RF_ANPP/sqrt(ts_length), NA))) %>%
  group_by(site_ID) %>%
  reframe(ANPP_understory = mean(ANPP_non_tree, na.rm = T),
          ANPP_tree = mean(ANPP_tree, na.rm = T), 
          ANPP_reproductive_material = mean(reproductive_material, na.rm = T), 
          ANPP_herbivory = mean(herbivory, na.rm = T), 
          uncertainty_AG = sqrt(sum((uncertainty_AG)^2, na.rm = T))/ANPP_count) %>%
  distinct() %>%
  mutate_if(is.numeric, list(~na_if(., NaN))) %>%
  rowwise() %>%
  mutate(ANPP = sum(ANPP_understory, ANPP_tree, ANPP_reproductive_material, 
                    ANPP_herbivory, na.rm = T))  %>%
  distinct()
```


```
Joining with `by = join_by(site_ID)`
```


```
#BNPP ----
## Cropland and Grassland
BNPP_crop_grass <- NPP_estimates %>%
  filter(biome %in% c('G', 'C')) %>%
  dplyr::select('biome', 'site_ID', 'begin_year', 'end_year', 'BNPP',
                'RF_BNPP') %>%
  filter(!is.na(BNPP)) %>%
  dplyr::left_join(data.frame(table(NPP_estimates = .[,c('site_ID')]))) %>%
  rename(BNPP_count = Freq) %>%
  mutate(ts_length = 1 + (end_year - begin_year)) %>%
  mutate(ts_length = replace(ts_length, begin_year == 9999, 1)) %>%
  rowwise() %>%
  mutate(uncertainty_BG = ifelse(biome == 'C', PB_C_BG*RF_BNPP/sqrt(ts_length),
                                 ifelse(biome == 'G', PB_G_BG*RF_BNPP/sqrt(ts_length), NA))) %>%
  group_by(site_ID) %>%
  reframe(BNPP = mean(BNPP, na.rm = T), 
          uncertainty_BG = sqrt(sum((uncertainty_BG)^2, na.rm = T))/BNPP_count) %>%
  distinct()
```


```
Joining with `by = join_by(site_ID)`
```


```
## Peatland and Tundra
BNPP_peat_tund <- NPP_estimates %>%
  filter(biome %in% c('P', 'T')) %>%
  dplyr::select('biome', 'site_ID', 'begin_year', 'end_year', 'fine_roots', 'coarse_roots','BNPP',
                'RF_BNPP') %>%
  filter(!(is.na(BNPP) & is.na(fine_roots) & is.na(coarse_roots))) %>%
  filter(!is.na(RF_BNPP)) %>%
  dplyr::left_join(data.frame(table(NPP_estimates = .[,c('site_ID')]))) %>%
  rename(BNPP_count = Freq) %>%
  mutate(ts_length = 1 + (end_year - begin_year)) %>%
  mutate(ts_length = replace(ts_length, begin_year == 9999, 1)) %>%
  rowwise() %>%
  mutate(BNPP = replace(BNPP, is.na(BNPP) & !is.na(fine_roots), sum(coarse_roots,fine_roots, na.rm =T))) %>%
  mutate(uncertainty_BG = ifelse(biome == 'P', PB_P_BG*RF_BNPP/sqrt(ts_length),
                                 ifelse(biome == 'T', PB_T_BG*RF_BNPP/sqrt(ts_length), NA))) %>%
  group_by(site_ID) %>%
  reframe(BNPP = mean(BNPP, na.rm = T), 
          uncertainty_BG = sqrt(sum((uncertainty_BG)^2, na.rm = T))/BNPP_count) %>%
  distinct() %>%
  mutate_if(is.numeric, list(~na_if(., 0.0000)))%>%
  mutate_if(is.numeric, list(~na_if(., NaN)))
```


```
Joining with `by = join_by(site_ID)`
```


```
## Forest and Shrubland 
BNPP_fore_shrub <- NPP_estimates %>%
  filter(biome %in% c('F', 'Sh')) %>%
  dplyr::select('biome', 'site_ID', 'begin_year', 'end_year', 'fine_roots', 'coarse_roots','BNPP',
                'RF_BNPP') %>%
  filter(!(is.na(BNPP) & is.na(fine_roots))) %>%
  filter(!is.na(RF_BNPP)) %>%
  dplyr::left_join(data.frame(table(NPP_estimates = .[,c('site_ID')]))) %>%
  rename(BNPP_count = Freq) %>%
  mutate(ts_length = 1 + (end_year - begin_year)) %>%
  mutate(ts_length = replace(ts_length, begin_year == 9999, 1)) %>%
  rowwise() %>%
  mutate(BNPP = replace(BNPP, is.na(BNPP) & !is.na(fine_roots), sum(coarse_roots,fine_roots, na.rm =T))) %>%
  mutate(uncertainty_BG = ifelse(biome == 'F', PB_F_BG*RF_BNPP/sqrt(ts_length),
                                 ifelse(biome == 'Sh', PB_Sh_BG*RF_BNPP/sqrt(ts_length), NA))) %>%
  group_by(site_ID) %>%
  reframe(BNPP = mean(BNPP, na.rm = T), 
          uncertainty_BG = sqrt(sum((uncertainty_BG)^2, na.rm = T))/BNPP_count) %>%
  distinct() %>%
  mutate_if(is.numeric, list(~na_if(., 0.0000))) %>%
  mutate_if(is.numeric, list(~na_if(., NaN)))
```


```
Joining with `by = join_by(site_ID)`
```


```
ANPP_all <- ANPP_fore_shrub[, c('site_ID', 'ANPP', 'uncertainty_AG')] %>%
  bind_rows(ANPP_crop_grass[, c('site_ID', 'ANPP', 'uncertainty_AG')]) %>%
  bind_rows(ANPP_peat_tund[, c('site_ID', 'ANPP', 'uncertainty_AG')]) 

BNPP_all <- BNPP_fore_shrub[, c('site_ID', 'BNPP', 'uncertainty_BG')] %>%
  bind_rows(BNPP_crop_grass[, c('site_ID', 'BNPP', 'uncertainty_BG')]) %>%
  bind_rows(BNPP_peat_tund[, c('site_ID', 'BNPP', 'uncertainty_BG')]) 

#NPP ----
NPP <- ANPP_all %>%
  left_join(BNPP_all, by = 'site_ID') %>%
  distinct() %>%
  mutate(uncertainty_TNPP = sqrt(sum(uncertainty_AG^2,uncertainty_BG^2))) %>%
  mutate(TNPP = sum(ANPP, BNPP, na.rm = F)) 

NPP_other <- NPP_estimates %>%
  filter(is.na(fine_roots) & is.na(BNPP)) %>%
  filter(is.na(herbs) & is.na(shrubs) & is.na(ANPP_herbs_shrubs) & is.na(non_vascular)
         & is.na(stem) & is.na(ANPP_wood) & is.na(ANPP_tot_litter) & is.na(ANPP_tree)) %>%
  filter(!(is.na(NPP_tot1) & is.na(NPP_tot2))) %>%
  filter(!site_ID %in% c(NPP$site_ID)) %>%
  dplyr::left_join(data.frame(table(NPP_estimates = .[,c('site_ID')]))) %>%
  rename(NPP_count = Freq) %>%
  mutate(ts_length = 1 + (end_year - begin_year)) %>%
  mutate(ts_length = replace(ts_length, begin_year == 9999, 1)) %>%
  rowwise() %>%
  mutate(NPP_tot2 = replace(NPP_tot2, is.na(NPP_tot2), sum(NPP_tot1, reproductive_material, herbivory, na.rm = T))) %>%
  mutate(uncertainty_TNPP = ifelse(biome == 'F' & AG_methodology_ID %in% c('TBP1', 'TBP2', 'TBP3', 'TBP4'), PB_F_TNPP*RF_BNPP/sqrt(ts_length),
                                   ifelse(biome == 'G' & AG_methodology_ID %in% c('TBP1', 'TBP2', 'TBP3', 'TBP4'), PB_G_TNPP*RF_BNPP/sqrt(ts_length), 
                                          ifelse(biome == 'C' & AG_methodology_ID %in% c('TBP1', 'TBP2', 'TBP3', 'TBP4'), PB_C_TNPP*RF_BNPP/sqrt(ts_length), 
                                                 ifelse(biome == 'P' & AG_methodology_ID %in% c('TBP1', 'TBP2', 'TBP3', 'TBP4'), PB_P_TNPP*RF_BNPP/sqrt(ts_length), 
                                                        ifelse(biome == 'T' & AG_methodology_ID %in% c('TBP1', 'TBP2', 'TBP3', 'TBP4'), PB_T_TNPP*RF_BNPP/sqrt(ts_length), NA)))))) %>%
  mutate(uncertainty_AG = ifelse(biome == 'F', PB_F_AG*RF_ANPP/sqrt(ts_length),
                                 ifelse(biome == 'S', PB_Sh_AG*RF_ANPP/sqrt(ts_length), 
                                        ifelse(biome == 'C', PB_C_AG*RF_ANPP/sqrt(ts_length),
                                               ifelse(biome == 'G', PB_G_AG*RF_ANPP/sqrt(ts_length),
                                                      ifelse(biome == 'P', PB_P_AG*RF_ANPP/sqrt(ts_length),
                                                             ifelse(biome == 'T', PB_T_AG*RF_ANPP/sqrt(ts_length), NA))))))) %>%
  mutate(uncertainty_BG = ifelse(biome == 'F', PB_F_BG*RF_BNPP/sqrt(ts_length),
                                 ifelse(biome == 'S', PB_Sh_BG*RF_BNPP/sqrt(ts_length), 
                                        ifelse(biome == 'C', PB_C_BG*RF_BNPP/sqrt(ts_length),
                                               ifelse(biome == 'G', PB_G_BG*RF_BNPP/sqrt(ts_length),
                                                      ifelse(biome == 'P', PB_P_BG*RF_BNPP/sqrt(ts_length),
                                                             ifelse(biome == 'T', PB_T_BG*RF_BNPP/sqrt(ts_length), NA))))))) %>%
  mutate(uncertainty_TNPP = replace(uncertainty_TNPP, is.na(uncertainty_TNPP),
                                    sqrt(sum(uncertainty_AG^2,uncertainty_BG^2)))) %>%
  group_by(site_ID) %>%
  reframe(TNPP = mean(NPP_tot2, na.rm = T), 
          uncertainty_TNPP = sqrt(sum((uncertainty_TNPP)^2, na.rm = T))/NPP_count) %>%
  distinct() %>%
  mutate_if(is.numeric, list(~na_if(., NaN)))
```


```
Joining with `by = join_by(site_ID)`
```


```
NPP_all <- NPP %>%
  bind_rows(NPP_other) %>%
  left_join(site_info[,c('site_ID', 'biome_code', 'climate_region', 'management_code')])
```


```
Joining with `by = join_by(site_ID)`
```


```
NPP_by_biome <- NPP_all[c('site_ID', 'biome_code', 'ANPP', 'BNPP', 'TNPP')] %>%
  pivot_longer(3:5, values_to = 'NPP', names_to = 'type')

NPP_by_climate <- NPP_all[c('site_ID', 'climate_region', 'ANPP', 'BNPP', 'TNPP')] %>%
  pivot_longer(3:5, values_to = 'NPP', names_to = 'type')
  
  boxplot_biome <-ggplot(na.omit(NPP_by_biome), aes(x=biome_code, y=NPP, fill=type)) +
  geom_boxplot() +  
  labs(y =  TeX(r'(Average annual NPP   [g m$^{-2}$yr$^{-1}])'), x = 'Biome', fill = ' ') +
  scale_fill_manual(values = c("#a7c957", "#7f5539",  "#ffd60a")) +
  theme_bw() +geom_boxplot(lwd=1) + theme(text=element_text(size=15))

boxplot_climate <-ggplot(na.omit(NPP_by_climate), aes(x=climate_region, y=NPP, fill=type)) +
  geom_boxplot() +  
  labs(y = TeX(r'(Average annual NPP   [g m$^{-2}$yr$^{-1}])'), x = 'Climate Region', fill = ' ') +
  scale_fill_manual(values = c("#a7c957", "#7f5539",  "#ffd60a")) + 
  theme_bw() +geom_boxplot(lwd=1) + theme(text=element_text(size=15))
```


```
plot(boxplot_climate)
```


```
plot(boxplot_biome)
```

## The Miami model


```
normalization_constant <- sum(na.omit(NPP_all[NPP_all$management_code == 'N',])$uncertainty_TNPP)

NPP_Miami_Model_temp <- na.omit(NPP_all[, c('site_ID','TNPP', 'management_code', 'uncertainty_TNPP')]) %>%
  filter(management_code == 'N') %>%
  left_join(climate_data, by = 'site_ID') %>%
  mutate(uncertainty_normalized = uncertainty_TNPP/normalization_constant) %>%
  arrange(bio1) %>%
  dplyr::select(-c(site_ID))

NPP_Miami_Model_prec <- na.omit(NPP_all[, c('site_ID', 'TNPP', 'management_code', 'uncertainty_TNPP')]) %>%
  filter(management_code == 'N') %>%
  left_join(climate_data, by = 'site_ID') %>%
  mutate(uncertainty_normalized = uncertainty_TNPP/normalization_constant ) %>%
  arrange(bio12) %>%
  dplyr::select(-c(site_ID))


MiamiModel_temp <- nls2(TNPP ~ 3000/(1+exp(a-b*bio1)), data = na.omit(NPP_Miami_Model_temp), list(a = 1.315, b = 0.119))
MiamiModel_prec <- nls2(TNPP ~ 3000*(1-exp(-a*bio12)), data = na.omit(NPP_Miami_Model_prec), list(a = 0.000664))


MiamiModel_temp_weighted <-  nls2(TNPP ~ 3000/(1+exp(a-b*bio1)), data = na.omit(NPP_Miami_Model_temp), list(a = 1.315, b = 0.119), control = nls.control(maxiter = 1000000, minFactor = 0.000000000006), weights = 1/uncertainty_normalized)
MiamiModel_prec_weighted <- nls2(TNPP ~ 3000*(1-exp(-a*bio12)), data = na.omit(NPP_Miami_Model_prec), list(a = 0.000664), weights = 1/uncertainty_normalized)

func_MiamiModel_temp <- function(a,b,temp){
  res = c()
  for (t in temp){
    res_t = 3000/(1+exp(a-b*t))
    res = c(res, res_t)
  }
  
  return(res)
}

func_MiamiModel_prec <- function(c,prec){
  res = c()
  for (p in prec){
    res_p = 3000*(1-exp(-c*p))
    res = c(res, res_p)
  }
  return(res)
}

Whittaker_Lieth_temp <- func_MiamiModel_temp(1.315, 0.119, na.omit(NPP_Miami_Model_temp$bio1)) 
Whittaker_Lieth_prec <- func_MiamiModel_prec(0.000664, na.omit(NPP_Miami_Model_prec$bio12))

ggplot( data = na.omit(NPP_Miami_Model_temp), aes(x = bio1, y = TNPP)) +geom_point(color = 'slategrey') +
  labs(x = TeX(r'(Mean annual temperature $[^\circ C]$)'), y = TeX(r'(Average annual NPP [g yr $^{-1}$m$^2$])')) +
  geom_segment(aes(x = bio1, xend = dplyr::lead(bio1), yend = dplyr::lead(predict(MiamiModel_temp, newdata = na.omit(NPP_Miami_Model_temp))), y = predict(MiamiModel_temp, newdata = na.omit(NPP_Miami_Model_temp)), color = "un-weighted"), linewidth = 1, linetype= 1) +
  geom_segment(aes(x = bio1, xend = dplyr::lead(bio1), yend = dplyr::lead(predict(MiamiModel_temp_weighted, newdata = na.omit(NPP_Miami_Model_temp))), y = predict(MiamiModel_temp_weighted, newdata = na.omit(NPP_Miami_Model_temp)), color = "weighted"), linewidth = 1, linetype = 1) +
  geom_segment(aes(x = bio1, xend = dplyr::lead(bio1), yend = dplyr::lead(na.omit(Whittaker_Lieth_temp)), y = na.omit(Whittaker_Lieth_temp) , color = 'Whittaker & Lieth'), linewidth = 1, linetype= 1) +
  scale_colour_manual(TeX(r'($NPP_T = \frac{3000}{1+e^{a\;-\;b\; T}}$    )'), 
                      breaks = c("un-weighted", "weighted", "Whittaker & Lieth"),
                      values = c("tomato", "turquoise", "black")) + theme_bw() +
  theme(legend.position = c(0.15, 0.8), legend.key = element_blank()) + theme(text=element_text(size=15))
```


```
ggplot( data = na.omit(NPP_Miami_Model_prec), aes(x = bio12, y = TNPP)) +geom_point(color = 'slategrey') +
  geom_segment(aes(x = bio12, xend = dplyr::lead(bio12), yend = dplyr::lead(predict(MiamiModel_prec, newdata = na.omit(NPP_Miami_Model_prec))), y = predict(MiamiModel_prec, newdata = na.omit(NPP_Miami_Model_prec)),color = "un-weighted"), linewidth = 1, linetype= 1) +
  geom_segment(aes(x = bio12, xend = dplyr::lead(bio12), yend = dplyr::lead(predict(MiamiModel_prec_weighted, newdata = na.omit(NPP_Miami_Model_prec))), y = predict(MiamiModel_prec_weighted, newdata = na.omit(NPP_Miami_Model_prec)),color = "weighted"), linewidth = 1, linetype = 1) +
  geom_segment(aes(x = bio12, xend = dplyr::lead(bio12), yend = dplyr::lead(na.omit(Whittaker_Lieth_prec)), y = na.omit(Whittaker_Lieth_prec) , color = 'Whittaker & Lieth'), linewidth = 1, linetype= 1) +
  labs(x = 'Total annual precipitation [mm]', y = TeX(r'(Average annual NPP [g yr $^{-1}$m$^2$])'), colour = "Weighted:") +
  scale_colour_manual(TeX(r'($NPP_P = 3000 \left(1-e^{-c \;P}\right)$       )'), 
                      breaks = c("un-weighted", "weighted", "Whittaker & Lieth"),
                      values = c("tomato", "turquoise", "black")) + theme_bw() +
  theme(legend.position = c(0.87, 0.85)) + theme(text=element_text(size=15))
```


```
model_coefficents <- data.frame('Parameter' = c('a','b','c'),
                                'Un.weighted' = c(summary(MiamiModel_temp)$coef[, c('Estimate')][1],summary(MiamiModel_temp)$coef[, c('Estimate')][2],summary(MiamiModel_prec)$coef[, c('Estimate')][1]),
                                'Weighted' = c(summary(MiamiModel_temp_weighted)$coef[, c('Estimate')][1], summary(MiamiModel_temp_weighted)$coef[, c('Estimate')][2], summary(MiamiModel_prec_weighted)$coef[, c('Estimate')][1]))
```

# Calculating maximum and minimum percentage difference


```
predict_temp_unweighted <- predict(MiamiModel_temp, newdata = na.omit(NPP_Miami_Model_temp))
predict_temp_weighted <- predict(MiamiModel_temp_weighted, newdata = na.omit(NPP_Miami_Model_temp))
predict_prec_unweighted <- predict(MiamiModel_prec, newdata = na.omit(NPP_Miami_Model_prec))
predict_prec_weighted <- predict(MiamiModel_prec_weighted, newdata = na.omit(NPP_Miami_Model_prec))


predicted_values_temp_fit <- data.frame(NPP_temp_fit = predict_temp_unweighted,
                                        NPP_temp_fit_weighted = predict_temp_weighted) %>%
  bind_cols(temp = na.omit(NPP_Miami_Model_temp)$bio1) %>%
  rowwise() %>%
  mutate(NPP_temp_fit_diff =  NPP_temp_fit-NPP_temp_fit_weighted) %>%
  mutate(NPP_temp_fit_perc_diff = (abs(NPP_temp_fit_diff)/((NPP_temp_fit)))*100) 

predicted_values_prec_fit <- data.frame(NPP_prec_fit = predict_prec_unweighted,
                                        NPP_prec_fit_weighted = predict_prec_weighted) %>%
  bind_cols(prec = na.omit(NPP_Miami_Model_prec)$bio12) %>%
  rowwise() %>%
  mutate(NPP_prec_fit_diff =  NPP_prec_fit-NPP_prec_fit_weighted) %>%
  mutate(NPP_prec_fit_perc_diff = (abs(NPP_prec_fit_diff)/((NPP_prec_fit)))*100)

perc_diff_temp_fits <- ggplot(data = predicted_values_temp_fit) +
  geom_segment(aes(x = temp, xend = dplyr::lead(temp), yend = dplyr::lead(NPP_temp_fit_perc_diff), y = NPP_temp_fit_perc_diff), linewidth = 1, linetype= 1) +
  labs(x = TeX(r'(Mean annual temperature $[^\circ C]$)'),
       y = 'Percentage difference [%]')  + theme_bw()
```


```
Warning in sub("^ +", " ", ch) :
  unable to translate '    phantom() ~ "<e2>\u0097<a6>" ~ phantom()' to a wide string
```


```
perc_diff_prec_fits <- ggplot(data = predicted_values_prec_fit) +
  geom_segment(aes(x = prec, xend = dplyr::lead(prec), yend = dplyr::lead(NPP_prec_fit_perc_diff), y = NPP_prec_fit_perc_diff ), linewidth = 1, linetype= 1) +
  labs(x = 'Total annual precipitation [mm]',
       y = 'Percentage difference [%]')  + theme_bw()


ggarrange(perc_diff_temp_fits, perc_diff_prec_fits,
          labels = c("A", "B"),
          ncol = 2, nrow = 1,
          widths = c(0.85, 1))
```


```
G2;H2;Warningh: Removed 1 row containing missing values or values outside the scale range (`geom_segment()`).g
G2;H2;Warningh: Removed 1 row containing missing values or values outside the scale range (`geom_segment()`).g
```


```
rs_p1 <- plot(MiamiModel_prec_weighted, title = "bl")
qq_p1 <- qqnorm(MiamiModel_prec_weighted, abline = c(0,1))
rs_p2 <- plot(MiamiModel_prec)
qq_p2 <- qqnorm(MiamiModel_prec, abline = c(0,1))
grid.arrange(arrangeGrob(rs_p1, top = 'weighted'),
             arrangeGrob(qq_p1, top = ' '),
             arrangeGrob(rs_p2, top =  'un-weighted'), 
             arrangeGrob(qq_p2, top = ' '),
             top = textGrob(TeX(r'(Diagnostic plots $NPP_P$)'),gp=gpar(fontsize=20,font=3)), ncol=2)
```


```
rs_p3 <- plot(MiamiModel_temp_weighted)
qq_p3 <- qqnorm(MiamiModel_temp_weighted, abline = c(0,1))
rs_p4 <- plot(MiamiModel_temp)
qq_p4 <- qqnorm(MiamiModel_temp, abline = c(0,1))
grid.arrange(arrangeGrob(rs_p3, top = 'weighted'),
             arrangeGrob(qq_p3, top = ' '),
             arrangeGrob(rs_p4, top =  'un-weighted'), 
             arrangeGrob(qq_p4, top = ' '),
             top = textGrob(TeX(r'(Diagnostic plots $NPP_T$)'),gp=gpar(fontsize=20,font=3)), ncol=2)
```

LS0tDQp0aXRsZTogIk1hbnVzY3JpcHQgZmlndXJlcyBmb3IgJ0EgZ2xvYmFsIGRhdGFiYXNlIG9mIG5ldCBwcmltYXJ5IHByb2R1Y3Rpb24gZm9yIHRoZSBtYWpvciB0ZXJyZXN0cmlhbCBiaW9tZXMnIg0Kb3V0cHV0OiBodG1sX25vdGVib29rDQotLS0NCg0KYGBge3IsIG1lc3NhZ2U9RkFMU0V9DQojLS0tLSBMb2FkIG5lY2Vzc2FyeSBsaWJyYXJpZXMNCmxpYnJhcnkocmVhZHhsKQ0KbGlicmFyeSh0aWR5dmVyc2UpDQpsaWJyYXJ5KGdncGxvdDIpDQpsaWJyYXJ5KG1hcHMpDQpsaWJyYXJ5KHJuYXR1cmFsZWFydGgpDQpsaWJyYXJ5KGxhdGV4MmV4cCkNCmxpYnJhcnkoc2NhbGVzKQ0KbGlicmFyeShnZ3B1YnIpDQpsaWJyYXJ5KGdyaWQpDQpsaWJyYXJ5KG5sczIpIA0KbGlicmFyeShncmlkRXh0cmEpDQpsaWJyYXJ5KG5sbWUpDQpsaWJyYXJ5KHBsb3RiaW9tZXMpDQpgYGANCg0KYGBge3IsZWNobz1GQUxTRX0NCkRCX2ZpbGVfcGF0aCA8LSAiRTpcXEZpbmFsRGF0YWJhc2UzXFxTdWJtaXNzaW9uXFxSZXN1Ym1pc3Npb25cXCINCmBgYA0KDQpgYGB7cn0NCnNpdGVfaW5mbyA8LSByZWFkX2V4Y2VsKHBhc3RlKERCX2ZpbGVfcGF0aCwnQmlvbWFzc1Byb2R1Y3Rpb25fREJfVjYuMS4xX2NvcHkueGxzeCcsIHNlcCA9ICIiKSwgc2hlZXQgPSAxKQ0KQUdfbWV0aG9kb2xvZ3kgPC0gcmVhZF9leGNlbChwYXN0ZShEQl9maWxlX3BhdGgsICdCaW9tYXNzUHJvZHVjdGlvbl9EQl9WNi4xLjFfY29weS54bHN4Jywgc2VwID0gIiIpLCBzaGVldCA9IDIpDQpBR19tZXRob2RzIDwtIHJlYWRfZXhjZWwocGFzdGUoREJfZmlsZV9wYXRoLCAnQmlvbWFzc1Byb2R1Y3Rpb25fREJfVjYuMS4xX2NvcHkueGxzeCcsIHNlcCA9ICIiKSwgc2hlZXQgPSAzKQ0KQkdfbWV0aG9kb2xvZ3kgPC0gcmVhZF9leGNlbChwYXN0ZShEQl9maWxlX3BhdGgsICdCaW9tYXNzUHJvZHVjdGlvbl9EQl9WNi4xLjFfY29weS54bHN4Jywgc2VwID0gIiIpLCBzaGVldCA9IDQpDQpCR19tZXRob2RzIDwtICByZWFkX2V4Y2VsKHBhc3RlKERCX2ZpbGVfcGF0aCwgJ0Jpb21hc3NQcm9kdWN0aW9uX0RCX1Y2LjEuMV9jb3B5Lnhsc3gnLCBzZXAgPSAiIiksIHNoZWV0ID0gNSkNCk5QUF9lc3RpbWF0ZXMgPC0gcmVhZF9leGNlbChwYXN0ZShEQl9maWxlX3BhdGgsICdCaW9tYXNzUHJvZHVjdGlvbl9EQl9WNi4xLjFfY29weS54bHN4Jywgc2VwID0iIiksIHNoZWV0ID0gNikgJT4lDQogIGRwbHlyOjpzZWxlY3QoLWMoY2FyYm9uX2NvbnRlbnQsIHJlZmVyZW5jZV8xLCByZWZlcmVuY2VfMiwgcmVmZXJlbmNlXzMpKSAlPiUNCiAgbGVmdF9qb2luKEFHX21ldGhvZG9sb2d5WywgYygnQUdfbWV0aG9kb2xvZ3lfSUQnLCAnUkYnKV0sIGJ5ID0gJ0FHX21ldGhvZG9sb2d5X0lEJykgJT4lDQogIHJlbmFtZShSRl9BTlBQID0gUkYpICU+JQ0KICBsZWZ0X2pvaW4oQkdfbWV0aG9kb2xvZ3lbLCBjKCdCR19tZXRob2RvbG9neV9JRCcsICdSRicpXSwgYnkgPSAnQkdfbWV0aG9kb2xvZ3lfSUQnKSAlPiUNCiAgcmVuYW1lKFJGX0JOUFAgPSBSRikgJT4lDQogIGxlZnRfam9pbihzaXRlX2luZm9bLGMoJ3NpdGVfSUQnLCAnYmlvbWVfY29kZScpXSwgYnkgPSAnc2l0ZV9JRCcpICU+JQ0KICBkaXN0aW5jdCgpICU+JQ0KICByZW5hbWUoYmlvbWUgPSBiaW9tZV9jb2RlKQ0KDQpjbGltYXRlX2RhdGEgPC0gcmVhZF9leGNlbChwYXN0ZShEQl9maWxlX3BhdGgsICdCaW9tYXNzUHJvZHVjdGlvbl9EQl9WNi4xLjFfY29weS54bHN4Jywgc2VwID0iIiksIHNoZWV0ID0gMTIpICU+JQ0KICBkcGx5cjo6c2VsZWN0KGMoJ3NpdGVfSUQnLCAnYmlvMScsICdiaW8xMicpKSAlPiUNCiAgZGlzdGluY3QoKQ0KYGBgDQoNCmBgYHtyfQ0KDQojLS0tLSBEZWZpbmUgdGhlIGNvbG9yIHNjaGVtZQ0KY29sb3Jfc2NoZW1lIDwtICAgc2NhbGVfY29sb3JfbWFudWFsKHZhbHVlcyA9IGMoIiNkYjdjMjYiLCAiIzAwODAwMCIsICIjYTdjOTU3IiwiIzUzOTBkOSIgLCIjZmRjNTAwIiwgJyNiZWU5ZTgnKSkNCmBgYA0KDQojIyBXaGl0dGFrZXItZGlhZ3JhbQ0KDQpgYGB7cn0NCldoaXR0YWtlcl9wbG90X2RhdGEgPC0gc2l0ZV9pbmZvWywgYygnc2l0ZV9JRCcsICdiaW9tZV9jb2RlJywgJ2NsaW1hdGVfcmVnaW9uJywgJ21hbmFnZW1lbnRfY29kZScpXSAlPiUNCiAgbGVmdF9qb2luKGNsaW1hdGVfZGF0YVssIGMoJ3NpdGVfSUQnLCAnYmlvMScsICdiaW8xMicpXSwgYnkgPSAnc2l0ZV9JRCcpJT4lDQogIHJlbmFtZSh0ZW1wZXJhdHVyZSA9IGJpbzEsIHByZWNpcGl0YXRpb24gPSBiaW8xMikgJT4lDQogIHJvd3dpc2UoKSAlPiUNCiAgbXV0YXRlKHByZWNpcGl0YXRpb24gPSAgcHJlY2lwaXRhdGlvbi8xMCkgJT4lDQogIG11dGF0ZShiaW9tZV9jb2RlID0gaWZlbHNlKGJpb21lX2NvZGUgPT0gJ0cnLCAnZ3Jhc3NsYW5kJywgDQogICAgICAgICAgICAgICAgICAgICAgICAgICAgIGlmZWxzZShiaW9tZV9jb2RlID09J0YnLCAnZm9yZXN0JywgDQogICAgICAgICAgICAgICAgICAgICAgICAgICAgICAgICAgICBpZmVsc2UoYmlvbWVfY29kZSA9PSdDJywgJ2Nyb3BsYW5kJywNCiAgICAgICAgICAgICAgICAgICAgICAgICAgICAgICAgICAgICAgICAgICBpZmVsc2UoYmlvbWVfY29kZSA9PSAnU2gnLCAnZHJ5IHNocnVibGFuZCcsIA0KICAgICAgICAgICAgICAgICAgICAgICAgICAgICAgICAgICAgICAgICAgICAgICAgICBpZmVsc2UoYmlvbWVfY29kZSA9PSdUJywgJ3R1bmRyYScsDQogICAgICAgICAgICAgICAgICAgICAgICAgICAgICAgICAgICAgICAgICAgICAgICAgICAgICAgICBpZmVsc2UoYmlvbWVfY29kZSA9PSdQJywgJ25vcnRoZXJuIHBlYXRsYW5kJywgTkEpKSkpKSkpICU+JQ0KICBtdXRhdGUoRWNvc3lzdGVtX3R5cGUgPSBwYXN0ZShjbGltYXRlX3JlZ2lvbiwgYmlvbWVfY29kZSwgc2VwID0gIiAiKSkgJT4lDQogIG11dGF0ZShFY29zeXN0ZW1fdHlwZSA9IHJlcGxhY2UoRWNvc3lzdGVtX3R5cGUsICBiaW9tZV9jb2RlID09ICJub3J0aGVybiBwZWF0bGFuZCIsICJub3J0aGVybiBwZWF0bGFuZCIpKSAlPiUNCiAgbXV0YXRlKEVjb3N5c3RlbV90eXBlID0gcmVwbGFjZShFY29zeXN0ZW1fdHlwZSwgIGJpb21lX2NvZGUgPT0gImNyb3BsYW5kIiwgImNyb3BsYW5kIikpJT4lDQogIG11dGF0ZShFY29zeXN0ZW1fdHlwZSA9IHJlcGxhY2UoRWNvc3lzdGVtX3R5cGUsICBiaW9tZV9jb2RlID09ICJkcnkgc2hydWJsYW5kIiwgImRyeSBzaHJ1YmxhbmQiKSklPiUNCiAgbXV0YXRlKEVjb3N5c3RlbV90eXBlID0gcmVwbGFjZShFY29zeXN0ZW1fdHlwZSwgIGJpb21lX2NvZGUgPT0gInR1bmRyYSIsICJ0dW5kcmEiKSkgJT4lDQogIGZpbHRlcighaXMubmEoY2xpbWF0ZV9yZWdpb24pKSAlPiUNCiAgcmVuYW1lKGBCaW9tZWAgPSAnYmlvbWVfY29kZScpIA0KDQpteV9vdXRsaWVycyA8LSBnZXRfb3V0bGllcnModHAgPSBXaGl0dGFrZXJfcGxvdF9kYXRhWywgNTo2XSkgDQoNCm15X291dGxpZXJzX3NpdGVfSUQgPC0gYygnQ0EtdHJ1LUQwMScsDQogICAgICAgICAgICAgICAgICAgICAgICAgJ0lOLW5hMi1EJywNCiAgICAgICAgICAgICAgICAgICAgICAgICAnSU4tbmEzLUQnLA0KICAgICAgICAgICAgICAgICAgICAgICAgICdJTi1uYTMtRCcsDQogICAgICAgICAgICAgICAgICAgICAgICAgJ0lOLXJsdS1EMDEnLA0KICAgICAgICAgICAgICAgICAgICAgICAgICdJTi11c2gtRDAxJywNCiAgICAgICAgICAgICAgICAgICAgICAgICAnVVMtY2FzLUQwMScsDQogICAgICAgICAgICAgICAgICAgICAgICAgJ1VTLWNhcy1EMDInLA0KICAgICAgICAgICAgICAgICAgICAgICAgICdVUy1jYXMtRDAzJywNCiAgICAgICAgICAgICAgICAgICAgICAgICAnVVMtY2FzLUQwNCcNCikgIA0KDQpXaGl0dGFrZXJfcGxvdF9kYXRhIDwtIFdoaXR0YWtlcl9wbG90X2RhdGEgJT4lDQogIG11dGF0ZShzdGF0dXMgPSBpZmVsc2Uoc2l0ZV9JRCAlaW4lIG15X291dGxpZXJzX3NpdGVfSUQsICcjOWIyMjI2JywgJyM0YTRlNjknKSkNCg0KDQpteV9wYWxldHRlIDwtIGNvbG9yUmFtcFBhbGV0dGUoY29sb3JzID0gYygiIzYyYjZjYiIsICAiIzZhOTk0ZSIsICIjZmZiYTA4IikpKDkpDQpgYGANCg0KYGBge3IsIHdhcm5pbmc9RkFMU0UsIGZpZy53aWR0aD0xMSxmaWcuaGVpZ2h0PTd9DQp3aGl0dGFrZXJfYmFzZV9wbG90KGNvbG9yX3BhbGV0dGUgPSBteV9wYWxldHRlKSArDQogICMgYWRkIHRoZSB0ZW1wZXJhdHVyZSAtIHByZWNpcGl0YXRpb24gZGF0YSBwb2ludHMNCiAgZ2VvbV9wb2ludChkYXRhID0gV2hpdHRha2VyX3Bsb3RfZGF0YVtXaGl0dGFrZXJfcGxvdF9kYXRhJEJpb21lICE9ICdjcm9wbGFuZCcsXSwgDQogICAgICAgICAgICAgYWVzKHggPSB0ZW1wZXJhdHVyZSwgDQogICAgICAgICAgICAgICAgIHkgPSBwcmVjaXBpdGF0aW9uLA0KICAgICAgICAgICAgICAgICBzaGFwZSA9IGBCaW9tZWApLCANCiAgICAgICAgICAgICBzaXplICA9IDMsDQogICAgICAgICAgICAgI3NoYXBlID0gMjEsDQogICAgICAgICAgICAgY29sb3VyID0gV2hpdHRha2VyX3Bsb3RfZGF0YVtXaGl0dGFrZXJfcGxvdF9kYXRhJEJpb21lICE9ICdjcm9wbGFuZCcsXSRzdGF0dXMsDQogICAgICAgICAgICAgc3Ryb2tlID0gMS41LA0KICAgICAgICAgICAgIGFscGhhID0gMC43NSkgKyB4bGFiKCJUZW1wZXJhdHVyZSBbXSIpICsgeWxhYigiUHJlY2lwaXRhdGlvbiBbY21dIikgICsNCiAgdGhlbWVfYncoKSAgKw0KICB0aGVtZSgNCiAgICBsZWdlbmQuanVzdGlmaWNhdGlvbiA9IGMoMCwgMSksICMgcGljayB0aGUgdXBwZXIgbGVmdCBjb3JuZXIgb2YgdGhlIGxlZ2VuZCBib3ggYW5kDQogICAgbGVnZW5kLnBvc2l0aW9uID0gYygwLCAxKSwgIyBhZGp1c3QgdGhlIHBvc2l0aW9uIG9mIHRoZSBjb3JuZXIgYXMgcmVsYXRpdmUgdG8gYXhpcw0KICAgIGxlZ2VuZC5iYWNrZ3JvdW5kID0gZWxlbWVudF9yZWN0KGZpbGwgPSBOQSksICMgdHJhbnNwYXJlbnQgbGVnZW5kIGJhY2tncm91bmQNCiAgICBsZWdlbmQuYm94ID0gImhvcml6b250YWwiLCAjIGhvcml6b250YWwgYXJyYW5nZW1lbnQgb2YgbXVsdGlwbGUgbGVnZW5kcw0KICAgIGxlZ2VuZC5zcGFjaW5nLnggPSB1bml0KDAuNSwgdW5pdHMgPSAiY20iKSwgIyBob3Jpem9udGFsIHNwYWNpbmcgYmV0d2VlbiBsZWdlbmRzDQogICAgcGFuZWwuZ3JpZCA9IGVsZW1lbnRfYmxhbmsoKSwgIyBlbGltaW5hdGUgZ3JpZHMNCiAgICB0ZXh0PWVsZW1lbnRfdGV4dChzaXplPTE4KQ0KICApDQpgYGANCiMjIFNpdGUgbG9jYXRpb24gKGdlb2dyYXBoaWNhbCkgcGxvdA0KDQpgYGB7cn0NCnNpdGVfbG9jYXRpb24gPC0gc2l0ZV9pbmZvWywgYygnc2l0ZV9JRCcsICdsYXRpdHVkZScsICdub3J0aF9zb3V0aCcsICdsb25naXR1ZGUnLCAnZWFzdF93ZXN0JywgJ2NsaW1hdGVfcmVnaW9uJywgJ2Jpb21lX2NvZGUnLCAnZWxldmF0aW9uJywgJ21hbmFnZW1lbnRfY29kZScpXSAlPiUNCiAgcm93d2lzZSgpICU+JQ0KICBtdXRhdGUobWFuYWdlbWVudF9jb2RlID0gcmVwbGFjZShtYW5hZ2VtZW50X2NvZGUsIG1hbmFnZW1lbnRfY29kZSA9PSAnTScsICdtYW5hZ2VkJykpICU+JQ0KICBtdXRhdGUobWFuYWdlbWVudF9jb2RlID0gcmVwbGFjZShtYW5hZ2VtZW50X2NvZGUsIG1hbmFnZW1lbnRfY29kZSA9PSAnTicsICd1bm1hbmFnZWQnKSkgJT4lDQogIG11dGF0ZShsYXRpdHVkZSA9IHJlcGxhY2UobGF0aXR1ZGUsIG5vcnRoX3NvdXRoID09ICdTJywgLWxhdGl0dWRlKSkgJT4lDQogIG11dGF0ZShsb25naXR1ZGUgPSByZXBsYWNlKGxvbmdpdHVkZSwgZWFzdF93ZXN0ID09ICdXJywgLWxvbmdpdHVkZSkpICU+JQ0KICByZW5hbWUoQmlvbWUgPSBiaW9tZV9jb2RlLCBDbGltYXRlID0gY2xpbWF0ZV9yZWdpb24pIA0KDQp3b3JsZF9tYXAgPC0gZ2dwbG90KG1hcHM6Om1hcCgnd29ybGQnLGludGVyaW9yPUZBTFNFLGNvbD0nZ3JheScsIGZpbGw9VFJVRSxsdHk9MCksIGFlcyh4ID0gbG9uZywgeSA9IGxhdCwgZ3JvdXAgPSBncm91cCkpICsNCiAgZ2VvbV9wb2x5Z29uKGZpbGw9J2RhcmtncmF5JywgY29sb3VyID0gImRhcmtncmF5IikgKyB4bGFiKCJMb25naXR1ZGUiKSArIHlsYWIoIkxhdGl0dWRlIikgKw0KICBnZW9tX3BvaW50KGRhdGEgPSBzaXRlX2xvY2F0aW9uLCBtYXBwaW5nID0gYWVzKHggPSBsb25naXR1ZGUsIHkgPSBsYXRpdHVkZSwgZmlsbCA9IEJpb21lKSwgY29sb3VyPSJibGFjayIscGNoPTIxLCBpbmhlcml0LmFlcyA9IEZBTFNFLCBzaXplID0gMywgKSAgKw0KICB0aGVtZV9idygpICsNCiAgc2NhbGVfZmlsbF9tYW51YWwodmFsdWVzID0gYygiI2RiN2MyNiIsICIjMDA4MDAwIiwgIiNhN2M5NTciLCIjNTM5MGQ5IiAsIiNmZGM1MDAiLCAnI2JlZTllOCcpKSArIHRoZW1lKHRleHQ9ZWxlbWVudF90ZXh0KHNpemU9MTUpKQ0KYGBgDQoNCmBgYHtyfQ0KcGxvdCh3b3JsZF9tYXApDQpgYGANCg0KIyMgTWVhc3VyZW1lbnQgeWVhciBkaXN0cmlidXRpb24gcGxvdA0KDQpgYGB7ciwgZmlnLndpZHRoPTExLGZpZy5oZWlnaHQ9NX0NCm1lYXN1cmVtZW50X3llYXIgPC0gTlBQX2VzdGltYXRlcyAlPiUNCiAgZHBseXI6OnNlbGVjdChzaXRlX0lELCBiZWdpbl95ZWFyLCBlbmRfeWVhcikgJT4lDQogIHJvd3dpc2UoKSU+JQ0KICBtdXRhdGUoYmVnaW5feWVhciA9IHJlcGxhY2UoYmVnaW5feWVhciwgYmVnaW5feWVhciA9PSA5OTk5LCBlbmRfeWVhcikpICU+JSANCiAgbXV0YXRlKHllYXIgPSBtYXAyKGJlZ2luX3llYXIsIGVuZF95ZWFyLCB+c2VxKGZyb20gPSAueCwgdG8gPSAueSwgYnkgPSAxKSkpICU+JQ0KICB1bm5lc3QoKSAlPiUNCiAgZHBseXI6OnNlbGVjdChjKCdzaXRlX0lEJywgJ3llYXInKSkgJT4lDQogIGxlZnRfam9pbihzaXRlX2luZm9bLCBjKCdzaXRlX0lEJywgJ2Jpb21lX2NvZGUnLCAnY2xpbWF0ZV9yZWdpb24nKV0pICU+JQ0KICBkaXN0aW5jdCgpICU+JQ0KICByZW5hbWUoQmlvbWUgPSBiaW9tZV9jb2RlKQ0KDQplbmRfbWVhc3VyZW1lbnRfeWVhciA8LSBOUFBfZXN0aW1hdGVzICU+JQ0KICBkcGx5cjo6c2VsZWN0KHNpdGVfSUQsIGVuZF95ZWFyKSAlPiUNCiAgbGVmdF9qb2luKHNpdGVfaW5mb1ssIGMoJ3NpdGVfSUQnLCAnYmlvbWVfY29kZScsICdjbGltYXRlX3JlZ2lvbicpXSkgJT4lDQogIGRpc3RpbmN0KCkgJT4lDQogIHJlbmFtZShCaW9tZSA9IGJpb21lX2NvZGUpIA0KDQpnZ3Bsb3QoZW5kX21lYXN1cmVtZW50X3llYXIsIGFlcyh4ID0gZW5kX3llYXIsIGZpbGwgPSBCaW9tZSkpICsgDQogIGFubm90YXRlKCdyZWN0JywgeG1pbj0xOTY0LCB4bWF4PTE5NzQsIHltaW49MCwgeW1heD1JbmYsIGFscGhhPS41LCBmaWxsPSdkYXJrZ3JheScpKw0KICBhbm5vdGF0ZSgidGV4dCIsIHg9MTk2OS4yLCB5PTQwLCBsYWJlbD0gIklCUCIsc2l6ZSA9IHVuaXQoNiwgInB0IikpICsgZ2VvbV9oaXN0b2dyYW0oYmlucyA9IDY0LCBjb2xvciA9ICdibGFjaycpICsgDQogIGxhYnMoeSA9ICdEYXRhYmFzZSByZWNvcmQnLCB4ID0gJ0VuZCB5ZWFyIG9mIG1lYXN1cmVtZW50cycpKw0KICB0aGVtZV9idygpICsgIHNjYWxlX2ZpbGxfbWFudWFsKHZhbHVlcyA9IGMoIiNkYjdjMjYiLCAiIzAwODAwMCIsICIjYTdjOTU3IiwiIzUzOTBkOSIgLCIjZmRjNTAwIiwgJyNiZWU5ZTgnKSkgKyB0aGVtZSh0ZXh0PWVsZW1lbnRfdGV4dChzaXplPTE1KSkgKyBnZW9tX3NlZ21lbnQoYWVzKHggPSAxOTY2LjcsIHkgPSA0MCwgeGVuZCA9IDE5NjQsIHllbmQgPSA0MCksDQogICAgICAgICAgICAgICAgICBhcnJvdyA9IGFycm93KGxlbmd0aCA9IHVuaXQoMC4yLCAiY20iKSksIHNpemUgPSAxKSsgdGhlbWUodGV4dD1lbGVtZW50X3RleHQoc2l6ZT0xNSkpICsgZ2VvbV9zZWdtZW50KGFlcyh4ID0gMTk3MS43LCB5ID0gNDAsIHhlbmQgPSAxOTc0LCB5ZW5kID0gNDApLA0KICAgICAgICAgICAgICAgICAgYXJyb3cgPSBhcnJvdyhsZW5ndGggPSB1bml0KDAuMiwgImNtIikpLCBzaXplID0gMSkNCmBgYA0KDQpgYGB7ciwgd2FybmluZyA9IEZBTFNFLCBmaWcud2lkdGg9MTEsZmlnLmhlaWdodD01fQ0KZ2dwbG90KHNpdGVfaW5mbywgYWVzKHg9ZWxldmF0aW9uLCBmaWxsID0gYmlvbWVfY29kZSkpKyBnZW9tX2hpc3RvZ3JhbShiaW5zID0gNTAsIGNvbG9yID0gJ2JsYWNrJykgKyANCiAgbGFicyh5ID0gJ0RhdGFiYXNlIHJlY29yZCcsIHggPSAnRWxldmF0aW9uIFttXScsIGZpbGwgPSAnQmlvbWUnKSArDQogIHRoZW1lX2J3KCkgKyAgc2NhbGVfZmlsbF9tYW51YWwodmFsdWVzID0gYygiI2RiN2MyNiIsICIjMDA4MDAwIiwgIiNhN2M5NTciLCIjNTM5MGQ5IiAsIiNmZGM1MDAiLCAnI2JlZTllOCcpKSArIHRoZW1lKHRleHQ9ZWxlbWVudF90ZXh0KHNpemU9MTUpKQ0KYGBgDQoNCiMjIEJveHBsb3RzIG9mIE5QUCB2cy5iaW9tZSBhbmQgY2xpbWF0ZQ0KDQojIyMgQ29tcHV0ZSBzaXRlIGF2ZXJhZ2VkIEFOUFAsIEJOUFAgYW5kIFROUFAgYW5kIHVuY2VydGFpbnR5DQoNCg0KDQpgYGB7cn0NCiMgRGVmaW5lIFBCaWsgZm9yIGVhY2ggYmlvbWUgYW5kIGNvbXBvbmVudA0KDQpQQl9DX0FHIDwtIDgzMA0KUEJfQ19CRyA8LSAxODANClBCX0dfQUcgPC0gNTUwDQpQQl9HX0JHIDwtIDYwMA0KUEJfRl9BRyA8LSA5MzANClBCX0ZfQkcgPC0gMzgwDQpQQl9TaF9BRyA8LSAxMjANClBCX1NoX0JHIDwtIDMwMA0KUEJfUF9BRyA8LSAyNDANClBCX1BfQkcgPC0gMzcwDQpQQl9UX0FHIDwtIDE2MA0KUEJfVF9CRyA8LSAxMTANCg0KUEJfQ19UTlBQIDwtIDEwMDANClBCX0dfVE5QUCA8LSA5NzANClBCX0ZfVE5QUCA8LSAxMTcwDQpQQl9TaF9UTlBQIDwtIDM4MA0KUEJfUF9UTlBQIDwtIDUwMA0KUEJfVF9UTlBQIDwtIDM2MA0KDQoNCiNBTlBQIC0tLS0NCiMjIENyb3BsYW5kIGFuZCBHcmFzc2xhbmQNCkFOUFBfY3JvcF9ncmFzcyA8LSBOUFBfZXN0aW1hdGVzICU+JQ0KICBmaWx0ZXIoYmlvbWUgJWluJSBjKCdHJywgJ0MnKSkgJT4lDQogIGRwbHlyOjpzZWxlY3QoJ2Jpb21lJywgJ3NpdGVfSUQnLCAnYmVnaW5feWVhcicsICdlbmRfeWVhcicsICdoZXJicycsICdzaHJ1YnMnLCANCiAgICAgICAgICAgICAgICAnQU5QUF9oZXJic19zaHJ1YnMnLCdBTlBQX25vbl90cmVlJyAsJ0FOUFBfdHJlZScsICdoZXJiaXZvcnknLCAncmVwcm9kdWN0aXZlX21hdGVyaWFsJywNCiAgICAgICAgICAgICAgICAnUkZfQU5QUCcpICU+JQ0KICBtdXRhdGUodHNfbGVuZ3RoID0gMSArIChlbmRfeWVhciAtIGJlZ2luX3llYXIpKSAlPiUNCiAgbXV0YXRlKHRzX2xlbmd0aCA9IHJlcGxhY2UodHNfbGVuZ3RoLCBiZWdpbl95ZWFyID09IDk5OTksIDEpKSAlPiUNCiAgcm93d2lzZSgpICU+JQ0KICBtdXRhdGUoQU5QUF9ub25fdHJlZSA9IHJlcGxhY2UoQU5QUF9ub25fdHJlZSxpcy5uYShBTlBQX25vbl90cmVlKSAmICEoaXMubmEoc2hydWJzKSAmIGlzLm5hKGhlcmJzKSksIHN1bShoZXJicywgc2hydWJzLCBuYS5ybSA9IFQpKSkgJT4lDQogIG11dGF0ZShBTlBQID0gaWZlbHNlKCEoaXMubmEoQU5QUF9ub25fdHJlZSkgJiBpcy5uYShBTlBQX3RyZWUpKSwgc3VtKEFOUFBfbm9uX3RyZWUsIEFOUFBfdHJlZSwgaGVyYml2b3J5LCBuYS5ybSA9IFQpLCBOQSkpICU+JQ0KICBtdXRhdGUodW5jZXJ0YWludHlfQUcgPSBpZmVsc2UoYmlvbWUgPT0gJ0MnLCBQQl9DX0FHKlJGX0FOUFAvc3FydCh0c19sZW5ndGgpLA0KICAgICAgICAgICAgICAgICAgICAgICAgICAgICAgICAgaWZlbHNlKGJpb21lID09ICdHJywgUEJfR19BRypSRl9BTlBQL3NxcnQodHNfbGVuZ3RoKSwgTkEpKSkgJT4lDQogIGZpbHRlcighaXMubmEoQU5QUCkpICU+JQ0KICBkcGx5cjo6bGVmdF9qb2luKGRhdGEuZnJhbWUodGFibGUoTlBQX2VzdGltYXRlcyA9IC5bLGMoJ3NpdGVfSUQnKV0pKSkgJT4lDQogIHJlbmFtZShBTlBQX2NvdW50ID0gRnJlcSkgJT4lDQogIGdyb3VwX2J5KHNpdGVfSUQpICU+JQ0KICByZWZyYW1lKEFOUFAgPSBtZWFuKEFOUFAsIG5hLnJtID0gVCksIA0KICAgICAgICAgIHVuY2VydGFpbnR5X0FHID0gc3FydChzdW0oKHVuY2VydGFpbnR5X0FHKV4yLCBuYS5ybSA9IFQpKS9BTlBQX2NvdW50KSAlPiUNCiAgZGlzdGluY3QoKQ0KDQoNCg0KIyMgUGVhdGxhbmQgYW5kIFR1bmRyYQ0KQU5QUF9wZWF0X3R1bmQgPC0gTlBQX2VzdGltYXRlcyAlPiUNCiAgZmlsdGVyKGJpb21lICVpbiUgYygnUCcsICdUJykpICU+JQ0KICBkcGx5cjo6c2VsZWN0KCdiaW9tZScsICdzaXRlX0lEJywgJ2JlZ2luX3llYXInLCAnZW5kX3llYXInLCAnQU5QUF9oZXJic19zaHJ1YnMnLCAnbm9uX3Zhc2N1bGFyJyAsJ0FOUFBfbm9uX3RyZWUnICwnQU5QUF90cmVlJywNCiAgICAgICAgICAgICAgICAnUkZfQU5QUCcpICU+JQ0KICBmaWx0ZXIoIShpcy5uYShBTlBQX2hlcmJzX3NocnVicykgJiBpcy5uYShub25fdmFzY3VsYXIpICYgaXMubmEoQU5QUF9ub25fdHJlZSkgJiBpcy5uYShBTlBQX3RyZWUpKSkgJT4lDQogIGZpbHRlcighaXMubmEoUkZfQU5QUCkpICU+JQ0KICBkcGx5cjo6bGVmdF9qb2luKGRhdGEuZnJhbWUodGFibGUoTlBQX2VzdGltYXRlcyA9IC5bLGMoJ3NpdGVfSUQnKV0pKSkgJT4lDQogIHJlbmFtZShBTlBQX2NvdW50ID0gRnJlcSkgJT4lDQogIG11dGF0ZSh0c19sZW5ndGggPSAxICsgKGVuZF95ZWFyIC0gYmVnaW5feWVhcikpICU+JQ0KICBtdXRhdGUodHNfbGVuZ3RoID0gcmVwbGFjZSh0c19sZW5ndGgsIGJlZ2luX3llYXIgPT0gOTk5OSwgMSkpICU+JQ0KICByb3d3aXNlKCkgJT4lDQogIG11dGF0ZShBTlBQX25vbl90cmVlID0gcmVwbGFjZShBTlBQX25vbl90cmVlLCBpcy5uYShBTlBQX25vbl90cmVlKSAmICEoaXMubmEoQU5QUF9oZXJic19zaHJ1YnMpIHwgaXMubmEobm9uX3Zhc2N1bGFyKSksIHN1bShBTlBQX2hlcmJzX3NocnVicywgbm9uX3Zhc2N1bGFyLCBuYS5ybSA9IFQpKSkgJT4lDQogIG11dGF0ZShBTlBQX25vbl90cmVlID0gcmVwbGFjZShBTlBQX25vbl90cmVlLCBpcy5uYShBTlBQX25vbl90cmVlKSAmIHNpdGVfSUQgJWluJSBjKCdTRS1rYXItRDAxX0YnLCAnU0Uta2FyLUQwMV9DJyksDQogICAgICAgICAgICAgICAgICAgICAgICAgICAgICAgICBzdW0oQU5QUF9oZXJic19zaHJ1YnMsIG5vbl92YXNjdWxhciwgbmEucm0gPSBUKSkpICU+JQ0KICBtdXRhdGUoQU5QUCA9IGlmZWxzZSghaXMubmEoQU5QUF9ub25fdHJlZSksIHN1bShBTlBQX25vbl90cmVlLCBBTlBQX3RyZWUsIG5hLnJtID0gVCksIA0KICAgICAgICAgICAgICAgICAgICAgICBpZmVsc2UoaXMubmEoQU5QUF9ub25fdHJlZSkgJiBBTlBQX2NvdW50ID09IDEgLCBzdW0oQU5QUF9oZXJic19zaHJ1YnMsIG5vbl92YXNjdWxhciwgbmEucm0gPSBUKSwgTkEpKSkgJT4lDQogIG11dGF0ZSh1bmNlcnRhaW50eV9BRyA9IGlmZWxzZShiaW9tZSA9PSAnUCcsIFBCX1BfQUcqUkZfQU5QUC9zcXJ0KHRzX2xlbmd0aCksDQogICAgICAgICAgICAgICAgICAgICAgICAgICAgICAgICBpZmVsc2UoYmlvbWUgPT0gJ1QnLCBQQl9UX0FHKlJGX0FOUFAvc3FydCh0c19sZW5ndGgpLCBOQSkpKSAlPiUNCiAgbXV0YXRlKHVuY2VydGFpbnR5X2hlcmJzX3NocnVicyA9ICBpZmVsc2UoaXMubmEoQU5QUCkgJg0KICAgICAgICAgICAgICAgICAgICAgICAgICAgICAgICAgICAgICAgICAgICAgICFpcy5uYShBTlBQX2hlcmJzX3NocnVicyksIHVuY2VydGFpbnR5X0FHICwgTkEpKSAlPiUNCiAgZ3JvdXBfYnkoc2l0ZV9JRCkgJT4lDQogIHJlZnJhbWUoQU5QUCA9IG1lYW4oQU5QUCwgbmEucm0gPSBUKSwgDQogICAgICAgICAgQU5QUF9ub25fdmFzY3VsYXIgPSBtZWFuKG5vbl92YXNjdWxhciwgbmEucm0gPSBUKSwgDQogICAgICAgICAgQU5QUF9oZXJic19zaHJ1YnMgPSBtZWFuKEFOUFBfaGVyYnNfc2hydWJzLCBuYS5ybSA9IFQpLCANCiAgICAgICAgICB1bmNlcnRhaW50eV9BRyA9IHNxcnQoc3VtKCh1bmNlcnRhaW50eV9BRyleMiwgbmEucm0gPSBUKSkvQU5QUF9jb3VudCwNCiAgICAgICAgICB1bmNlcnRhaW50eV9oZXJic19zaHJ1YnMgPSBzcXJ0KHN1bSgodW5jZXJ0YWludHlfaGVyYnNfc2hydWJzKV4yLCBuYS5ybSA9IFQpKSkgJT4lDQogIGRpc3RpbmN0KCkgJT4lDQogIG11dGF0ZV9pZihpcy5udW1lcmljLCBsaXN0KH5uYV9pZiguLCAwLjAwMDApKSklPiUNCiAgbXV0YXRlX2lmKGlzLm51bWVyaWMsIGxpc3Qofm5hX2lmKC4sIE5hTikpKSAlPiUNCiAgcm93d2lzZSgpICU+JQ0KICBtdXRhdGUodW5jZXJ0YWludHlfQUcgPSByZXBsYWNlKHVuY2VydGFpbnR5X0FHLCBpcy5uYShBTlBQKSwgdW5jZXJ0YWludHlfaGVyYnNfc2hydWJzKSkgJT4lDQogIG11dGF0ZShBTlBQID0gcmVwbGFjZShBTlBQLCBpcy5uYShBTlBQKSwgc3VtKEFOUFBfbm9uX3Zhc2N1bGFyLCBBTlBQX2hlcmJzX3NocnVicywgbmEucm0gPSBUKSkpICU+JQ0KICBkaXN0aW5jdCgpDQoNCiMjIEZvcmVzdCBhbmQgU2hydWJsYW5kIA0KQU5QUF9mb3JlX3NocnViIDwtIE5QUF9lc3RpbWF0ZXMgJT4lDQogIGZpbHRlcihiaW9tZSAlaW4lIGMoJ0YnLCAnU2gnKSkgJT4lDQogIGRwbHlyOjpzZWxlY3QoJ2Jpb21lJywgJ3NpdGVfSUQnLCAnYmVnaW5feWVhcicsICdlbmRfeWVhcicsICdBTlBQX25vbl90cmVlJywgJ3N0ZW0nLCAnYnJhbmNoJywgDQogICAgICAgICAgICAgICAgJ0FOUFBfd29vZCcsICdBTlBQX3RvdF9saXR0ZXInLCAnQU5QUF90cmVlJywgJ2hlcmJpdm9yeScsICdyZXByb2R1Y3RpdmVfbWF0ZXJpYWwnLA0KICAgICAgICAgICAgICAgICdSRl9BTlBQJykgJT4lDQogIGZpbHRlcighKGlzLm5hKEFOUFBfbm9uX3RyZWUpICYgaXMubmEoc3RlbSkgJiBpcy5uYShBTlBQX3dvb2QpICYgaXMubmEoQU5QUF90b3RfbGl0dGVyKSAmIA0KICAgICAgICAgICAgIGlzLm5hKEFOUFBfdHJlZSkgJiBpcy5uYShoZXJiaXZvcnkpICYgaXMubmEocmVwcm9kdWN0aXZlX21hdGVyaWFsKSkpICU+JQ0KICBkcGx5cjo6bGVmdF9qb2luKGRhdGEuZnJhbWUodGFibGUoTlBQX2VzdGltYXRlcyA9IC5bLGMoJ3NpdGVfSUQnKV0pKSkgJT4lDQogIHJlbmFtZShBTlBQX2NvdW50ID0gRnJlcSkgJT4lDQogIG11dGF0ZShBTlBQX2NvdW50ID0gcmVwbGFjZShBTlBQX2NvdW50LCBzaXRlX0lEID09ICdKUC1UYTEtRDAxJywgNykpICU+JQ0KICBtdXRhdGUoQU5QUF9jb3VudCA9IHJlcGxhY2UoQU5QUF9jb3VudCwgc2l0ZV9JRCA9PSAnVVMtRGszLUYwMScsIDEpKSAlPiUNCiAgcm93d2lzZSgpICU+JQ0KICBtdXRhdGUodHNfbGVuZ3RoID0gMSArIChlbmRfeWVhciAtIGJlZ2luX3llYXIpKSAlPiUNCiAgbXV0YXRlKHRzX2xlbmd0aCA9IHJlcGxhY2UodHNfbGVuZ3RoLCBiZWdpbl95ZWFyID09IDk5OTksIDEpKSAlPiUNCiAgbXV0YXRlKEFOUFBfd29vZCA9IHJlcGxhY2UoQU5QUF93b29kLCBpcy5uYShBTlBQX3dvb2QpICYgIWlzLm5hKHN0ZW0pLCBzdW0oc3RlbSwgYnJhbmNoLCBuYS5ybSA9IFQpKSkgJT4lDQogIG11dGF0ZShBTlBQX3RyZWUgPSByZXBsYWNlKEFOUFBfdHJlZSwgaXMubmEoQU5QUF90cmVlKSAmICFpcy5uYShBTlBQX3dvb2QpLCBzdW0oQU5QUF93b29kLCBBTlBQX3RvdF9saXR0ZXIsIG5hLnJtID0gVCkpKSAlPiUNCiAgbXV0YXRlKHVuY2VydGFpbnR5X0FHID0gaWZlbHNlKGJpb21lID09ICdGJywgUEJfRl9BRypSRl9BTlBQL3NxcnQodHNfbGVuZ3RoKSwNCiAgICAgICAgICAgICAgICAgICAgICAgICAgICAgICAgIGlmZWxzZShiaW9tZSA9PSAnUycsIFBCX1NoX0FHKlJGX0FOUFAvc3FydCh0c19sZW5ndGgpLCBOQSkpKSAlPiUNCiAgZ3JvdXBfYnkoc2l0ZV9JRCkgJT4lDQogIHJlZnJhbWUoQU5QUF91bmRlcnN0b3J5ID0gbWVhbihBTlBQX25vbl90cmVlLCBuYS5ybSA9IFQpLA0KICAgICAgICAgIEFOUFBfdHJlZSA9IG1lYW4oQU5QUF90cmVlLCBuYS5ybSA9IFQpLCANCiAgICAgICAgICBBTlBQX3JlcHJvZHVjdGl2ZV9tYXRlcmlhbCA9IG1lYW4ocmVwcm9kdWN0aXZlX21hdGVyaWFsLCBuYS5ybSA9IFQpLCANCiAgICAgICAgICBBTlBQX2hlcmJpdm9yeSA9IG1lYW4oaGVyYml2b3J5LCBuYS5ybSA9IFQpLCANCiAgICAgICAgICB1bmNlcnRhaW50eV9BRyA9IHNxcnQoc3VtKCh1bmNlcnRhaW50eV9BRyleMiwgbmEucm0gPSBUKSkvQU5QUF9jb3VudCkgJT4lDQogIGRpc3RpbmN0KCkgJT4lDQogIG11dGF0ZV9pZihpcy5udW1lcmljLCBsaXN0KH5uYV9pZiguLCBOYU4pKSkgJT4lDQogIHJvd3dpc2UoKSAlPiUNCiAgbXV0YXRlKEFOUFAgPSBzdW0oQU5QUF91bmRlcnN0b3J5LCBBTlBQX3RyZWUsIEFOUFBfcmVwcm9kdWN0aXZlX21hdGVyaWFsLCANCiAgICAgICAgICAgICAgICAgICAgQU5QUF9oZXJiaXZvcnksIG5hLnJtID0gVCkpICAlPiUNCiAgZGlzdGluY3QoKQ0KDQoNCg0KI0JOUFAgLS0tLQ0KIyMgQ3JvcGxhbmQgYW5kIEdyYXNzbGFuZA0KQk5QUF9jcm9wX2dyYXNzIDwtIE5QUF9lc3RpbWF0ZXMgJT4lDQogIGZpbHRlcihiaW9tZSAlaW4lIGMoJ0cnLCAnQycpKSAlPiUNCiAgZHBseXI6OnNlbGVjdCgnYmlvbWUnLCAnc2l0ZV9JRCcsICdiZWdpbl95ZWFyJywgJ2VuZF95ZWFyJywgJ0JOUFAnLA0KICAgICAgICAgICAgICAgICdSRl9CTlBQJykgJT4lDQogIGZpbHRlcighaXMubmEoQk5QUCkpICU+JQ0KICBkcGx5cjo6bGVmdF9qb2luKGRhdGEuZnJhbWUodGFibGUoTlBQX2VzdGltYXRlcyA9IC5bLGMoJ3NpdGVfSUQnKV0pKSkgJT4lDQogIHJlbmFtZShCTlBQX2NvdW50ID0gRnJlcSkgJT4lDQogIG11dGF0ZSh0c19sZW5ndGggPSAxICsgKGVuZF95ZWFyIC0gYmVnaW5feWVhcikpICU+JQ0KICBtdXRhdGUodHNfbGVuZ3RoID0gcmVwbGFjZSh0c19sZW5ndGgsIGJlZ2luX3llYXIgPT0gOTk5OSwgMSkpICU+JQ0KICByb3d3aXNlKCkgJT4lDQogIG11dGF0ZSh1bmNlcnRhaW50eV9CRyA9IGlmZWxzZShiaW9tZSA9PSAnQycsIFBCX0NfQkcqUkZfQk5QUC9zcXJ0KHRzX2xlbmd0aCksDQogICAgICAgICAgICAgICAgICAgICAgICAgICAgICAgICBpZmVsc2UoYmlvbWUgPT0gJ0cnLCBQQl9HX0JHKlJGX0JOUFAvc3FydCh0c19sZW5ndGgpLCBOQSkpKSAlPiUNCiAgZ3JvdXBfYnkoc2l0ZV9JRCkgJT4lDQogIHJlZnJhbWUoQk5QUCA9IG1lYW4oQk5QUCwgbmEucm0gPSBUKSwgDQogICAgICAgICAgdW5jZXJ0YWludHlfQkcgPSBzcXJ0KHN1bSgodW5jZXJ0YWludHlfQkcpXjIsIG5hLnJtID0gVCkpL0JOUFBfY291bnQpICU+JQ0KICBkaXN0aW5jdCgpDQoNCiMjIFBlYXRsYW5kIGFuZCBUdW5kcmENCkJOUFBfcGVhdF90dW5kIDwtIE5QUF9lc3RpbWF0ZXMgJT4lDQogIGZpbHRlcihiaW9tZSAlaW4lIGMoJ1AnLCAnVCcpKSAlPiUNCiAgZHBseXI6OnNlbGVjdCgnYmlvbWUnLCAnc2l0ZV9JRCcsICdiZWdpbl95ZWFyJywgJ2VuZF95ZWFyJywgJ2ZpbmVfcm9vdHMnLCAnY29hcnNlX3Jvb3RzJywnQk5QUCcsDQogICAgICAgICAgICAgICAgJ1JGX0JOUFAnKSAlPiUNCiAgZmlsdGVyKCEoaXMubmEoQk5QUCkgJiBpcy5uYShmaW5lX3Jvb3RzKSAmIGlzLm5hKGNvYXJzZV9yb290cykpKSAlPiUNCiAgZmlsdGVyKCFpcy5uYShSRl9CTlBQKSkgJT4lDQogIGRwbHlyOjpsZWZ0X2pvaW4oZGF0YS5mcmFtZSh0YWJsZShOUFBfZXN0aW1hdGVzID0gLlssYygnc2l0ZV9JRCcpXSkpKSAlPiUNCiAgcmVuYW1lKEJOUFBfY291bnQgPSBGcmVxKSAlPiUNCiAgbXV0YXRlKHRzX2xlbmd0aCA9IDEgKyAoZW5kX3llYXIgLSBiZWdpbl95ZWFyKSkgJT4lDQogIG11dGF0ZSh0c19sZW5ndGggPSByZXBsYWNlKHRzX2xlbmd0aCwgYmVnaW5feWVhciA9PSA5OTk5LCAxKSkgJT4lDQogIHJvd3dpc2UoKSAlPiUNCiAgbXV0YXRlKEJOUFAgPSByZXBsYWNlKEJOUFAsIGlzLm5hKEJOUFApICYgIWlzLm5hKGZpbmVfcm9vdHMpLCBzdW0oY29hcnNlX3Jvb3RzLGZpbmVfcm9vdHMsIG5hLnJtID1UKSkpICU+JQ0KICBtdXRhdGUodW5jZXJ0YWludHlfQkcgPSBpZmVsc2UoYmlvbWUgPT0gJ1AnLCBQQl9QX0JHKlJGX0JOUFAvc3FydCh0c19sZW5ndGgpLA0KICAgICAgICAgICAgICAgICAgICAgICAgICAgICAgICAgaWZlbHNlKGJpb21lID09ICdUJywgUEJfVF9CRypSRl9CTlBQL3NxcnQodHNfbGVuZ3RoKSwgTkEpKSkgJT4lDQogIGdyb3VwX2J5KHNpdGVfSUQpICU+JQ0KICByZWZyYW1lKEJOUFAgPSBtZWFuKEJOUFAsIG5hLnJtID0gVCksIA0KICAgICAgICAgIHVuY2VydGFpbnR5X0JHID0gc3FydChzdW0oKHVuY2VydGFpbnR5X0JHKV4yLCBuYS5ybSA9IFQpKS9CTlBQX2NvdW50KSAlPiUNCiAgZGlzdGluY3QoKSAlPiUNCiAgbXV0YXRlX2lmKGlzLm51bWVyaWMsIGxpc3Qofm5hX2lmKC4sIDAuMDAwMCkpKSU+JQ0KICBtdXRhdGVfaWYoaXMubnVtZXJpYywgbGlzdCh+bmFfaWYoLiwgTmFOKSkpIA0KDQoNCiMjIEZvcmVzdCBhbmQgU2hydWJsYW5kIA0KQk5QUF9mb3JlX3NocnViIDwtIE5QUF9lc3RpbWF0ZXMgJT4lDQogIGZpbHRlcihiaW9tZSAlaW4lIGMoJ0YnLCAnU2gnKSkgJT4lDQogIGRwbHlyOjpzZWxlY3QoJ2Jpb21lJywgJ3NpdGVfSUQnLCAnYmVnaW5feWVhcicsICdlbmRfeWVhcicsICdmaW5lX3Jvb3RzJywgJ2NvYXJzZV9yb290cycsJ0JOUFAnLA0KICAgICAgICAgICAgICAgICdSRl9CTlBQJykgJT4lDQogIGZpbHRlcighKGlzLm5hKEJOUFApICYgaXMubmEoZmluZV9yb290cykpKSAlPiUNCiAgZmlsdGVyKCFpcy5uYShSRl9CTlBQKSkgJT4lDQogIGRwbHlyOjpsZWZ0X2pvaW4oZGF0YS5mcmFtZSh0YWJsZShOUFBfZXN0aW1hdGVzID0gLlssYygnc2l0ZV9JRCcpXSkpKSAlPiUNCiAgcmVuYW1lKEJOUFBfY291bnQgPSBGcmVxKSAlPiUNCiAgbXV0YXRlKHRzX2xlbmd0aCA9IDEgKyAoZW5kX3llYXIgLSBiZWdpbl95ZWFyKSkgJT4lDQogIG11dGF0ZSh0c19sZW5ndGggPSByZXBsYWNlKHRzX2xlbmd0aCwgYmVnaW5feWVhciA9PSA5OTk5LCAxKSkgJT4lDQogIHJvd3dpc2UoKSAlPiUNCiAgbXV0YXRlKEJOUFAgPSByZXBsYWNlKEJOUFAsIGlzLm5hKEJOUFApICYgIWlzLm5hKGZpbmVfcm9vdHMpLCBzdW0oY29hcnNlX3Jvb3RzLGZpbmVfcm9vdHMsIG5hLnJtID1UKSkpICU+JQ0KICBtdXRhdGUodW5jZXJ0YWludHlfQkcgPSBpZmVsc2UoYmlvbWUgPT0gJ0YnLCBQQl9GX0JHKlJGX0JOUFAvc3FydCh0c19sZW5ndGgpLA0KICAgICAgICAgICAgICAgICAgICAgICAgICAgICAgICAgaWZlbHNlKGJpb21lID09ICdTaCcsIFBCX1NoX0JHKlJGX0JOUFAvc3FydCh0c19sZW5ndGgpLCBOQSkpKSAlPiUNCiAgZ3JvdXBfYnkoc2l0ZV9JRCkgJT4lDQogIHJlZnJhbWUoQk5QUCA9IG1lYW4oQk5QUCwgbmEucm0gPSBUKSwgDQogICAgICAgICAgdW5jZXJ0YWludHlfQkcgPSBzcXJ0KHN1bSgodW5jZXJ0YWludHlfQkcpXjIsIG5hLnJtID0gVCkpL0JOUFBfY291bnQpICU+JQ0KICBkaXN0aW5jdCgpICU+JQ0KICBtdXRhdGVfaWYoaXMubnVtZXJpYywgbGlzdCh+bmFfaWYoLiwgMC4wMDAwKSkpICU+JQ0KICBtdXRhdGVfaWYoaXMubnVtZXJpYywgbGlzdCh+bmFfaWYoLiwgTmFOKSkpIA0KDQpBTlBQX2FsbCA8LSBBTlBQX2ZvcmVfc2hydWJbLCBjKCdzaXRlX0lEJywgJ0FOUFAnLCAndW5jZXJ0YWludHlfQUcnKV0gJT4lDQogIGJpbmRfcm93cyhBTlBQX2Nyb3BfZ3Jhc3NbLCBjKCdzaXRlX0lEJywgJ0FOUFAnLCAndW5jZXJ0YWludHlfQUcnKV0pICU+JQ0KICBiaW5kX3Jvd3MoQU5QUF9wZWF0X3R1bmRbLCBjKCdzaXRlX0lEJywgJ0FOUFAnLCAndW5jZXJ0YWludHlfQUcnKV0pIA0KDQpCTlBQX2FsbCA8LSBCTlBQX2ZvcmVfc2hydWJbLCBjKCdzaXRlX0lEJywgJ0JOUFAnLCAndW5jZXJ0YWludHlfQkcnKV0gJT4lDQogIGJpbmRfcm93cyhCTlBQX2Nyb3BfZ3Jhc3NbLCBjKCdzaXRlX0lEJywgJ0JOUFAnLCAndW5jZXJ0YWludHlfQkcnKV0pICU+JQ0KICBiaW5kX3Jvd3MoQk5QUF9wZWF0X3R1bmRbLCBjKCdzaXRlX0lEJywgJ0JOUFAnLCAndW5jZXJ0YWludHlfQkcnKV0pIA0KDQojTlBQIC0tLS0NCk5QUCA8LSBBTlBQX2FsbCAlPiUNCiAgbGVmdF9qb2luKEJOUFBfYWxsLCBieSA9ICdzaXRlX0lEJykgJT4lDQogIGRpc3RpbmN0KCkgJT4lDQogIG11dGF0ZSh1bmNlcnRhaW50eV9UTlBQID0gc3FydChzdW0odW5jZXJ0YWludHlfQUdeMix1bmNlcnRhaW50eV9CR14yKSkpICU+JQ0KICBtdXRhdGUoVE5QUCA9IHN1bShBTlBQLCBCTlBQLCBuYS5ybSA9IEYpKSANCg0KTlBQX290aGVyIDwtIE5QUF9lc3RpbWF0ZXMgJT4lDQogIGZpbHRlcihpcy5uYShmaW5lX3Jvb3RzKSAmIGlzLm5hKEJOUFApKSAlPiUNCiAgZmlsdGVyKGlzLm5hKGhlcmJzKSAmIGlzLm5hKHNocnVicykgJiBpcy5uYShBTlBQX2hlcmJzX3NocnVicykgJiBpcy5uYShub25fdmFzY3VsYXIpDQogICAgICAgICAmIGlzLm5hKHN0ZW0pICYgaXMubmEoQU5QUF93b29kKSAmIGlzLm5hKEFOUFBfdG90X2xpdHRlcikgJiBpcy5uYShBTlBQX3RyZWUpKSAlPiUNCiAgZmlsdGVyKCEoaXMubmEoTlBQX3RvdDEpICYgaXMubmEoTlBQX3RvdDIpKSkgJT4lDQogIGZpbHRlcighc2l0ZV9JRCAlaW4lIGMoTlBQJHNpdGVfSUQpKSAlPiUNCiAgZHBseXI6OmxlZnRfam9pbihkYXRhLmZyYW1lKHRhYmxlKE5QUF9lc3RpbWF0ZXMgPSAuWyxjKCdzaXRlX0lEJyldKSkpICU+JQ0KICByZW5hbWUoTlBQX2NvdW50ID0gRnJlcSkgJT4lDQogIG11dGF0ZSh0c19sZW5ndGggPSAxICsgKGVuZF95ZWFyIC0gYmVnaW5feWVhcikpICU+JQ0KICBtdXRhdGUodHNfbGVuZ3RoID0gcmVwbGFjZSh0c19sZW5ndGgsIGJlZ2luX3llYXIgPT0gOTk5OSwgMSkpICU+JQ0KICByb3d3aXNlKCkgJT4lDQogIG11dGF0ZShOUFBfdG90MiA9IHJlcGxhY2UoTlBQX3RvdDIsIGlzLm5hKE5QUF90b3QyKSwgc3VtKE5QUF90b3QxLCByZXByb2R1Y3RpdmVfbWF0ZXJpYWwsIGhlcmJpdm9yeSwgbmEucm0gPSBUKSkpICU+JQ0KICBtdXRhdGUodW5jZXJ0YWludHlfVE5QUCA9IGlmZWxzZShiaW9tZSA9PSAnRicgJiBBR19tZXRob2RvbG9neV9JRCAlaW4lIGMoJ1RCUDEnLCAnVEJQMicsICdUQlAzJywgJ1RCUDQnKSwgUEJfRl9UTlBQKlJGX0JOUFAvc3FydCh0c19sZW5ndGgpLA0KICAgICAgICAgICAgICAgICAgICAgICAgICAgICAgICAgICBpZmVsc2UoYmlvbWUgPT0gJ0cnICYgQUdfbWV0aG9kb2xvZ3lfSUQgJWluJSBjKCdUQlAxJywgJ1RCUDInLCAnVEJQMycsICdUQlA0JyksIFBCX0dfVE5QUCpSRl9CTlBQL3NxcnQodHNfbGVuZ3RoKSwgDQogICAgICAgICAgICAgICAgICAgICAgICAgICAgICAgICAgICAgICAgICBpZmVsc2UoYmlvbWUgPT0gJ0MnICYgQUdfbWV0aG9kb2xvZ3lfSUQgJWluJSBjKCdUQlAxJywgJ1RCUDInLCAnVEJQMycsICdUQlA0JyksIFBCX0NfVE5QUCpSRl9CTlBQL3NxcnQodHNfbGVuZ3RoKSwgDQogICAgICAgICAgICAgICAgICAgICAgICAgICAgICAgICAgICAgICAgICAgICAgICAgaWZlbHNlKGJpb21lID09ICdQJyAmIEFHX21ldGhvZG9sb2d5X0lEICVpbiUgYygnVEJQMScsICdUQlAyJywgJ1RCUDMnLCAnVEJQNCcpLCBQQl9QX1ROUFAqUkZfQk5QUC9zcXJ0KHRzX2xlbmd0aCksIA0KICAgICAgICAgICAgICAgICAgICAgICAgICAgICAgICAgICAgICAgICAgICAgICAgICAgICAgICBpZmVsc2UoYmlvbWUgPT0gJ1QnICYgQUdfbWV0aG9kb2xvZ3lfSUQgJWluJSBjKCdUQlAxJywgJ1RCUDInLCAnVEJQMycsICdUQlA0JyksIFBCX1RfVE5QUCpSRl9CTlBQL3NxcnQodHNfbGVuZ3RoKSwgTkEpKSkpKSkgJT4lDQogIG11dGF0ZSh1bmNlcnRhaW50eV9BRyA9IGlmZWxzZShiaW9tZSA9PSAnRicsIFBCX0ZfQUcqUkZfQU5QUC9zcXJ0KHRzX2xlbmd0aCksDQogICAgICAgICAgICAgICAgICAgICAgICAgICAgICAgICBpZmVsc2UoYmlvbWUgPT0gJ1MnLCBQQl9TaF9BRypSRl9BTlBQL3NxcnQodHNfbGVuZ3RoKSwgDQogICAgICAgICAgICAgICAgICAgICAgICAgICAgICAgICAgICAgICAgaWZlbHNlKGJpb21lID09ICdDJywgUEJfQ19BRypSRl9BTlBQL3NxcnQodHNfbGVuZ3RoKSwNCiAgICAgICAgICAgICAgICAgICAgICAgICAgICAgICAgICAgICAgICAgICAgICAgaWZlbHNlKGJpb21lID09ICdHJywgUEJfR19BRypSRl9BTlBQL3NxcnQodHNfbGVuZ3RoKSwNCiAgICAgICAgICAgICAgICAgICAgICAgICAgICAgICAgICAgICAgICAgICAgICAgICAgICAgIGlmZWxzZShiaW9tZSA9PSAnUCcsIFBCX1BfQUcqUkZfQU5QUC9zcXJ0KHRzX2xlbmd0aCksDQogICAgICAgICAgICAgICAgICAgICAgICAgICAgICAgICAgICAgICAgICAgICAgICAgICAgICAgICAgICAgaWZlbHNlKGJpb21lID09ICdUJywgUEJfVF9BRypSRl9BTlBQL3NxcnQodHNfbGVuZ3RoKSwgTkEpKSkpKSkpICU+JQ0KICBtdXRhdGUodW5jZXJ0YWludHlfQkcgPSBpZmVsc2UoYmlvbWUgPT0gJ0YnLCBQQl9GX0JHKlJGX0JOUFAvc3FydCh0c19sZW5ndGgpLA0KICAgICAgICAgICAgICAgICAgICAgICAgICAgICAgICAgaWZlbHNlKGJpb21lID09ICdTJywgUEJfU2hfQkcqUkZfQk5QUC9zcXJ0KHRzX2xlbmd0aCksIA0KICAgICAgICAgICAgICAgICAgICAgICAgICAgICAgICAgICAgICAgIGlmZWxzZShiaW9tZSA9PSAnQycsIFBCX0NfQkcqUkZfQk5QUC9zcXJ0KHRzX2xlbmd0aCksDQogICAgICAgICAgICAgICAgICAgICAgICAgICAgICAgICAgICAgICAgICAgICAgIGlmZWxzZShiaW9tZSA9PSAnRycsIFBCX0dfQkcqUkZfQk5QUC9zcXJ0KHRzX2xlbmd0aCksDQogICAgICAgICAgICAgICAgICAgICAgICAgICAgICAgICAgICAgICAgICAgICAgICAgICAgICBpZmVsc2UoYmlvbWUgPT0gJ1AnLCBQQl9QX0JHKlJGX0JOUFAvc3FydCh0c19sZW5ndGgpLA0KICAgICAgICAgICAgICAgICAgICAgICAgICAgICAgICAgICAgICAgICAgICAgICAgICAgICAgICAgICAgIGlmZWxzZShiaW9tZSA9PSAnVCcsIFBCX1RfQkcqUkZfQk5QUC9zcXJ0KHRzX2xlbmd0aCksIE5BKSkpKSkpKSAlPiUNCiAgbXV0YXRlKHVuY2VydGFpbnR5X1ROUFAgPSByZXBsYWNlKHVuY2VydGFpbnR5X1ROUFAsIGlzLm5hKHVuY2VydGFpbnR5X1ROUFApLA0KICAgICAgICAgICAgICAgICAgICAgICAgICAgICAgICAgICAgc3FydChzdW0odW5jZXJ0YWludHlfQUdeMix1bmNlcnRhaW50eV9CR14yKSkpKSAlPiUNCiAgZ3JvdXBfYnkoc2l0ZV9JRCkgJT4lDQogIHJlZnJhbWUoVE5QUCA9IG1lYW4oTlBQX3RvdDIsIG5hLnJtID0gVCksIA0KICAgICAgICAgIHVuY2VydGFpbnR5X1ROUFAgPSBzcXJ0KHN1bSgodW5jZXJ0YWludHlfVE5QUCleMiwgbmEucm0gPSBUKSkvTlBQX2NvdW50KSAlPiUNCiAgZGlzdGluY3QoKSAlPiUNCiAgbXV0YXRlX2lmKGlzLm51bWVyaWMsIGxpc3Qofm5hX2lmKC4sIE5hTikpKQ0KDQpOUFBfYWxsIDwtIE5QUCAlPiUNCiAgYmluZF9yb3dzKE5QUF9vdGhlcikgJT4lDQogIGxlZnRfam9pbihzaXRlX2luZm9bLGMoJ3NpdGVfSUQnLCAnYmlvbWVfY29kZScsICdjbGltYXRlX3JlZ2lvbicsICdtYW5hZ2VtZW50X2NvZGUnKV0pDQpgYGANCg0KYGBge3J9DQpOUFBfYnlfYmlvbWUgPC0gTlBQX2FsbFtjKCdzaXRlX0lEJywgJ2Jpb21lX2NvZGUnLCAnQU5QUCcsICdCTlBQJywgJ1ROUFAnKV0gJT4lDQogIHBpdm90X2xvbmdlcigzOjUsIHZhbHVlc190byA9ICdOUFAnLCBuYW1lc190byA9ICd0eXBlJykNCg0KTlBQX2J5X2NsaW1hdGUgPC0gTlBQX2FsbFtjKCdzaXRlX0lEJywgJ2NsaW1hdGVfcmVnaW9uJywgJ0FOUFAnLCAnQk5QUCcsICdUTlBQJyldICU+JQ0KICBwaXZvdF9sb25nZXIoMzo1LCB2YWx1ZXNfdG8gPSAnTlBQJywgbmFtZXNfdG8gPSAndHlwZScpDQogIA0KICBib3hwbG90X2Jpb21lIDwtZ2dwbG90KG5hLm9taXQoTlBQX2J5X2Jpb21lKSwgYWVzKHg9YmlvbWVfY29kZSwgeT1OUFAsIGZpbGw9dHlwZSkpICsNCiAgZ2VvbV9ib3hwbG90KCkgKyAgDQogIGxhYnMoeSA9ICBUZVgocicoQXZlcmFnZSBhbm51YWwgTlBQICAgW2cgbSReey0yfSR5ciReey0xfV0pJyksIHggPSAnQmlvbWUnLCBmaWxsID0gJyAnKSArDQogIHNjYWxlX2ZpbGxfbWFudWFsKHZhbHVlcyA9IGMoIiNhN2M5NTciLCAiIzdmNTUzOSIsICAiI2ZmZDYwYSIpKSArDQogIHRoZW1lX2J3KCkgK2dlb21fYm94cGxvdChsd2Q9MSkgKyB0aGVtZSh0ZXh0PWVsZW1lbnRfdGV4dChzaXplPTE1KSkNCg0KYm94cGxvdF9jbGltYXRlIDwtZ2dwbG90KG5hLm9taXQoTlBQX2J5X2NsaW1hdGUpLCBhZXMoeD1jbGltYXRlX3JlZ2lvbiwgeT1OUFAsIGZpbGw9dHlwZSkpICsNCiAgZ2VvbV9ib3hwbG90KCkgKyAgDQogIGxhYnMoeSA9IFRlWChyJyhBdmVyYWdlIGFubnVhbCBOUFAgICBbZyBtJF57LTJ9JHlyJF57LTF9XSknKSwgeCA9ICdDbGltYXRlIFJlZ2lvbicsIGZpbGwgPSAnICcpICsNCiAgc2NhbGVfZmlsbF9tYW51YWwodmFsdWVzID0gYygiI2E3Yzk1NyIsICIjN2Y1NTM5IiwgICIjZmZkNjBhIikpICsgDQogIHRoZW1lX2J3KCkgK2dlb21fYm94cGxvdChsd2Q9MSkgKyB0aGVtZSh0ZXh0PWVsZW1lbnRfdGV4dChzaXplPTE1KSkNCmBgYA0KDQpgYGB7cixmaWcud2lkdGg9MTEsZmlnLmhlaWdodD01fQ0KcGxvdChib3hwbG90X2NsaW1hdGUpDQpgYGANCg0KYGBge3IsZmlnLndpZHRoPTExLGZpZy5oZWlnaHQ9NX0NCnBsb3QoYm94cGxvdF9iaW9tZSkNCmBgYA0KDQoNCiMjIFRoZSBNaWFtaSBtb2RlbA0KDQoNCmBgYHtyLCB3YXJuaW5nID0gRkFMU0V9DQpub3JtYWxpemF0aW9uX2NvbnN0YW50IDwtIHN1bShuYS5vbWl0KE5QUF9hbGxbTlBQX2FsbCRtYW5hZ2VtZW50X2NvZGUgPT0gJ04nLF0pJHVuY2VydGFpbnR5X1ROUFApDQoNCk5QUF9NaWFtaV9Nb2RlbF90ZW1wIDwtIG5hLm9taXQoTlBQX2FsbFssIGMoJ3NpdGVfSUQnLCdUTlBQJywgJ21hbmFnZW1lbnRfY29kZScsICd1bmNlcnRhaW50eV9UTlBQJyldKSAlPiUNCiAgZmlsdGVyKG1hbmFnZW1lbnRfY29kZSA9PSAnTicpICU+JQ0KICBsZWZ0X2pvaW4oY2xpbWF0ZV9kYXRhLCBieSA9ICdzaXRlX0lEJykgJT4lDQogIG11dGF0ZSh1bmNlcnRhaW50eV9ub3JtYWxpemVkID0gdW5jZXJ0YWludHlfVE5QUC9ub3JtYWxpemF0aW9uX2NvbnN0YW50KSAlPiUNCiAgYXJyYW5nZShiaW8xKSAlPiUNCiAgZHBseXI6OnNlbGVjdCgtYyhzaXRlX0lEKSkNCg0KTlBQX01pYW1pX01vZGVsX3ByZWMgPC0gbmEub21pdChOUFBfYWxsWywgYygnc2l0ZV9JRCcsICdUTlBQJywgJ21hbmFnZW1lbnRfY29kZScsICd1bmNlcnRhaW50eV9UTlBQJyldKSAlPiUNCiAgZmlsdGVyKG1hbmFnZW1lbnRfY29kZSA9PSAnTicpICU+JQ0KICBsZWZ0X2pvaW4oY2xpbWF0ZV9kYXRhLCBieSA9ICdzaXRlX0lEJykgJT4lDQogIG11dGF0ZSh1bmNlcnRhaW50eV9ub3JtYWxpemVkID0gdW5jZXJ0YWludHlfVE5QUC9ub3JtYWxpemF0aW9uX2NvbnN0YW50ICkgJT4lDQogIGFycmFuZ2UoYmlvMTIpICU+JQ0KICBkcGx5cjo6c2VsZWN0KC1jKHNpdGVfSUQpKQ0KDQoNCk1pYW1pTW9kZWxfdGVtcCA8LSBubHMyKFROUFAgfiAzMDAwLygxK2V4cChhLWIqYmlvMSkpLCBkYXRhID0gbmEub21pdChOUFBfTWlhbWlfTW9kZWxfdGVtcCksIGxpc3QoYSA9IDEuMzE1LCBiID0gMC4xMTkpKQ0KTWlhbWlNb2RlbF9wcmVjIDwtIG5sczIoVE5QUCB+IDMwMDAqKDEtZXhwKC1hKmJpbzEyKSksIGRhdGEgPSBuYS5vbWl0KE5QUF9NaWFtaV9Nb2RlbF9wcmVjKSwgbGlzdChhID0gMC4wMDA2NjQpKQ0KDQoNCk1pYW1pTW9kZWxfdGVtcF93ZWlnaHRlZCA8LSAgbmxzMihUTlBQIH4gMzAwMC8oMStleHAoYS1iKmJpbzEpKSwgZGF0YSA9IG5hLm9taXQoTlBQX01pYW1pX01vZGVsX3RlbXApLCBsaXN0KGEgPSAxLjMxNSwgYiA9IDAuMTE5KSwgY29udHJvbCA9IG5scy5jb250cm9sKG1heGl0ZXIgPSAxMDAwMDAwLCBtaW5GYWN0b3IgPSAwLjAwMDAwMDAwMDAwNiksIHdlaWdodHMgPSAxL3VuY2VydGFpbnR5X25vcm1hbGl6ZWQpDQpNaWFtaU1vZGVsX3ByZWNfd2VpZ2h0ZWQgPC0gbmxzMihUTlBQIH4gMzAwMCooMS1leHAoLWEqYmlvMTIpKSwgZGF0YSA9IG5hLm9taXQoTlBQX01pYW1pX01vZGVsX3ByZWMpLCBsaXN0KGEgPSAwLjAwMDY2NCksIHdlaWdodHMgPSAxL3VuY2VydGFpbnR5X25vcm1hbGl6ZWQpDQoNCmZ1bmNfTWlhbWlNb2RlbF90ZW1wIDwtIGZ1bmN0aW9uKGEsYix0ZW1wKXsNCiAgcmVzID0gYygpDQogIGZvciAodCBpbiB0ZW1wKXsNCiAgICByZXNfdCA9IDMwMDAvKDErZXhwKGEtYip0KSkNCiAgICByZXMgPSBjKHJlcywgcmVzX3QpDQogIH0NCiAgDQogIHJldHVybihyZXMpDQp9DQoNCmZ1bmNfTWlhbWlNb2RlbF9wcmVjIDwtIGZ1bmN0aW9uKGMscHJlYyl7DQogIHJlcyA9IGMoKQ0KICBmb3IgKHAgaW4gcHJlYyl7DQogICAgcmVzX3AgPSAzMDAwKigxLWV4cCgtYypwKSkNCiAgICByZXMgPSBjKHJlcywgcmVzX3ApDQogIH0NCiAgcmV0dXJuKHJlcykNCn0NCg0KV2hpdHRha2VyX0xpZXRoX3RlbXAgPC0gZnVuY19NaWFtaU1vZGVsX3RlbXAoMS4zMTUsIDAuMTE5LCBuYS5vbWl0KE5QUF9NaWFtaV9Nb2RlbF90ZW1wJGJpbzEpKSANCldoaXR0YWtlcl9MaWV0aF9wcmVjIDwtIGZ1bmNfTWlhbWlNb2RlbF9wcmVjKDAuMDAwNjY0LCBuYS5vbWl0KE5QUF9NaWFtaV9Nb2RlbF9wcmVjJGJpbzEyKSkNCg0KZ2dwbG90KCBkYXRhID0gbmEub21pdChOUFBfTWlhbWlfTW9kZWxfdGVtcCksIGFlcyh4ID0gYmlvMSwgeSA9IFROUFApKSArZ2VvbV9wb2ludChjb2xvciA9ICdzbGF0ZWdyZXknKSArDQogIGxhYnMoeCA9IFRlWChyJyhNZWFuIGFubnVhbCB0ZW1wZXJhdHVyZSAkW15cY2lyYyBDXSQpJyksIHkgPSBUZVgocicoQXZlcmFnZSBhbm51YWwgTlBQIFtnIHlyICReey0xfSRtJF4yJF0pJykpICsNCiAgZ2VvbV9zZWdtZW50KGFlcyh4ID0gYmlvMSwgeGVuZCA9IGRwbHlyOjpsZWFkKGJpbzEpLCB5ZW5kID0gZHBseXI6OmxlYWQocHJlZGljdChNaWFtaU1vZGVsX3RlbXAsIG5ld2RhdGEgPSBuYS5vbWl0KE5QUF9NaWFtaV9Nb2RlbF90ZW1wKSkpLCB5ID0gcHJlZGljdChNaWFtaU1vZGVsX3RlbXAsIG5ld2RhdGEgPSBuYS5vbWl0KE5QUF9NaWFtaV9Nb2RlbF90ZW1wKSksIGNvbG9yID0gInVuLXdlaWdodGVkIiksIGxpbmV3aWR0aCA9IDEsIGxpbmV0eXBlPSAxKSArDQogIGdlb21fc2VnbWVudChhZXMoeCA9IGJpbzEsIHhlbmQgPSBkcGx5cjo6bGVhZChiaW8xKSwgeWVuZCA9IGRwbHlyOjpsZWFkKHByZWRpY3QoTWlhbWlNb2RlbF90ZW1wX3dlaWdodGVkLCBuZXdkYXRhID0gbmEub21pdChOUFBfTWlhbWlfTW9kZWxfdGVtcCkpKSwgeSA9IHByZWRpY3QoTWlhbWlNb2RlbF90ZW1wX3dlaWdodGVkLCBuZXdkYXRhID0gbmEub21pdChOUFBfTWlhbWlfTW9kZWxfdGVtcCkpLCBjb2xvciA9ICJ3ZWlnaHRlZCIpLCBsaW5ld2lkdGggPSAxLCBsaW5ldHlwZSA9IDEpICsNCiAgZ2VvbV9zZWdtZW50KGFlcyh4ID0gYmlvMSwgeGVuZCA9IGRwbHlyOjpsZWFkKGJpbzEpLCB5ZW5kID0gZHBseXI6OmxlYWQobmEub21pdChXaGl0dGFrZXJfTGlldGhfdGVtcCkpLCB5ID0gbmEub21pdChXaGl0dGFrZXJfTGlldGhfdGVtcCkgLCBjb2xvciA9ICdXaGl0dGFrZXIgJiBMaWV0aCcpLCBsaW5ld2lkdGggPSAxLCBsaW5ldHlwZT0gMSkgKw0KICBzY2FsZV9jb2xvdXJfbWFudWFsKFRlWChyJygkTlBQX1QgPSBcZnJhY3szMDAwfXsxK2Vee2FcOy1cO2JcOyBUfX0kICAgICknKSwgDQogICAgICAgICAgICAgICAgICAgICAgYnJlYWtzID0gYygidW4td2VpZ2h0ZWQiLCAid2VpZ2h0ZWQiLCAiV2hpdHRha2VyICYgTGlldGgiKSwNCiAgICAgICAgICAgICAgICAgICAgICB2YWx1ZXMgPSBjKCJ0b21hdG8iLCAidHVycXVvaXNlIiwgImJsYWNrIikpICsgdGhlbWVfYncoKSArDQogIHRoZW1lKGxlZ2VuZC5wb3NpdGlvbiA9IGMoMC4xNSwgMC44KSwgbGVnZW5kLmtleSA9IGVsZW1lbnRfYmxhbmsoKSkgKyB0aGVtZSh0ZXh0PWVsZW1lbnRfdGV4dChzaXplPTE1KSkNCmBgYA0KDQpgYGB7ciwgd2FybmluZz1GQUxTRX0NCmdncGxvdCggZGF0YSA9IG5hLm9taXQoTlBQX01pYW1pX01vZGVsX3ByZWMpLCBhZXMoeCA9IGJpbzEyLCB5ID0gVE5QUCkpICtnZW9tX3BvaW50KGNvbG9yID0gJ3NsYXRlZ3JleScpICsNCiAgZ2VvbV9zZWdtZW50KGFlcyh4ID0gYmlvMTIsIHhlbmQgPSBkcGx5cjo6bGVhZChiaW8xMiksIHllbmQgPSBkcGx5cjo6bGVhZChwcmVkaWN0KE1pYW1pTW9kZWxfcHJlYywgbmV3ZGF0YSA9IG5hLm9taXQoTlBQX01pYW1pX01vZGVsX3ByZWMpKSksIHkgPSBwcmVkaWN0KE1pYW1pTW9kZWxfcHJlYywgbmV3ZGF0YSA9IG5hLm9taXQoTlBQX01pYW1pX01vZGVsX3ByZWMpKSxjb2xvciA9ICJ1bi13ZWlnaHRlZCIpLCBsaW5ld2lkdGggPSAxLCBsaW5ldHlwZT0gMSkgKw0KICBnZW9tX3NlZ21lbnQoYWVzKHggPSBiaW8xMiwgeGVuZCA9IGRwbHlyOjpsZWFkKGJpbzEyKSwgeWVuZCA9IGRwbHlyOjpsZWFkKHByZWRpY3QoTWlhbWlNb2RlbF9wcmVjX3dlaWdodGVkLCBuZXdkYXRhID0gbmEub21pdChOUFBfTWlhbWlfTW9kZWxfcHJlYykpKSwgeSA9IHByZWRpY3QoTWlhbWlNb2RlbF9wcmVjX3dlaWdodGVkLCBuZXdkYXRhID0gbmEub21pdChOUFBfTWlhbWlfTW9kZWxfcHJlYykpLGNvbG9yID0gIndlaWdodGVkIiksIGxpbmV3aWR0aCA9IDEsIGxpbmV0eXBlID0gMSkgKw0KICBnZW9tX3NlZ21lbnQoYWVzKHggPSBiaW8xMiwgeGVuZCA9IGRwbHlyOjpsZWFkKGJpbzEyKSwgeWVuZCA9IGRwbHlyOjpsZWFkKG5hLm9taXQoV2hpdHRha2VyX0xpZXRoX3ByZWMpKSwgeSA9IG5hLm9taXQoV2hpdHRha2VyX0xpZXRoX3ByZWMpICwgY29sb3IgPSAnV2hpdHRha2VyICYgTGlldGgnKSwgbGluZXdpZHRoID0gMSwgbGluZXR5cGU9IDEpICsNCiAgbGFicyh4ID0gJ1RvdGFsIGFubnVhbCBwcmVjaXBpdGF0aW9uIFttbV0nLCB5ID0gVGVYKHInKEF2ZXJhZ2UgYW5udWFsIE5QUCBbZyB5ciAkXnstMX0kbSReMiRdKScpLCBjb2xvdXIgPSAiV2VpZ2h0ZWQ6IikgKw0KICBzY2FsZV9jb2xvdXJfbWFudWFsKFRlWChyJygkTlBQX1AgPSAzMDAwIFxsZWZ0KDEtZV57LWMgXDtQfVxyaWdodCkkICAgICAgICknKSwgDQogICAgICAgICAgICAgICAgICAgICAgYnJlYWtzID0gYygidW4td2VpZ2h0ZWQiLCAid2VpZ2h0ZWQiLCAiV2hpdHRha2VyICYgTGlldGgiKSwNCiAgICAgICAgICAgICAgICAgICAgICB2YWx1ZXMgPSBjKCJ0b21hdG8iLCAidHVycXVvaXNlIiwgImJsYWNrIikpICsgdGhlbWVfYncoKSArDQogIHRoZW1lKGxlZ2VuZC5wb3NpdGlvbiA9IGMoMC44NywgMC44NSkpICsgdGhlbWUodGV4dD1lbGVtZW50X3RleHQoc2l6ZT0xNSkpDQpgYGANCg0KYGBge3J9DQptb2RlbF9jb2VmZmljZW50cyA8LSBkYXRhLmZyYW1lKCdQYXJhbWV0ZXInID0gYygnYScsJ2InLCdjJyksDQogICAgICAgICAgICAgICAgICAgICAgICAgICAgICAgICdVbi53ZWlnaHRlZCcgPSBjKHN1bW1hcnkoTWlhbWlNb2RlbF90ZW1wKSRjb2VmWywgYygnRXN0aW1hdGUnKV1bMV0sc3VtbWFyeShNaWFtaU1vZGVsX3RlbXApJGNvZWZbLCBjKCdFc3RpbWF0ZScpXVsyXSxzdW1tYXJ5KE1pYW1pTW9kZWxfcHJlYykkY29lZlssIGMoJ0VzdGltYXRlJyldWzFdKSwNCiAgICAgICAgICAgICAgICAgICAgICAgICAgICAgICAgJ1dlaWdodGVkJyA9IGMoc3VtbWFyeShNaWFtaU1vZGVsX3RlbXBfd2VpZ2h0ZWQpJGNvZWZbLCBjKCdFc3RpbWF0ZScpXVsxXSwgc3VtbWFyeShNaWFtaU1vZGVsX3RlbXBfd2VpZ2h0ZWQpJGNvZWZbLCBjKCdFc3RpbWF0ZScpXVsyXSwgc3VtbWFyeShNaWFtaU1vZGVsX3ByZWNfd2VpZ2h0ZWQpJGNvZWZbLCBjKCdFc3RpbWF0ZScpXVsxXSkpDQpgYGANCg0KIyBDYWxjdWxhdGluZyBtYXhpbXVtIGFuZCBtaW5pbXVtIHBlcmNlbnRhZ2UgZGlmZmVyZW5jZSANCg0KYGBge3J9DQpwcmVkaWN0X3RlbXBfdW53ZWlnaHRlZCA8LSBwcmVkaWN0KE1pYW1pTW9kZWxfdGVtcCwgbmV3ZGF0YSA9IG5hLm9taXQoTlBQX01pYW1pX01vZGVsX3RlbXApKQ0KcHJlZGljdF90ZW1wX3dlaWdodGVkIDwtIHByZWRpY3QoTWlhbWlNb2RlbF90ZW1wX3dlaWdodGVkLCBuZXdkYXRhID0gbmEub21pdChOUFBfTWlhbWlfTW9kZWxfdGVtcCkpDQpwcmVkaWN0X3ByZWNfdW53ZWlnaHRlZCA8LSBwcmVkaWN0KE1pYW1pTW9kZWxfcHJlYywgbmV3ZGF0YSA9IG5hLm9taXQoTlBQX01pYW1pX01vZGVsX3ByZWMpKQ0KcHJlZGljdF9wcmVjX3dlaWdodGVkIDwtIHByZWRpY3QoTWlhbWlNb2RlbF9wcmVjX3dlaWdodGVkLCBuZXdkYXRhID0gbmEub21pdChOUFBfTWlhbWlfTW9kZWxfcHJlYykpDQoNCg0KcHJlZGljdGVkX3ZhbHVlc190ZW1wX2ZpdCA8LSBkYXRhLmZyYW1lKE5QUF90ZW1wX2ZpdCA9IHByZWRpY3RfdGVtcF91bndlaWdodGVkLA0KICAgICAgICAgICAgICAgICAgICAgICAgICAgICAgICAgICAgICAgIE5QUF90ZW1wX2ZpdF93ZWlnaHRlZCA9IHByZWRpY3RfdGVtcF93ZWlnaHRlZCkgJT4lDQogIGJpbmRfY29scyh0ZW1wID0gbmEub21pdChOUFBfTWlhbWlfTW9kZWxfdGVtcCkkYmlvMSkgJT4lDQogIHJvd3dpc2UoKSAlPiUNCiAgbXV0YXRlKE5QUF90ZW1wX2ZpdF9kaWZmID0gIE5QUF90ZW1wX2ZpdC1OUFBfdGVtcF9maXRfd2VpZ2h0ZWQpICU+JQ0KICBtdXRhdGUoTlBQX3RlbXBfZml0X3BlcmNfZGlmZiA9IChhYnMoTlBQX3RlbXBfZml0X2RpZmYpLygoTlBQX3RlbXBfZml0KSkpKjEwMCkgDQoNCnByZWRpY3RlZF92YWx1ZXNfcHJlY19maXQgPC0gZGF0YS5mcmFtZShOUFBfcHJlY19maXQgPSBwcmVkaWN0X3ByZWNfdW53ZWlnaHRlZCwNCiAgICAgICAgICAgICAgICAgICAgICAgICAgICAgICAgICAgICAgICBOUFBfcHJlY19maXRfd2VpZ2h0ZWQgPSBwcmVkaWN0X3ByZWNfd2VpZ2h0ZWQpICU+JQ0KICBiaW5kX2NvbHMocHJlYyA9IG5hLm9taXQoTlBQX01pYW1pX01vZGVsX3ByZWMpJGJpbzEyKSAlPiUNCiAgcm93d2lzZSgpICU+JQ0KICBtdXRhdGUoTlBQX3ByZWNfZml0X2RpZmYgPSAgTlBQX3ByZWNfZml0LU5QUF9wcmVjX2ZpdF93ZWlnaHRlZCkgJT4lDQogIG11dGF0ZShOUFBfcHJlY19maXRfcGVyY19kaWZmID0gKGFicyhOUFBfcHJlY19maXRfZGlmZikvKChOUFBfcHJlY19maXQpKSkqMTAwKQ0KDQpwZXJjX2RpZmZfdGVtcF9maXRzIDwtIGdncGxvdChkYXRhID0gcHJlZGljdGVkX3ZhbHVlc190ZW1wX2ZpdCkgKw0KICBnZW9tX3NlZ21lbnQoYWVzKHggPSB0ZW1wLCB4ZW5kID0gZHBseXI6OmxlYWQodGVtcCksIHllbmQgPSBkcGx5cjo6bGVhZChOUFBfdGVtcF9maXRfcGVyY19kaWZmKSwgeSA9IE5QUF90ZW1wX2ZpdF9wZXJjX2RpZmYpLCBsaW5ld2lkdGggPSAxLCBsaW5ldHlwZT0gMSkgKw0KICBsYWJzKHggPSBUZVgocicoTWVhbiBhbm51YWwgdGVtcGVyYXR1cmUgJFteXGNpcmMgQ10kKScpLA0KICAgICAgIHkgPSAnUGVyY2VudGFnZSBkaWZmZXJlbmNlIFslXScpICArIHRoZW1lX2J3KCkNCg0KDQpwZXJjX2RpZmZfcHJlY19maXRzIDwtIGdncGxvdChkYXRhID0gcHJlZGljdGVkX3ZhbHVlc19wcmVjX2ZpdCkgKw0KICBnZW9tX3NlZ21lbnQoYWVzKHggPSBwcmVjLCB4ZW5kID0gZHBseXI6OmxlYWQocHJlYyksIHllbmQgPSBkcGx5cjo6bGVhZChOUFBfcHJlY19maXRfcGVyY19kaWZmKSwgeSA9IE5QUF9wcmVjX2ZpdF9wZXJjX2RpZmYgKSwgbGluZXdpZHRoID0gMSwgbGluZXR5cGU9IDEpICsNCiAgbGFicyh4ID0gJ1RvdGFsIGFubnVhbCBwcmVjaXBpdGF0aW9uIFttbV0nLA0KICAgICAgIHkgPSAnUGVyY2VudGFnZSBkaWZmZXJlbmNlIFslXScpICArIHRoZW1lX2J3KCkNCg0KDQpnZ2FycmFuZ2UocGVyY19kaWZmX3RlbXBfZml0cywgcGVyY19kaWZmX3ByZWNfZml0cywNCiAgICAgICAgICBsYWJlbHMgPSBjKCJBIiwgIkIiKSwNCiAgICAgICAgICBuY29sID0gMiwgbnJvdyA9IDEsDQogICAgICAgICAgd2lkdGhzID0gYygwLjg1LCAxKSkNCmBgYA0KDQpgYGB7cn0NCnJzX3AxIDwtIHBsb3QoTWlhbWlNb2RlbF9wcmVjX3dlaWdodGVkLCB0aXRsZSA9ICJibCIpDQpxcV9wMSA8LSBxcW5vcm0oTWlhbWlNb2RlbF9wcmVjX3dlaWdodGVkLCBhYmxpbmUgPSBjKDAsMSkpDQpyc19wMiA8LSBwbG90KE1pYW1pTW9kZWxfcHJlYykNCnFxX3AyIDwtIHFxbm9ybShNaWFtaU1vZGVsX3ByZWMsIGFibGluZSA9IGMoMCwxKSkNCmdyaWQuYXJyYW5nZShhcnJhbmdlR3JvYihyc19wMSwgdG9wID0gJ3dlaWdodGVkJyksDQogICAgICAgICAgICAgYXJyYW5nZUdyb2IocXFfcDEsIHRvcCA9ICcgJyksDQogICAgICAgICAgICAgYXJyYW5nZUdyb2IocnNfcDIsIHRvcCA9ICAndW4td2VpZ2h0ZWQnKSwgDQogICAgICAgICAgICAgYXJyYW5nZUdyb2IocXFfcDIsIHRvcCA9ICcgJyksDQogICAgICAgICAgICAgdG9wID0gdGV4dEdyb2IoVGVYKHInKERpYWdub3N0aWMgcGxvdHMgJE5QUF9QJCknKSxncD1ncGFyKGZvbnRzaXplPTIwLGZvbnQ9MykpLCBuY29sPTIpDQpgYGANCg0KDQpgYGB7cn0NCnJzX3AzIDwtIHBsb3QoTWlhbWlNb2RlbF90ZW1wX3dlaWdodGVkKQ0KcXFfcDMgPC0gcXFub3JtKE1pYW1pTW9kZWxfdGVtcF93ZWlnaHRlZCwgYWJsaW5lID0gYygwLDEpKQ0KcnNfcDQgPC0gcGxvdChNaWFtaU1vZGVsX3RlbXApDQpxcV9wNCA8LSBxcW5vcm0oTWlhbWlNb2RlbF90ZW1wLCBhYmxpbmUgPSBjKDAsMSkpDQpncmlkLmFycmFuZ2UoYXJyYW5nZUdyb2IocnNfcDMsIHRvcCA9ICd3ZWlnaHRlZCcpLA0KICAgICAgICAgICAgIGFycmFuZ2VHcm9iKHFxX3AzLCB0b3AgPSAnICcpLA0KICAgICAgICAgICAgIGFycmFuZ2VHcm9iKHJzX3A0LCB0b3AgPSAgJ3VuLXdlaWdodGVkJyksIA0KICAgICAgICAgICAgIGFycmFuZ2VHcm9iKHFxX3A0LCB0b3AgPSAnICcpLA0KICAgICAgICAgICAgIHRvcCA9IHRleHRHcm9iKFRlWChyJyhEaWFnbm9zdGljIHBsb3RzICROUFBfVCQpJyksZ3A9Z3Bhcihmb250c2l6ZT0yMCxmb250PTMpKSwgbmNvbD0yKQ0KYGBgDQoNCg==
