# Supplementary material for: A global database of net primary production of terrestrial ecosystems
Source: Sci Data. 2025 Sep 2;12:1534. doi: 10.1038/s41597-025-05773-4 (PMC12405561; doi:10.1038/s41597-025-05773-4)
Supplement: Supplementary file 3 — Documentation file NPP DB [file 41597_2025_5773_MOESM3_ESM.pdf]

Global Net Primary Productivity Database

Documentation File

Table of Contents

Version Information **2**

V 1.1.1 2

Database structure and documentation **3**

Tables 3

Site Information 3

AG methodology 4

AG methods 4

BG methodology 5

BG methods 5

NPP estimates 5

Soil fertility estimate 7

CRU-TS climate data 8

WorldClim2 climate data 8

HWSD2 soil data 9

Nitrogen deposition 10

BioClim 10

Management classification 11

Considered methods and the error reduction coefficient (RF) **12**

Considered methods for estimating NPP and the corresponding RF 12

Methods for estimating ANPP 12

Methods for estimating BNPP 13

Methods for estimating total NPP 14

Error reduction coefficient for combined methods 14

References **15**

Documentation file 15

Database 19

Unpublished sources 19

Published sources 19

0. Version Information

V 1.1.1.

Any corrections or questions should be addressed to Marie Rodal at marie.rodal@uantwerpen.be.

1. Database structure and documentation

1.1 Tables

Site Information		
Site ID	Text	Code for the plot. First 2 letters: country. Central 3 letters: abbreviation site. Letter F or D: F indicates that the code is the same as Fluxnet (or other network as CarboEurope-IP , Ameriflux, ICOS etc.) while D indicates that the name was given here in developing the database. Last number: indicate the stand at the site location (sometimes more stands are available at the same site). Some sites additionally have a "_I", "_F" or "_C" (I: irrigated, F: fertilized, C: control) added at the end, indicating treatment experiments.
Plot Name	Text	Name (normally referring to area, city or research station nearby)
Vegetation Source	Text	Vegetation type according to the data source, thus 'not standardized' e.g. cold steppe, tropical grassland etc.
Climate region	Text	Broad classification into 5 climate regions: Temperate, Tropical, Arid, Polar and Cold. Based on the Koppen-Geiger Classification scheme[1] using temperature and precipitation data from the WorldClim (V2.1) database[2].
Climate class	Text	Broad classification into 5 climate classes: humid, dry sub-humid, semi-arid, arid and hyper arid. Based on the aridity index of ref. [3].
Biome	Text	Broad classification into 10 biomes types: forest, grassland, cropland, desert, arctic and alpine tundra, marsh, northern peatland, woodland and savanna.
Biome code	Text	The biomes in the previous column (*Biome*) is further reduced to 6 categories indicated by a one or two letter abbreviation: Forest (F), Grassland (G), Peatland (P), Shrubland (Sh), Tundra (T) and Cropland (C).
Study type	Text	FA: field assessment<br>TE: treatment experiment; refers to sites which underwent treatment (e.g. fertilization or irrigation) as part of an experiment and the corresponding control plots.<br>M: highly intensively or extensively managed site (e.g. NPP is available from harvest data)<br>PM: process-based model (calibrated for the specific site)
Dominant functional type	Text	Dominant plant functional types (10 plant functional types: deciduous tree, evergreen tree, C3 and C4 shrub, (C3) forb, C3 grass, C4 grass, nonvascular (lichens or moss), sedges and (C3) legume.) Delimiter: '+'
Dominant species	Text	Dominant species at site; for herbaceous biomes it is typically difficult to identify the dominant species and is such cases a list of species is given. Delimiter: ' ; '
Subdominant species 1	Text	Main subdominant species at site. Delimiter: ' ; '
Subdominant species 2	Text	Other species at site. Delimiter: ' ; '
Management and disturbance	Text	Relevant information on management and disturbance. Delimiter: ' ; '
Management code	Text	Un-ordered, binary category indicating whether a site is managed or unmanaged.
Latitude	Number	Latitude
North/South	Text	Indicate Southern or Northern hemisphere.
Longitude	Number	Longitude
East/West	Text	Indicate West or East with reference to the prime meridian
Decimal digits coordinates	Number	Number of decimals in latitude and longitude coordinates, indicating the degree of precision in the location estimate.

Elevation	Number	Elevation above sea level in **m**.
Reference	Text	Main publications where the plot information is available

**AG Methodology**		
AG methodology ID	Text	Unique identifier for the given aboveground methodology. The letters indicate that the vegetation is primarily herbaceous (HE), woody (WO), mixed (MIX) or non-vascular (NV).
Understory	Logical	Indicates whether an estimate for the understory (i.e. shrubs, grasses, mosses, lichens etc.) is included (TRUE) or not (FALSE) for the given methodology. For biomes dominated by herbaceous or non-vascular vegetation this is the primary above ground component.
Method ANPP herbs and shrubs	Text	Method code indicating the method used to estimate component ANPP; refer to **AG Methods** table below.
Non Vascular	Logical	Indicates whether non-vascular plants were accounted for in the ANPP estimate. NaN values refer to sites where no non-vascular vegetation is presumed present.
Method non-vascular	Text	Method code indicating the method used to estimate component ANPP; refer to **AG Methods** table below.
Overstory	Logical	Indicates whether an estimate for the overstory vegetation (i.e. trees) is included for the given methodology. TRUE if overstory is included, FALSE if overstory is missing from estimate, NaN if there is no overstory present at site.
Stem	Logical	Indicates whether the stem included in the ANPP estimate. Only filled in if *Overstory* is TRUE.
Branches	Logical	Indicates whether branches are included in the ANPP estimate. Only filled in if *Overstory* is TRUE.
Foliage	Logical	Indicates whether foliage is included in the ANPP estimate. Only filled in if *Overstory* is TRUE.
Decomp. leaves	Logical	Indicates whether the rapid decomposition of leaves in tropical sites is accounted for. Only filled in for tropical sites and whenever *Overstory* is TRUE.
Herbivory	Logical	Indicates whether herbivory is accounted for. If NaN, assume that herbivory is not accounted for.
Repro. material	Logical	Indicates whether reproductive materials were accounted for in the estimate of NPP. Only filled in when *Overstory* is TRUE. Note that often reproductive material will be counted together with leaf litter and there will not be given as a separate entry in the NPP estimates table.
RF	Number	Method specific error reduction coefficient for aboveground NPP estimate.

**AG methods**		
ID	Text	Unique identifier for each AG method group
RF	Number	Error reduction factor for a given method group
Method	Text	A brief description of the NPP methodologies considered as a part of a given AG method group. (See subsequent section on description of considered methodologies.)
References	Text	Main publications where the NPP methodology is given

BG Methodology		
BG methodology ID	Text	Unique identifier for the given belowground methodology. The letters indicate whether coarse roots are present at site (CFR) or whether all roots are assumed accounted for in the fine roots estimate (FR).
Fine roots	Logical	Indicates whether a fine root estimate is included for the given methodology.
Method BNPP fine roots	Text	Method code indicating the method used to estimate BNPP of fine roots. For non woody vegetation all roots are assumed to be fine roots; refer to BG Methods table below.
Coarse roots	Logical	Indicates whether a coarse root estimate is included for the given methodology. NaN values refer to sites where no coarse roots are presumed present (e.g. when all roots are counted as fine roots).
Method BNPP coarse roots	Text	Method code indicating the method used to estimate BNPP of coarse roots; refer to BG Methods table below.
RF	Number	Method specific error reduction coefficient for belowground NPP estimate. Take care: this RF does not account for missing components, e.g. when coarse roots are missing.

BG methods		
ID	Text	Unique identifier for each BG method group
RF	Number	Error reduction factor for a given method group
Method	Text	A brief description of the NPP methodologies considered as a part of a given BG method group. (See subsequent section on description of considered methodologies.)
References	Text	Main publications where the NPP methodology is given

NPP Estimates		
Measurement ID	Text	Unique identifier for the given measurement: First character identifies the biome type, next four characters identifies the site using the site abbreviation (see column Site ID in table **Site Information**), last number identifies the measurement number.
Site ID	Text	Site ID according to table **Site information**
Begin year	Number	First year that NPP was estimated, 9999 when not known
End year	Number	Last year of the period that NPP was estimated, use the year of publication when not known. Note that if there is less than 12 months between the begin and end of measurements, then *End year* is taken to be the same as *Begin year* even when falling in different years.
Herbs	Number	Aboveground NPP estimate in **g $yr^{-1}m^2$** for herbaceous vegetation.
Shrubs	Number	Above ground NPP estimate in **g $yr^{-1}m^2$** for shrubs.
ANPP herbs shrubs	Number	Aboveground NPP estimate in **g $yr^{-1}m^2$** including shrubs and herbaceous vegetation. Whenever no distinction is made between shrubs and herbs the recorded NPP value is entered in this column (e.g. for most croplands).
Non-vascular	Number	Aboveground NPP estimate in **g $yr^{-1}m^2$** for the non-vascular (e.g. mosses and lichens) part of the vegetation.
ANPP non tree	Number	Total aboveground NPP estimate in **g $yr^{-1}m^2$.** Whenever the preceding 3 columns are complete (i.e. no missing relevant components), ANPP non-tree is given by their sum. For forests biomes ANPP non-tree refers to the understory vegetation.

Fine roots (inc. leaching)	Number	Belowground NPP estimates in **g $yr^{-1}m^2$** for fine roots. As leaching is only available for very few sites, this has been included in the fine root estimate.
Coarse roots	Number	Belowground NPP estimates in **g $yr^{-1}m^2$** for coarse roots whenever present.
BNPP	Number	Belowground NPP in **g $yr^{-1}m^2$** of entire vegetation (herbaceous layer + any woody growth forms of overstory). It includes rhizhomes or crowns (stem bases left after harvest plus rhizomes), wherever mentioned.
Stem	Number	Estimate for stem NPP in **g $yr^{-1}m^2$** of woody vegetation not included in shrubs.
Branch	Number	Estimate for branch NPP in **g $yr^{-1}m^2$** of woody vegetation not included in shrubs.
ANPP wood	Number	Sum of stem and branch columns, and estimate for total above ground wood. Whenever branches and stem NPP values are not estimated independently, ANPP wood (in **g $yr^{-1}m^2$**) is entered in this column.
Leaf litter	Number	NPP estimate in **g $yr^{-1}m^2$** for leaf litter from woody vegetation not included in shrubs.
Extra litter	Number	NPP estimate in **g $yr^{-1}m^2$** for extra litter (e.g. small branches) not included in leaf litter.
ANPP total litterfall	Number	Sum of leaf litter and extra litter in **g $yr^{-1}m^2$.**
ANPP tree	Number	Sum *ANPP total litter* and *ANPP wood* whenever both components are reported, or total ANPP (in **g $yr^{-1}m^2$**) of the woody vegetation whenever the values for the canopy and above ground wood NPP are not reported separately.
NPP total1	Number	Total NPP in **g $yr^{-1}m^2$** (*excluding* potential loss to herbivory, reproductive materials and rapid decomposition of leaves). When belowground and aboveground (overstory and understory, i.e. *ANPP tree* and *ANPP non-tree*) NPP is given, total NPP is the sum of the two.
Reproductive material	Number	NPP estimate in **g $yr^{-1}m^2$** for reproductive material (e.g. acorns, flowers, seeds, fruits etc.) whenever these are not included in total litter.
Herbivory	Number	NPP estimate in **g $yr^{-1}m^2$** for loss to herbivory. In case of the use of grazing exclosures, herbivory is assumed to be negligible in grasslands.
NPP total2	Number	Total NPP in **g $yr^{-1}m^2$** (*including* potential loss to herbivory, reproductive materials and rapid decomposition of leaves). Whenever all components are accounted for, it is the sum of *NPP total1*, decomposing leaves, reproductive material and (loss to) herbivory.
Carbon Content	Number	(Average) carbon content of all plant parts as given in original publication, or assumed whenever converted from gC to g (see **Comment 1.1** below).
AG methodology ID	Text	Identifier linking each entry in NPP estimates table to a above ground methodology and corresponding uncertainty reduction factor (see table AG methodology)
BG methodology ID	Text	Identifier linking each entry in NPP estimates table to a aboveground methodology and corresponding uncertainty reduction factor (see table BG methodology)
References	Number	Main publications where the NPP values are available

Comment 1.1 – Carbon conversion factor: Whenever the NPP data in the original publication was reported in grams of carbon as opposed to grams of dry weight, a conversion factor of 0.45 has been used for herbaceous biomes (grasslands, croplands, tundra and peatlands), while a conversion factor of 0.5 has been used for woody biomes (forests and shrubland), unless otherwise stated (e.g. when the authors of the original publication provide a conversion factor of their own). The same conversion factor has been used for all for all components of NPP; since future analysis using the database will in all likelihood be done using units of grams of carbon, the separate conversion of each component was deemed unnecessary.

**Soil Fertility Estimate**		
Site ID	Text	Site ID according to table **Site information**
Fertility status	Text	Three level category (L: low, M: medium, H: high) indicating fertility status, based on summary information gathered from various sources; see column Summary remarks.
Soil type	Text	Soil type (e.g. luvisol), but based on different soil classification schemes and thus not standardized.
Texture	Text	Soil texture, primarily based on the USDA soil texture classification, unless other classification scheme was used in the reference material.
Organic layer thickness	Number	Thickness of the organic layer (or peat, whenever the relevant site is a peatland). Measured in cm. Whenever a range is given, the mean value is reported.
Sand	Number	Sand content [%]
Clay	Number	Clay content [%]
N (min, max, mean)	Number	Nitrogen content [%] divided into three columns for min, max and mean.
C (min, max, mean)	Number	Carbon content [%] divided into three columns for min, max and mean.
CN_ratio (min, max, mean)	Number	Ratio of carbon to nitrogen in soil, divided into three columns for min, max and mean.
pH (min, max, mean)	Number	pH value, divided into three columns for min, max and mean. Note that the methods for obtaining this may differ; in particular, the type of solution (e.g. H_2O or $CaCl_2$) used to measure pH differs. Thus, the user should consult the column *Additional Information* which indicates how the value was obtained whenever this information was reported in the original reference.
CEC (min, max, mean)	Number	Cation exchange capacity, divided into three columns for min, max and mean.
Fertilization status	Text	Categorical variable indicating whether the site has been fertilized or not (FE – fertilized, UF – unfertilized, SL- slashes, NI – no information).
Additional information	Text	Remarks pertaining to how measurements where obtained, what soil layer they refer to, whether they refer to only the mineral or organic layer and additional information on the soil (e.g. water draining capacity, presence of permafrost etc.) which is not included elsewhere.
Summary remarks	Text	A brief summary of the information gathered on the fertility status of the site.
References	Text	Main publications where the information on soil texture and nutrient status was initially reported.

Comment 1.2 – Limitations of collected soil information: The information included in the soil fertility index table is, due to the nature by which it was gathered, very heterogeneous. Thus, the information is not intended for use as is, but rather as an overall indication of soil health and as justification for the derived fertility status. The information in the table can also be used to corroborate or correct data collected from global or regional soil databases for the sites in the database.

CRU-TS climate dataset		
Site ID	Text	Site ID according to table **Site information**
Year	Number	Measurement year
Month	Text	Measurement month
Cld	Number	Percentage cloud cover [%]
Frs	Number	Number of frost days [days]
Dtr	Number	Diurnal temperature range [°C]
Pet	Number	Potential evapo-transpiration [mm/day]
Pre	Number	Precipitation [mm]
Tmn	Number	Minimum Temperature [°C]
Tmp	Number	Average Temperature [°C]
Tmx	Number	Maximum Temperature [°C]
Vap	Number	Vapor pressure [hPa]
Wet	Number	Number of wet days [days]
Spei12	Number	Aridity index SPEI (the Standardized Precipitation-Evaporation Index) derived from the water deficit WD = PRE - PET

Comment 1.3 – Reference CRU-TS: The primary reference for the CRU-TS dataset is ref. [4]; extensive information on the methodology is available therein. The complete database can be downloaded from CRU-TS-V4.07. We also include an aridity index (SPEI)[5]. SPEI comes in at different temporal scales ranging from 1 to 48 months with longer temporal scales being better suited to the detection of long term drought conditions. The complete database can be downloaded from SPEI-DB.

WorldClim2 climate data		
Site ID	Text	Site ID according to table **Site information**
Month	Text	Measurement month
Tavg	Number	Long term average monthly temperature [°C]
Tmax	Number	Long term maximum monthly temperature [°C]
Tmin	Number	Long term minimum monthly temperature [°C]
Prec	Number	Long term total monthly precipitation [mm]
Srad	Number	Long term total monthly radiation [MJ m^{-2}]
Vapr	Number	Long term average monthly vapor pressure [kPa]
wind	Number	Long term average wind speed [m/s]
PET	Number	Long term average monthly PET [mm/month]
AI	Number	Long term monthly values for the aridity index defined as AI = PRE/PET

Comment 1.4 – Reference WorldClim2 - : The primary reference for the WorldClim2 dataset is ref. [2]. The complete database can be downloaded from WorldClimV2.1. The monthly average PET and AI values are from ref. [3], and can be downloaded from Global-ET0V3.0.

HWSD2 top soil data		
Site ID	Text	Site ID according to table **Site information**
HWSD2	Number	Soil classification identification number from HWSD2[6].
sequence	Number	Number typically from 1 to 5 indicating the ordering of the soil types within the soil layer (sequence 1 is always the dominant soil type as measured in terms of fraction of soil layer).
share	Number	Share of soil as a fraction of the total soil in the soil layer.
FAO90	Text	Soil classification according to FAO90
AWC	Number	Available water capacity [mm/m]
Coarse	Number	Percentage content of coarse fragments (soil particles with a nominal diameter above 2mm) [% volume]
Sand	Number	Percentage sand content [% weight]
Silt	Number	Percentage silt content [% weight]
Clay	Number	Percentage clay content [% weight]
Texture USDA	Text	Texture class according to the USDA soil texture classification
Bulk	Number	Bulk density [g/cm^3].
Ref. bulk	Number	Reference bulk density [g/cm^3].
Org. carbon	Number	Organic carbon content of the soil [% weight].
pH water	Number	pH of soil as measured in a solution of H2O [-log(H^+)]
Total N	Number	Total nitrogen content. [g/kg]
CN ratio	Number	C:N ratio.
CEC_{soil}	Number	$cmol_c/kg$
CEC_{clay}	Number	$cmol_c/kg$
CEC_{eff}	Number	$cmol_c/kg$
TEB	Number	Total exchangeable bases.
bsat	Number	Base saturation. Measures the sum of exchangeable cations Na++, Ca++, Mg++ and K+ as a percentage of the overall exchange capacity of the soil. [% of CEC_{soil}]
Alum sat	Number	Amount of exchangeable aluminum [% of CEC_{EFF}]
ESP	Number	Percentage of exchangeable sodium [%]
tCarbon_eq	Number	Percentage calcium carbonate [% weight]
gypsum	Number	Percentage gypsum content [% weight]
Elec. Cond.	Number	Electric conductivity [dS/m]

Comment 1.5 – Reference HWSD2: The primary reference for the HWSD2 database is ref. [6]. The complete database can be downloaded from HWSD2-DB.

Nitrogen deposition data		
Site ID	Text	Site ID according to table **Site information**
year	Number	Measurement month
Organic	number	Organic nitrogen deposition [kgN y^{-1} km^{-2}]
Reduced	Number	Reduced nitrogen deposition [kgN y^{-1} km^{-2}]
Oxidized	Number	Oxidized nitrogen deposition [kgN y^{-1} km^{-2}]
Inorganic	Number	Inorganic nitrogen deposition [kgN y^{-1} km^{-2}]
Total Ndep	Number	Total nitrogen deposition; sum of the organic, reduced, oxidized and inorganic columns. [kgN y^{-1} km^{-2}]

Comment 1.6 – Reference GlobalNdep: The primary reference for the Nitrogen deposition data is ref. [7]. The complete dataset can be downloaded from GlobalNdepV1.0.

BioClimatic Variables		
Site ID	Text	Site ID according to table **Site information**
Bio1	Number	Annual mean temperature [°C]
Bio2	Number	Mean diurnal range (mean of monthly (max temp – min temp)) [°C]
Bio3	Number	Isothermality (BIO2/BIO7) (x100)
Bio4	Number	Temperature seasonality (standard deviation x 100) [°C]
Bio5	Number	Max temperature of warmest month [°C]
Bio6	Number	Min temperature of coldest month [°C]
Bio7	Number	Temperature annual range (Bio5 – Bio6) [°C]
Bio8	Number	Mean temperature of wettest quarter [°C]
Bio9	Number	Mean temperature of driest quarter [°C]
Bio10	Number	Mean temperature of warmest quarter [°C]
Bio11	Number	Mean temperature of coldest quarter [°C]
Bio12	Number	Annual precipitation [mm]
Bio13	Number	Precipitation of wettest month [mm]
Bio14	Number	Precipitation of driest month [mm]
Bio15	Number	Precipitation seasonality (coefficient of variation)
Bio16	Number	Precipitation of wettest quarter [mm]
Bio17	Number	Precipitation of driest quarter [mm]
Bio18	Number	Precipitation of warmest quarter [mm]
Bio19	Number	Precipitation of coldest quarter [mm]
PET	Number	Potential evapo-transpiration (reference: Zomer and Trabucco 2022) [mm]
AI	Number	Aridity index defined as AI = PRE/PET (reference: Zomer and Trabucco 2022)

Comment 1.7 – Reference BioClim: The primary reference for the bioclimatic variables is ref. [2]. The complete database can be downloaded from WorldClimV2.1. The yearly average PET and AI values are from ref. [3], and can be downloaded from Global-ET0V3.0.

1.2 Management classification

Biome	Description	Management classification
Forest	Fertilized in the last 20 years<br>Thinned in the last 50 years<br>Planted less than 10 years before measurements<br>Established plantations managed for fruit or rubber production	Managed
	Old-growth<br>Not managed in last 50 years<br>Planted more than 10 years before measurements with minimal anthropogenic impact since	Unmanaged
Grassland	Fertilized<br>Irrigated<br>Planted on agricultural land same year as measurements	Managed
	Pristine<br>Low to intermediate grazing or mowing<br>Yearly burns	Unmanaged
Dry shrubland	Fertilized	Managed
	Pristine<br>Low to intermediate grazing<br>Yearly burns	Unmanaged
Peatland/Tundra	Fertilized (a)	Managed
	Pristine	Unmanaged
Cropland		Managed

(a) Refers to manipulative experiments

2. Considered methods and the error reduction coefficient (RF)

2.1 Considered methods for estimating NPP and the corresponding RF

Description of methodologies for estimating ANPP				
**ID**	**RF**	**Methodology**	**Remarks**	**References**
AG1	0.1	Isotope turnover/radioactive element dating		Ref. [8]
AG2	0.2	Sum of increments in live and dead biomass with adjustment for decomposition		Ref. [9] (methods 5, 6 and 7)
AG3	0.3	Sum of increments in live biomass		Ref. [9] (method 4)
AG4	0.4	Peak standing crop/maximum minus minimum live biomass/Peak standing crop adjusted with turnover coefficients/difference between annual harvests		Ref. [9] (method 1 and 2)
AG5	0.4	Allometric/biometric methods		Refs.[10,11]
AG6	0.8	Fixed proportion of other BP components	Only appropriate when not the main component, e.g. understory in a forest	Ref. [12]
NC	1.0	Not clear.	If the primary above ground component, which varies from biome to biome, is labeled NC then the RF for ANPP is 1.0 regardless of the accuracy of the measurements of other aboveground components (see Section 2.2).	
ACT	-	Accounted for with trees	Refers typically to understory in shrublands or non-vascular in e.g. peatlands or tundra sites; only relevant when not main component.	
WO	-	Woody biome (for understory in forests)	Indicates that the biome is a forest or other woody biome, and that the understory is accounted for but that the methodology is irrelevant for the estimation of RF for ANPP	

Description of methodologies for estimating BNPP				
**ID**	**RF**	**BNPP Methodology**	**Remarks**	**References**
BG1	0.2	Isotope turnover/radioactive element dating	Does not include isotope dilution, as this latter method is deemed a too in-accurate method due to violation of the assumption of homogenous labeling (see ref. [13]).	Refs. [13,14]
BG2	0.3	Minirhizotron or root windows		Refs. [10,13,15]
BG3	0.3	Ingrowth core/ingrowth donuts/mesh bag		Refs. [13,15]
BG4	0.6	Allometric methods		Ref. [10]
BG5	0.6	Carbon budget and mass balance approaches.	Includes the Raich and Nadelhoffer approximation[16], TBCA / TBCF[17] and the mass balance approach presented in ref. [18]	Refs. [10,15,17]
BG6	0.7	Sequential coring using the following methods:<br>1. Sum of increments in live and dead biomass<br>2. Sum of changes in live and dead biomass with adjustment for decomposition		Refs. [19–21]
BG7	0.8	Sequential coring using the following methods:<br>1. Peak standing crop/peak biomass<br>2. Maximum minus minimum standing total biomass<br>3. Peak total biomass corrected with turnover coefficients (see Remark 2 and 3 below)<br>4. Sum of increments in live biomass<br>5. Sum of increments in total (live + dead) biomass	Sequential coring also refers to cases where the given site is only sampled once and peak is assumed (e.g. sampling takes place at end of growing season and turnover is assumed to be 1 year).	Refs. [13,15,22]
BG8	0.8/ 1.0	Fixed proportion of ANPP stem, ANPP wood, total ANPP etc. (RF is 0.8 coarse roots, 1.0 fine roots). Examples include:<br>1. 21% of ANPP stem[23]<br>2. 21 % BNPP coarse; for fine roots[24]	Whenever this methodology is applied to the fine root component of BNPP, then the RF for BNPP is 1.0 regardless of the accuracy of the coarse root estimate.	Ref. [25]
NC	0.8/ 1.0	Not clear (RF is 0.8 for coarse roots and 1.0 for fine roots).	If the methodology for estimating fine roots is labeled NC, then the RF for BNPP is 1.0 regardless of the accuracy of the coarse root estimate.	

Comment 2.1 - RF for forested biomes: Given that the methods for estimating aboveground production in forests is generally the same (i.e. allometric relations and litter traps) the RF for the aboveground part in forested biomes is 0.4. The big difference in the assumed accuracy of estimates for forest biomes comes from the belowground component (in particular the fine roots estimate) and the estimation of missing components (e.g. understory, herbivory, reproductive material and loss due to rapid decomposition of leaves in tropical sites). The reason for putting the emphasis on below ground (in particular fine root growth) is that this is the largest source of uncertainty for NPP measurements in forests[15]. This emphasis follows the method of ref. [26] where NPP estimates were given as RF of 0.3 if both fine and coarse roots were measured, as opposed to estimated, using e.g. ingrowth cores, soil cores or minirhizotrons, or allometric relations for coarse roots, while for sites where either component was estimated (see method BG8) RF was 0.6. We deviate slightly from this approach; in particular we provide stricter criteria for what methods are considered good enough to warrant a RF of 0.3 (e.g. sequential coring methods are given a much higher uncertainty unless additional measurements using more accurate methods was used to estimate e.g. turnover coefficients), hence our uncertainty estimates, in particular for the below ground component, will generally be higher than those provided in ref. [26]. This is primarily to ensure consistency in the uncertainty estimates across biome types, and to provide a more accurate representation of the method specific uncertainty for fine root estimation.

Comment 2.2 - Below ground biomass and turnover: In contrast to ref. [26], we have chosen to give sites where BNPP of fine roots was measured from estimates of belowground biomass adjusted with turnover coefficients a comparatively high RF. This is because, based on work by e.g. ref. [13], sequential coring is deemed to be an inaccurate method, although it remains the most frequently used as it is less labor intensive than other methods. The methods included in BG8.3 refer to all methods where BNPP is obtained from multiplying biomass with a turnover coefficient. This also includes those where the regression equation of [27], which relates soil nitrogen availability to the turnover time of fine roots, has been employed (as e.g. in ref. [28]), and sites where the turnover coefficient is an estimate taken from other studies without a clear reference to methodology, even when the estimate is site specific, as in ref. [29]. Only whenever turnover was measured on site within a reasonable time frame (to ensure similar climatic conditions and avoid issues related to increasing stand age or changes in species composition) using a more accurate method, as in ref. [30], is the RF reduced.

Description of methodologies for estimating total NPP				
**ID**	**RF**	**Methodology**	**Remarks**	**Primary references**
TBP1	0.6	Radiocarbon		Refs. [31–33]
TBP2	0.6	Peat formation		Ref. [34]
TBP3	0.6	Process based model	Only process based models which are calibrated and evaluated for the specific site are considered.	Refs. [35–37]
TBP4	1.0	Flux components, including methods like:<br>1. GPP - Ra [38]<br>2. NEE + Rh [39]		Refs. [38,39]

2.2 Error reduction coefficient for combined methods

Reduction factor for combined methods. For sites where standing fine or coarse root biomass (from soil cores; see BG7 and BG8) has been combined with turnover AND turnover has been measured at the site using e.g. ingrowth

cores and minirhizotrons, the sites have been labeled as e.g. BG8 + BG3. The uncertainty reduction factor, RF, for such sites is then given as 0.6, which is the (conservative) average of the two methods. Thus, the total uncertainty is assumed to be lower than for sites where the turnover rate is simply assumed.

Primary aboveground components. Which component of ANPP determines the overall RF depends primarily on the biome, but may also depend on the specific site. Generally, for non-forested biomes the primary above ground components are herbs and shrubs, with non-vascular being an important component in a few tundra and peatland sites. For most of these sites the RF for ANPP will be determined from these components alone, with overstory biomass assumed to be less important (often it is also not measured). For some sites, e.g. NO-ha1-D01 (classified as tundra), the overstory, consisting of willow trees, represents just under 40% of ANPP, and therefore the RF is an average of the methods for both over- and understory. This information is summarized in the table below.

Combining RF for fine and coarse roots. As already mentioned in Comment 2.1, fine roots are the biggest source of uncertainty of BNPP. Thus, when providing an RF for BNPP, taking into account both coarse and fine roots, the emphasis is put on the fine root estimate. That means that while the RF's of the two belowground components are averaged, the averaging is always in favor of the fine roots (numbers are rounded to one decimal place). Hence, if the fine root method is given by the ingrowth core method (RF = 0.3), while the coarse root method is given by allometric relations BG4 (RF = 0.6), then the combined method has an RF of 0.4. This information is summarized in the table below.

Determination of RF for AG and BG production					
Aboveground				Belowground	
Primarily herbaceous	Significant non-vascular presence	Primarily woody	Mix herbaceous and woody	Fine roots only	Coarse roots present
RF equals RF of method used to estimate herbaceous NPP	RF equals the mean of the RF of the non-vascular and herbaceous components	As ANPP of all the woody sites in the database is estimated using allometric relations and litter traps, they are all given the same RF (0.4)	RF equals the mean of the RF of the herbaceous (understory) and woody (overstory) component methods.	RF equals the RF of the fine roots method.	RF equals the mean of the RF of the coarse and fine roots methods.

References

Documentation file

1. Peel, M. C., Finlayson, B. L. & McMahon, T. A. Updated world map of the Köppen-Geiger climate classification. *Hydrol Earth Syst Sci* (2007).

2. Fick, S. E. & Hijmans, R. J. WorldClim 2: new 1-km spatial resolution climate surfaces for global land areas. *Int. J. Climatol.* **37**, 4302–4315 (2017).

3. Zomer, R. J. & Trabucco, A. Global Aridity Index and Potential Evapo-Transpiration (ET0) Database v3. (2022).
4. Harris, I., Osborn, T. J., Jones, P. & Lister, D. Version 4 of the CRU TS monthly high-resolution gridded multivariate climate dataset. *Sci. Data* **7**, 109 (2020).
5. Vicente-Serrano, S. M., Beguería, S. & López-Moreno, J. I. A Multiscalar Drought Index Sensitive to Global Warming: The Standardized Precipitation Evapotranspiration Index. *J. Clim.* **23**, 1696–1718 (2010).
6. *Harmonized World Soil Database Version 2.0*. (FAO; International Institute for Applied Systems Analysis (IIASA);, 2023). doi:10.4060/cc3823en.
7. Ackerman, D., Millet, D. B. & Chen, X. Global Estimates of Inorganic Nitrogen Deposition Across Four Decades. *Glob. Biogeochem. Cycles* **33**, 100–107 (2019).
8. Milchunas, D. G. & Lauenroth, W. K. Carbon Dynamics and Estimates of Primary Production by Harvest, ^(14)C Dilution, and ^(14)C Turnover. *Ecology* **73**, 593–607 (1992).
9. Scurlock, J. M. O., Johnson, K. & Olson, R. J. Estimating net primary productivity from grassland biomass dynamics measurements. *Glob. Change Biol.* **8**, 736–753 (2002).
10. Clark, D. A. *et al.* Measuring net primary production in forests: concepts and field methods. *Ecol. Appl.* **11**, (2001).
11. Ladwig, L. M. *et al.* Above- and belowground responses to nitrogen addition in a Chihuahuan Desert grassland. *Oecologia* **169**, 177–185 (2012).
12. Keith, H. *et al.* Multiple measurements constrain estimates of net carbon exchange by a Eucalyptus forest. *Agric. For. Meteorol.* **149**, 535–558 (2009).
13. Milchunas, D. G. Estimating Root Production: Comparison of 11 Methods in Shortgrass Steppe and Review of Biases. *Ecosystems* **12**, 1381–1402 (2009).

14. Gaudinski, J. B., Trumbore, S. E., Davidson, E. A. & Zheng, S. Soil carbon cycling in a temperate forest: radiocarbon-based estimates of residence times, sequestration rates and partitioning of fluxes. *Biogeochemistry* **51**, 53–69 (2000).

15. Vogt, K. A., Vogt, D. J. & Bloomfield, J. Analysis of some direct and indirect methods for estimating root biomass and production of forests at an ecosystem level. in *Root Demographics and Their Efficiencies in Sustainable Agriculture, Grasslands and Forest Ecosystems* (ed. Box, J. E.) 687–720 (Springer Netherlands, Dordrecht, 1998). doi:10.1007/978-94-011-5270-9_61.

16. Raich, J. W. & Nadelhoffer, K. J. Belowground Carbon Allocation in Forest Ecosystems: Global Trends. *Ecology* **70**, 1346–1354 (1989).

17. Giardina, C. P. & Ryan, M. G. Total Belowground Carbon Allocation in a Fast-growing Eucalyptus Plantation Estimated Using a Carbon Balance Approach. *Ecosystems* **5**, 487–499 (2002).

18. Fenn, K., Malhi, Y., Morecroft, M., Lloyd, C. & Thomas, M. *Comprehensive Description of the Carbon Cycle of an Ancient Temperate Broadleaved Woodland*. https://bg.copernicus.org/preprints/7/3735/2010/ (2010) doi:10.5194/bgd-7-3735-2010.

19. Pérez, C. A. & Frangi, J. L. Grassland biomass dynamics along an altitudinal gradient in the Pampa. *J. RANGE Manag.* (2000).

20. San Jose, J., Montes, R., Grace, J. & Nikonova, N. Land-use changes alter CO2 flux patterns of a tall-grass Andropogon field and a savanna-woodland continuum in the Orinoco lowlands. *Tree Physiol.* **28**, 437–450 (2008).

21. Konôpka, B., Yuste, J. C., Janssens, I. A. & Ceulemans, R. Comparison of Fine Root Dynamics in Scots Pine and Pedunculate Oak in Sandy Soil. *Plant Soil* **276**, 33–45 (2005).

22. McClaugherty, C. A., Aber, J. D. & Melillo, J. M. The Role of Fine Roots in the Organic Matter and Nitrogen Budgets of Two Forested Ecosystems. *Ecology* **63**, 1481–1490 (1982).

23. Malhi, Y. *et al.* Comprehensive assessment of carbon productivity, allocation and storage in three Amazonian forests. *Glob. Change Biol.* **15**, 1255–1274 (2009).

24. Noormets, A. *et al.* Response of carbon fluxes to drought in a coastal plain loblolly pine forest. *Glob. Change Biol.* **16**, 272–287 (2010).

25. Aragão, L. E. O. C. *et al.* Above- and below-ground net primary productivity across ten Amazonian forests on contrasting soils. *Biogeosciences* **6**, 2759–2778 (2009).

26. Luyssaert, S. *et al.* CO_2 balance of boreal, temperate, and tropical forests derived from a global database. *Glob. Change Biol.* **13**, 2509–2537 (2007).

27. Aber, J. D., Melillo, J. M., Nadelhoffer, K. J., McClaugherty, C. A. & Pastor, J. Fine root turnover in forest ecosystems in relation to quantity and form of nitrogen availability: a comparison of two methods. *Oecologia* **66**, 317–321 (1985).

28. Ehman, J. L. *et al.* An initial intercomparison of micrometeorological and ecological inventory estimates of carbon exchange in a mid-latitude deciduous forest. *Glob. Change Biol.* **8**, 575–589 (2002).

29. Marsden, C. *et al.* Modifying the G'DAY process-based model to simulate the spatial variability of Eucalyptus plantation growth on deep tropical soils. *For. Ecol. Manag.* **301**, 112–128 (2013).

30. Law, B. E. *et al.* Disturbance and climate effects on carbon stocks and fluxes across Western Oregon USA. *Glob. Change Biol.* **10**, 1429–1444 (2004).

31. Wieder, R. K. Past, Present, and Future Peatland Carbon Balance: An Empirical Model Based on 210 Pb-Dated Cores. *Ecol. Appl.* **11**, 327 (2001).

32. Jenkinson, D. S. *et al.* Estimating net primary production from measurements made on soil organic matter. **80**, (1999).

33. Trumbore, S. E., Bubier, J. L., Harden, J. W. & Crill, P. M. Carbon cycling in boreal wetlands: A comparison of three approaches. *J. Geophys. Res. Atmospheres* **104**, 27673–27682 (1999).

34. Malmer, N., Johansson, T., Olsrud, M. & Christensen, T. R. Vegetation, climatic changes and net carbon sequestration in a North-Scandinavian subarctic mire over 30 years. *Glob. Change Biol.* **11**, 1895–1909 (2005).
35. Grant, R. F., Oechel, W. C. & Ping, C. Modelling carbon balances of coastal arctic tundra under changing climate. *Glob. Change Biol.* **9**, 16–36 (2003).
36. Wang, Q., Watanabe, M. & Ouyang, Z. Simulation of water and carbon fluxes using BIOME-BGC model over crops in China. *Agric. For. Meteorol.* **131**, 209–224 (2005).
37. Hunt, H. W. *et al.* Simulation model for the effects of climate change on temperate grassland ecosystems. *Ecol. Model.* **53**, 205–246 (1991).
38. Griffis, T. J. *et al.* Seasonal variation and partitioning of ecosystem respiration in a southern boreal aspen forest. *Agric. For. Meteorol.* **125**, 207–223 (2004).
39. Lagergren, F. *et al.* Net primary production and light use efficiency in a mixed coniferous forest in Sweden. *Plant Cell Environ.* **28**, 412–423 (2005).

Database sources

Database unpublished sources

Pers. Commun. Alberti

Pers. Commun. Bernhofer

Pers. Commun. Grünwald

Pers. Commun. Zanotelli

Pers. Commun. Ohtsuka

Pers. Commun. Corre

Pers. Commun. York

Pers. Commun. Spotswood

Pers. Commun. Dore

Pers. Commun. Koteen

Pers. Commun. Suykers

Pers. Commun. Dukes

Database published sources

1. Adamek, M., Corre, M. D. & Hölscher, D. Early effect of elevated nitrogen input on above-ground net primary production of a lower montane rain forest, Panama. *J. Trop. Ecol.* **25**, 637–647 (2009).
2. Adamek, M., Corre, M. D. & Hölscher, D. Responses of fine roots to experimental nitrogen addition in a tropical lower montane rain forest, Panama. *J. Trop. Ecol.* **27**, 73–81 (2011).
3. Adhikari, B. S., Rawat, Y. S. & Singh, S. P. Structure and function of high-altitude forests of central Himalaya 1. Dry matter dynamics. *Annals of Botany* **75**, 237–248 (1995).
4. Adzmi, Y. *et al.* Heterogeneity of soil morphology and hydrology on the 50 HA long-term ecological research plot at Pasoh, peninsular Malaysia. *Journal of Tropical Forest Science* (2010).
5. Aerts, R. Nutrient turnover in Dutch heathlands during succession from ericaceous to gramineous dominance. *Scripta Geobot.* **21**, 7–15 (1993).
6. Aerts, R. Aboveground Biomass and Nutrient Dynamics of Calluna vulgaris and Molinia caerulea in a Dry Heathland. *Oikos* **56**, 31 (1989).
7. Aerts, R., Bakker, C. & De Caluwe, H. Root turnover as determinant of the cycling of C,N and P in a dry heathland ecosytem. *Biogeochemistry* **15**, 175–190 (1992).
8. Alberti, G. *et al.* Changes in CO2 emissions after crop conversion from continuous maize to alfalfa. *Agriculture, Ecosystems & Environment* **136**, 139–147 (2010).
9. Allen, A. S. *et al.* Effects of Free-Air CO 2 Enrichment (FACE) on Belowground Processes in a Pinus taeda Forest. *Ecological Applications* **10**, 437 (2000).
10. Almagro, M., López, J., Boix-Fayos, C., Albaladejo, J. & Martínez-Mena, M. Belowground carbon

allocation patterns in a dry Mediterranean ecosystem: A comparison of two models. *Soil Biology and Biochemistry* **42**, 1549–1557 (2010).

11. Almagro, M., Querejeta, J. I., Boix-Fayos, C. & Martínez-Mena, M. Links between vegetation patterns, soil C and N pools and respiration rate under three different land uses in a dry Mediterranean ecosystem. *J Soils Sediments* **13**, 641–653 (2013).

12. Alon, M. & Sternberg, M. Effects of extreme drought on primary production, species composition and species diversity of a Mediterranean annual plant community. *J Vegetation Science* **30**, 1045–1061 (2019).

13. Amaral, J. A. & Knowles, R. Localization of methane consumption and nitrification activities in some boreal forest soils and the stability of methane consumption on storage and disturbance. *J. Geophys. Res.* **102**, 29255–29260 (1997).

14. Anthoni, P. M., Freibauer, A., Kolle, O. & Schulze, E.-D. Winter wheat carbon exchange in Thuringia, Germany. *Agricultural and Forest Meteorology* **121**, 55–67 (2004).

15. Aragão, L. E. O. C. *et al.* Above- and below-ground net primary productivity across ten Amazonian forests on contrasting soils. *Biogeosciences* **6**, 2759–2778 (2009).

16. Araujo-Murakami, A. *et al.* The productivity, allocation and cycling of carbon in forests at the dry margin of the Amazon forest in Bolivia. *Plant Ecology & Diversity* **7**, 55–69 (2014).

17. Aubinet, M. *et al.* Carbon sequestration by a crop over a 4-year sugar beet/winter wheat/seed potato/winter wheat rotation cycle. *Agricultural and Forest Meteorology* **149**, 407–418 (2009).

18. Awasthi, P., Bargali, K., Bargali, S. S. & Jhariya, M. K. Structure and functioning of *Coriaria nepalensis* dominated shrublands in degraded hills of Kumaun Himalaya. I. Dry matter dynamics. *Land Degrad Dev* **33**, 1474–1494 (2022).

19. Bagchi, S., Bhatnagar, Y. V. & Ritchie, M. E. Comparing the effects of livestock and native herbivores on plant production and vegetation composition in the Trans-Himalayas. *Pastor Res Policy Pract* **2**,

21 (2012).

20. Bagchi, S. & Ritchie, M. E. Herbivore effects on above- and belowground plant production and soil nitrogen availability in the Trans-Himalayan shrub-steppes. *Oecologia* **164**, 1075–1082 (2010).

21. Baishya, R. & Barik, S. K. Estimation of tree biomass, carbon pool and net primary production of an old-growth Pinus kesiya Royle ex. Gordon forest in north-eastern India. *Annals of Forest Science* **68**, 727–736 (2011).

22. Bakker, E. S., Knops, J. M. H., Milchunas, D. G., Ritchie, M. E. & Olff, H. Cross-site comparison of herbivore impact on nitrogen availability in grasslands: the role of plant nitrogen concentration. *Oikos* **118**, 1613–1622 (2009).

23. Baptist, F. & Choler, P. A Simulation of the Importance of Length of Growing Season and Canopy Functional Properties on the Seasonal Gross Primary Production of Temperate Alpine Meadows. *Annals of Botany* **101**, 549–559 (2008).

24. Bartsch, N. Element release in Beech (Fagus Sylvatica L.) forest gaps. *Water, Air and Soil Pollution* **122**, 3–16 (1992).

25. Bascietto, M. *et al.* Database of tree stands (Structure, age, biomass, LAI and NPP) of the FORCAST project. (2003).

26. Battles, J. J. *et al.* Net Primary Production and Biomass Distribution in the Blue Oak Savanna. Technical report. (2006).

27. Belelli Marchesini, L. Analysis of the carbon cycle of steppe and old field ecosystems of central Asia. *PhD Thesis*, Università degli studi della Tuscia – Viterbo. (2008).

28. Belelli Marchesini, L. *et al.* Carbon balance assessment of a natural steppe of southern Siberia by multiple constraint approach. *Biogeosciences* **4**, 581–595. (2007).

29. Bergeron, O. *et al.* Comparison of carbon dioxide fluxes over three boreal black spruce forests in Canada. *Global Change Biology* **13**, 89–107 (2007).

30. Bergeron, O., Margolis, H. A., Coursolle, C. & Giasson, M.-A. How does forest harvest influence carbon dioxide fluxes of black spruce ecosystems in eastern North America? *Agricultural and Forest Meteorology* **148**, 537–548 (2008).

31. Berggren Kleja, D. *et al.* Pools and fluxes of carbon in three Norway spruce ecosystems along a climatic gradient in Sweden. *Biogeochemistry* **89**, 7–25 (2008).

32. Bergh, J. *et al.* Modelling the short-term effects of climate change on the productivity of selected tree species in Nordic countries. *Forest Ecology and Management* **183**, 327–340 (2003).

33. Bernard, J. M. Seasonal Changes in Standing Crop and Primary Production in a Sedge Wetland and an Adjacent Dry Old-Field in Central Minnesota. *Ecology* **55**, 350–359 (1974).

34. Bernard, J. M. Nutrient cycling in a Carex-Lacustris wetland. *Canadian Journal of Botany* **55**, 630–638 (1977).

35. Bernard, J. M. & Hankinson, G. Seasonal Changes in Standing Crop, Primary Production, and Nutrient Levels in a Carex rostrata Wetland. *Oikos* **32**, 328 (1979).

36. Bernard, J. M. & Macdonald Jr., J. G. Primary production and life history of *Carex lacustris*. *Can. J. Bot.* **52**, 117–123 (1974).

37. Bernard, J. M., Seischab, F. K. & Jacoby, G. Life History and Production of Above-and Belowground Structures of Cladium mariscoides (Muhl.) Torr. in a Western New York Fen. *Bulletin of the Torrey Botanical Club* **112**, 288 (1985).

38. Bhardwaj, A. K. *et al.* Water and energy footprints of bioenergy crop production on marginal lands: LAND MARGINALITY EFFECTS ON WATER AND ENERGY USE. *GCB Bioenergy* **3**, 208–222 (2011).

39. Biondini, M. E., Patton, B. D. & Nyren, P. E. Grazing intensity and ecosystem processes in a northern mixed-grass prairie, USA. *Ecological Applications* **8**, 469–479 (1998).

40. Bitter, G. Microclimate of alpine grasslands under experimental drought conditions.

41. Black, K. *et al.* Inventory and eddy covariance-based estimates of annual carbon sequestration in a

Sitka spruce (Picea sitchensis (Bong.) Carr.) forest ecosystem. *Eur J Forest Res* **126**, 167–178 (2007).

42. Black, K., Tobin, B., Saiz, G., Byrne, K. A. & Osborne, B. Improved estimates of biomass expansion factors for Sitka spruce. *Irish Forestry* (2004).

43. Black, K. G. & Farrell, E. P. *Carbon Sequestration and Irish Forest Ecosystems*. COFORD, Dublin. (2006).

44. Black, T. A. *et al.* Annual cycles of water vapour and carbon dioxide fluxes in and above a boreal aspen forest. *Global Change Biology* **2**, 219–229 (1996).

45. *Tundra Ecosystems: A Comparative Analysis*. (Cambridge University Press, Cambridge [Eng.] ; New York, 1981).

46. *Truelove Lowland, Devon Island, Canada: A High Arctic Ecosystem*. (Univ. of Alberta Pr, Edmonton, Alberta, 1987).

47. Boegh, E. *et al.* Remote sensing-based evapotranspiration and runoff modelling of agricultural, forest and urban flux sites in Denmark: From field to macro-scale. *Journal of Hydrology* **377**, 300–316 (2009).

48. Bond-Lamberty, B., Wang, C. & Gower, S. T. Annual carbon flux from woody debris for a boreal black spruce fire chronosequence. *J.-Geophys.-Res.* **107**, (2002).

49. Bond-Lamberty, B., Wang, C. & Gower, S. T. Net primary production and net ecosystem production of a boreal black spruce wildfire chronosequence. *Global Change Biology* **10**, 473–487 (2004).

50. Borken, W. & Beese, F. Control of Nitrous Oxide Emissions in European Beech, Norway Spruce and Scots Pine Forests. *Biogeochemistry* **76**, 141–159 (2005).

51. Broeckx, L. S., Verlinden, M. S. & Ceulemans, R. Establishment and two-year growth of a bio-energy plantation with fast-growing Populus trees in Flanders (Belgium): Effects of genotype and former land use. *Biomass and Bioenergy* **42**, 151–163 (2012).

52. Brown, R. F. & Collins, S. L. As above, not so below: Long-term dynamics of net primary production

across a dryland transition zone. *Global Change Biology* **29**, 3941–3953 (2023).

53. Brye, K. R., Andraski, T. W., Jarrell, W. M., Bundy, L. G. & Norman, J. M. Phosphorus Leaching under a Restored Tallgrass Prairie and Corn Agroecosystems. *J. ENVIRON. QUAL.* **31**, (2002).

54. Brye, K. R., Norman, J. M., Gower, S. T. & Bundy, L. G. Effects of management practices on annual net N-mineralization in a restored prairie and maize agroecosystems. *Biogeochemistry* **63**, 135–160 (2003).

55. Brye, K. R., Norman, J. M., Bundy, L. G. & Gower, S. T. Water-Budget Evaluation of Prairie and Maize Ecosystems. *Soil Science Soc of Amer J* **64**, 715–724 (2000).

56. Brye, K. R., Norman, J. M. & Gower, S. T. Assessing the Progress of a Tallgrass Prairie Restoration in Southern Wisconsin. *Am Midl Nat* **148**, 218 (2002).

57. Brye, K. R., Norman, J. M., Gower, S. T. & Bundy, L. G. Methodological limitations and N-budget differences among a restored tallgrass prairie and maize agroecosystems. *Agriculture, Ecosystems & Environment* **97**, 181–198 (2003).

58. Brye, K. R., Gower, S. T., Norman, J. M. & Bundy, L. G. Carbon budgets for a prairie and agroecosystems: effects of land use and interannual variability. *Ecological Applications* **12**, 962–979 (2002).

59. Burke, I. C. & Mosier, A. R. Soil Organic Matter and Nutrient Dynamics of Shortgrass Steppe Ecosystems. in *Ecology of the Shortgrass Steppe* (Oxford University Press, 2008). doi:10.1093/oso/9780195135824.003.0017.

60. Butt, N. *et al*. Initial Results from Establishment of a Long-Term Broadleaf Monitoring Plot at Wytham Woods, Oxford, UK. 83 (2009).

61. Cabido, M., Breimer, R. & Vega, G. Plant communities and associated soil types in a high plateau of the Cordoba mountains, central Argentina. *Mountain Res. Dev.* **7**, 25–42 (1987).

62. Cahill, K. N., Kucharic, C. J. & work(s):, J. A. F. R. Prairie Restoration and Carbon Sequestration:

Difficulties Quantifying C Sources and Sinks Using a Biometric Approach. *Ecological Applications* **19**, 2185–2201 (2009).

63. Caldwell, M. M. & Camp, L. B. Belowground productivity of two cool desert communities. *Oecologia* **17**, 123–130 (1974).

64. Caldwell, M. M., White, R. S., Moore, R. T. & Camp, L. B. Carbon balance, productivity, and water use of cold-winter desert shrub communities dominated by C3 and C4 species. *Oecologia* **29**, 275–300 (1977).

65. Camill, P. *et al.* Community- and Ecosystem-Level Changes in a Species-Rich Tallgrass Prairie Restoration. *Ecological Applications* **14**, 1680–1694 (2004).

66. Campbell, J., Alberti, G., Martin, J. & Law, B. E. Carbon dynamics of a ponderosa pine plantation following a thinning treatment in the northern Sierra Nevada. *Forest Ecology and Management* **257**, 453–463 (2009).

67. Campioli, M. *et al.* Evaluating the convergence between eddy-covariance and biometric methods for assessing carbon budgets of forests. *Nat Commun* **7**, 13717 (2016).

68. Campioli, M. *et al.* Biomass production efficiency controlled by management in temperate and boreal ecosystems. *Nature Geosci* **8**, 843–846 (2015).

69. Campioli, M. *et al.* Net Primary Production and Carbon Stocks for Subarctic Mesic–Dry Tundras with Contrasting Microtopography, Altitude, and Dominant Species. *Ecosystems* **12**, 760–776 (2009).

70. Campo, J., Maass, J. M., Jaramillo, V. J. & Yrízar, A. M. Calcium, potassium, and magnesium cycling in a Mexican tropical dry forest ecosystem. *Biogeochemistry* **49**, 21–36 (2000).

71. Campo, J., Maass, M., Jaramillo, V. J., Martínez-Yrízar, A. & Sarukhán, J. Phosphorus cycling in a Mexican tropical dry forest ecosystem. Biogeochemistry **53**, 161–179 (2001).

72. Caquet, B. *et al.* Soil carbon balance in a tropical grassland: Estimation of soil respiration and its partitioning using a semi-empirical model. *Agricultural and Forest Meteorology* **158–159**, 71–79

(2012).

73. Ceulemans, R. *et al.* Coniferous Forests (Scots and Maritime Pine): Carbon and Water Fluxes, Balances, Ecological and Ecophysiological Determinants. in *Fluxes of Carbon, Water and Energy of European Forests* (ed. Valentini, R.) vol. 163 71–97 (Springer Berlin Heidelberg, Berlin, Heidelberg, 2003).

74. Chambers, J. Q. *et al.* Respiration from a tropical forest ecosystem: partitioning of sources and low carbon use efficiency. *Ecological Applications* **14**, 72–88 (2004).

75. Chapin, F. S., Shaver, G. R., Giblin, A. E., Nadelhoffer, K. J. & Laundre, J. A. Responses of Arctic Tundra to Experimental and Observed Changes in Climate. *Ecology* **76**, 694–711 (1995).

76. Chen, D. *et al.* Subtropical plantations are large carbon sinks: Evidence from two monoculture plantations in South China. *Agricultural and Forest Meteorology* **151**, 1214–1225 (2011).

77. Chew, R. M. & Chew, A. E. The Primary Productivity of a Desert-Shrub (Larrea tridentata) Community. *Ecological Monographs* **35**, 355–375 (1965).

78. Chimner, R. A. & Cooper, D. J. Carbon dynamics of pristine and hydrologically modified fens in the southern Rocky Mountains. *Can. J. Bot.* **81**, 477–491 (2003).

79. Chou, W. W., Silver, W. L., Jackson, R. D., Thompson, A. W. & Allen-Diaz, B. The sensitivity of annual grassland carbon cycling to the quantity and timing of rainfall. *Global Change Biology* **14**, 1382–1394 (2008).

80. Clark, D. A. *et al.* Net primary production in tropical forests: an evaluation and synthesis of existing field data. *Ecological Applications* **11**, 371–384 (2001).

81. Clark, M. R., Coupe, M. D., Bork, E. W. & Cahill, J. F. Interactive effects of insects and ungulates on root growth in a native grassland. *Oikos* **121**, 1585–1592 (2012).

82. Cook, B. D. *et al.* Carbon exchange and venting anomalies in an upland deciduous forest in northern Wisconsin, USA. *Agricultural and Forest Meteorology* **126**, 271–295 (2004).

83. Corney, P. M. *et al.* Changes in the field-layer of Wytham Woods - assessment of the impacts of a range of environmental factors controlling change. *J Vegetation Science* **19**, 287–298 (2008).

84. Corre, M. D., Veldkamp, E., Arnold, J. & Wright, S. J. Impact of elevated N input on soil N cycling and losses in old-growth lowland and montane forests in Panama. *Ecology* **91**, 1715–1729 (2010).

85. Couto, E. G. & Oliveira, V. Á. The Soil Diversity of the Pantanal. in *The Pantanal: Ecology, biodiversity and sustainable management of a large neotropical seasonal wetland* 71–102 (Pensoft Publishers, Sofia–Moscow, 2010).

86. Crews, T. E. *et al.* Changes in Soil Phosphorus Fractions and Ecosystem Dynamics across a Long Chronosequence in Hawaii. *Ecology* **76**, 1407–1424 (1995).

87. Curtis, P. S. *et al.* Biometric and eddy-covariance based estimates of annual carbon storage in five eastern North American deciduous forests. *Agricultural and Forest Meteorology* **113**, 3–19 (2002).

88. Da Rocha, H. R. *et al.* Seasonality of water and heat fluxes over a tropical forest in eastern Amazonia. *Ecological Applications* **14**, 22–32 (2004).

89. Dahlgren, R. A., Singer, M. J. & Huang, X. Oak tree and grazing impacts on soil properties and nutrients in a California oak woodland. *Biogeochemistry* **39**, 45–64 (1997).

90. Dahlgren, R. A., Horwath, W. R., Tate, K. W. & Camping, T. J. Blue oak enhance soil quality in California oak woodlands. *Cal Ag* **57**, 42–47 (2003).

91. Davis, R. C. Structure and Function of Two Antarctic Terrestrial Moss Communities. *Ecological Monographs* **51**, 125–143 (1981).

92. de Grandcourt, A. *et al. Vegetation Dynamics in a Littoral Savannah in Congo*. (2008).

93. Deal, M. W. *et al.* Net primary production in three bioenergy crop systems following land conversion. *Journal of Plant Ecology* **7**, 451–460 (2014).

94. DeAngelis, D. L., Gardner, R. H. & Shugart, H. H. Productivity of forest ecosystems studied during the IBP: the woodlands data set. in *Dynamic properties of forest ecosystems* (ed. Reichle, D. E.)

567–672 (Cambridge University Press, Cambridge, UK, 1981).

95. del Aguila-Pasquel, J. *et al.* The seasonal cycle of productivity, metabolism and carbon dynamics in a wet aseasonal forest in north-west Amazonia (Iquitos, Peru). *Plant Ecology & Diversity* **7**, 71–83 (2014).

96. DeLucia, E. H. *et al.* Net Primary Production of a Forest Ecosystem with Experimental CO_2 Enrichment. *Science* **284**, 1177–1179 (1999).

97. Deutsch, E. S., Bork, E. W. & Willms, W. D. Soil moisture and plant growth responses to litter and defoliation impacts in Parkland grasslands. *Agriculture, Ecosystems & Environment* **135**, 1–9 (2010).

98. Diemer, M. & Korner, C. Transient Enhancement of Carbon Uptake in an Alpine Grassland Ecosystem under Elevated CO 2. *Arctic and Alpine Research* **30**, 381 (1998).

99. Dilly, O. *et al.* Characteristics and energetic strategies of the rhizosphere in ecosystems of the Bornhöved Lake district. *Applied Soil Ecology* **15**, 201–210 (2000).

100. Doll, J. E., Brink, G. E., Cates, R. L. & Jackson, R. D. Effects of Native Grass Restoration Management on Above- and Belowground Pasture Production and Forage Quality. *Journal of Sustainable Agriculture* **33**, 512–527 (2009).

101. Dore, S. *et al.* Carbon and water fluxes from ponderosa pine forests disturbed by wildfire and thinning. *Ecological Applications* **20**, 663–683 (2010).

102. Dufranne, D., Moureaux, C., Vancutsem, F., Bodson, B. & Aubinet, M. Comparison of carbon fluxes, growth and productivity of a winter wheat crop in three contrasting growing seasons. *Agriculture, Ecosystems & Environment* **141**, 133–142 (2011).

103. Dufrêne, E. *et al.* Modelling carbon and water cycles in a beech forest. *Ecological Modelling* **185**, 407–436 (2005).

104. Dugas, W. A., Heuer, M. L. & Mayeux, H. S. Carbon dioxide fluxes over bermudagrass, native prairie, and sorghum. *Agricultural and Forest Meteorology* (1999).

105. Ehman, J. L. *et al.* An initial intercomparison of micrometeorological and ecological inventory estimates of carbon exchange in a mid-latitude deciduous forest. *Global Change Biology* **8**, 575–589 (2002).

106. Epron, D. *et al.* Partitioning of net primary production in Eucalyptus and Acacia stands and in mixed-species plantations: Two case-studies in contrasting tropical environments. *Forest Ecology and Management* **301**, 102–111 (2013).

107. Esser, G., *et al*. The high resolution biosphere model : status of development, validation, results. / Le modèle biosphère haute résolution (HRBM) : état des travaux, validation, résultats. *sgeol* **50**, 73–88 (1997).

108. Etzold, S. *et al.* The Carbon Balance of Two Contrasting Mountain Forest Ecosystems in Switzerland: Similar Annual Trends, but Seasonal Differences. *Ecosystems* **14**, 1289–1309 (2011).

109. Fang, Q. *et al.* Soil nitrate accumulation, leaching and crop nitrogen use as influenced by fertilization and irrigation in an intensive wheat–maize double cropping system in the North China Plain. *Plant Soil* **284**, 335–350 (2006).

110. Faqi, W., Haibin, L., Baosheng, S., Jian, W. & Gale, W. J. Net primary production and nutrient cycling in an apple orchard–annual crop system in the Loess Plateau, China: a comparison of Qinguan apple, Fuji apple, corn and millet production subsystems. *Nutr Cycl Agroecosyst* **81**, 95–105 (2008).

111. Fenn, K., Malhi, Y., Morecroft, M., Lloyd, C. & Thomas, M. Comprehensive Description of the Carbon Cycle of an Ancient Temperate Broadleaved Woodland. https://bg.copernicus.org/preprints/7/3735/2010/ (2010) doi:10.5194/bgd-7-3735-2010.

112. Fernández-Martínez, M. *et al.* Nutrient availability as the key regulator of global forest carbon balance. *Nature Clim Change* **4**, 471–476 (2014).

113. Fiala, K. Belowground plant biomass of grassland ecosystems and its variation according to ecological factors. *ekol* **29**, 182–206 (2010).

114. Fisk, M. C., Schmidt, S. K. & Seastedt, T. R. Topographic patterns of above- and belowground production and nitrogen cycling in apline tundra. *Ecology* **79**, 2253–2266 (1998).

115. Flanagan, L. B., Wever, L. A. & Carlson, P. J. Seasonal and interannual variation in carbon dioxide exchange and carbon balance in a northern temperate grassland. *Global Change Biology* **8**, 599–615 (2002).

116. Forrest, G. I. Structure and Production of North Pennine Blanket Bog Vegetation. *The Journal of Ecology* **59**, 453 (1971).

117. Forrest, G. I. Structure and Production of North Pennine Blanket Bog Vegetation. *The Journal of Ecology* **59**, 453 (1971).

118. Forrest, G. I. & Smith, R. A. H. The Productivity of a Range of Blanket Bog Vegetation Types in the Northern Pennines. *The Journal of Ecology* **63**, 173 (1975).

119. Fukuzawa, K. *et al.* Temporal variation in fine-root biomass, production and mortality in a cool temperate forest covered with dense understory vegetation in northern Japan. *Forest Ecology and Management* **310**, 700–710 (2013).

120. Gao, Y. Z., Chen, Q., Lin, S., Giese, M. & Brueck, H. Resource manipulation effects on net primary production, biomass allocation and rain-use efficiency of two semiarid grassland sites in Inner Mongolia, China. *Oecologia* **165**, 855–864 (2011).

121. Gao, Y. Z. *et al.* Belowground net primary productivity and biomass allocation of a grassland in Inner Mongolia is affected by grazing intensity. *Plant Soil* **307**, 41–50 (2008).

122. Garcia-Moya, E. NPP Grassland: Montecillo, Mexico, 1984-1994, R1. 0.021576 MB (1999) doi:10.3334/ORNLDAAC/413.

123. Garkoti, S. C. Estimates of biomass and primary productivity in a high-altitude maple forest of the west central Himalayas. *Ecological Research* **23**, 41–49 (2008).

124. Garkoti, S. C. & Singh, S. P. Variation in net primary productivity and biomass of forests in the high

mountains of Central Himalaya. *J Vegetation Science* **6**, 23–28 (1995).

125. Gates, D. H., Stoddart, L. A. & Cook, C. W. Soil as a Factor Influencing Plant Distribution on Salt-Deserts of Utah. *Ecological Monographs* **26**, 155–175 (1956).

126. Gaudinski, J. B., Trumbore, S. E., Davidson, E. A. & Zheng, S. Soil carbon cycling in a temperate forest: radiocarbon-based estimates of residence times, sequestration rates and partitioning of fluxes. *Biogeochemistry* **51**, 53–69 (2000).

127. Giardina, C. P. & Ryan, M. G. Total Belowground Carbon Allocation in a Fast-growing Eucalyptus Plantation Estimated Using a Carbon Balance Approach. *Ecosystems* **5**, 487–499 (2002).

128. Giardina, C. P., Ryan, M. G., Binkley, D. & Fownes, J. H. Primary production and carbon allocation in relation to nutrient supply in a tropical experimental forest. *Global Change Biology* **9**, 1438–1450 (2003).

129. Gielen, B. *et al.* Net carbon storage in a poplar plantation (POPFACE) after three years of free-air CO2 enrichment. *Tree Physiology* **25**, 1399–1408 (2005).

130. Gilmanov, T. G. *et al.* Productivity, Respiration, and Light-Response Parameters of World Grassland and Agroecosystems Derived From Flux-Tower Measurements. *Rangeland Ecology & Management* **63**, 16–39 (2010).

131. Gilmanov, T. G., Parton, W. J. & Ojima, D. S. Testing the 'CENTURY' ecosystem level model on data sets from eight grassland sites in the former USSR representing a wide climatic/soil gradient. *Ecological Modelling* **96**, 191–210 (1997).

132. Gilmanov, T. G. *et al.* Gross primary production and light response parameters of four Southern Plains ecosystems estimated using long-term CO_2 -flux tower measurements. *Global Biogeochemical Cycles* **17**, 2002GB002023 (2003).

133. Girardin, C. A. J. *et al.* Fine root dynamics along an elevational gradient in tropical Amazonian and Andean forests. *Global Biogeochemical Cycles* **27**, 252–264 (2013).

134. Girardin, C. A. J. *et al.* Net primary productivity allocation and cycling of carbon along a tropical forest elevational transect in the Peruvian Andes. *Global Change Biology* **16**, 3176–3192 (2010).

135. Girardin, C. A. J. *et al.* Productivity and carbon allocation in a tropical montane cloud forest in the Peruvian Andes. *Plant Ecology & Diversity* **7**, 107–123 (2014).

136. Golovatskaya, E. A. & Dyukarev, E. A. Carbon budget of oligotrophic mire sites in the Southern Taiga of Western Siberia. *Plant Soil* **315**, 19–34 (2009).

137. *Freshwater Wetlands: Ecological Processes and Management Potential*. (Academic Press, New York San Francisco London, 1978).

138. Gopal, B. Bulletin of the National Institute of Ecology 16: 00-00, 2005.

139. Gough, C. M., Vogel, C. S., Harrold, K. H., George, K. & Curtis, P. S. The legacy of harvest and fire on ecosystem carbon storage in a north temperate forest. *Global Change Biology* **13**, 1935–1949 (2007).

140. Goulden, M. L. *et al.* Diel and seasonal patterns of tropical forest for CO_2 exchange. *Ecological Applications* **14**, 42–54 (2004).

141. Gower, S. T. *et al.* Net primary production and carbon allocation patterns of boreal forest ecosystems. *Ecological Applications* **11**, 1395–1411 (2001).

142. Gower, S. T. *et al.* Carbon distribution and aboveground net primary production in aspen, jack pine, and black spruce stands in Saskatchewan and Manitoba, Canada. *J. Geophys. Res.* **102**, 29029–29041 (1997).

143. Gower, S. *et al.* Nutrient dynamics of the southern and northern BOREAS boreal forests. *Écoscience* **7**, 481–490 (2000).

144. Grady, K. C. & Hart, S. C. Influences of thinning, prescribed burning, and wildfire on soil processes and properties in southwestern ponderosa pine forests: A retrospective study. *Forest Ecology and Management* **234**, 123–135 (2006).

145. Granier, A., Bréda, N., Longdoz, B., Gross, P. & Ngao, J. Ten years of fluxes and stand growth in a young beech forest at Hesse, North-eastern France. *Ann. For. Sci.* **65**, 704–704 (2008).

146. Grant, R. F. *et al.* Net ecosystem productivity of boreal jack pine stands regenerating from clearcutting under current and future climates. *Global Change Biology* **13**, 1423–1440 (2007).

147. Grant, R. F., Desai, A. R. & Sulman, B. N. Modelling contrasting responses of wetland productivity to changes in water table depth. *Biogeosciences* **9**, 4215–4231 (2012).

148. Grant, R. F. & Flanagan, L. B. Modeling stomatal and nonstomatal effects of water deficits on CO_2 fixation in a semiarid grassland. *J. Geophys. Res.* **112**, 2006JG000302 (2007).

149. Grant, R. F., Oechel, W. C. & Ping, C. Modelling carbon balances of coastal arctic tundra under changing climate. *Global Change Biology* **9**, 16–36 (2003).

150. Griffis, T. J. *et al.* Seasonal variation and partitioning of ecosystem respiration in a southern boreal aspen forest. *Agricultural and Forest Meteorology* **125**, 207–223 (2004).

151. Guckland, A. Nutrient stocks, acidity, processes of N transformation and net uptake of methane in soils of a temperate deciduous forest with different abundance of beech (Fagus sylvatica L.). (Georg-August-University Göttingen, 2009). doi:10.53846/goediss-2298.

152. Guckland, A., Jacob, M., Flessa, H., Thomas, F. M. & Leuschner, C. Acidity, nutrient stocks, and organic-matter content in soils of a temperate deciduous forest with different abundance of European beech (*Fagus sylvatica* L.). *Z. Pflanzenernähr. Bodenk.* **172**, 500–511 (2009).

153. Guedes, B. S., Olsson, B. A., Sitoe, A. A. & Egnell, G. Net primary production in plantations of Pinus taeda and Eucalyptus cloeziana compared with a mountain miombo woodland in Mozambique. *Global Ecology and Conservation* **15**, e00414 (2018).

154. Guo, L. B., Cowie, A. L., Montagu, K. D. & Gifford, R. M. Carbon and nitrogen stocks in a native pasture and an adjacent 16-year-old Pinus radiata D. Don. plantation in Australia. *Agriculture, Ecosystems & Environment* **124**, 205–218 (2008).

155. Gupta, R. K. & Ratan, N. Biomass dynamics, net primary production and turnover rate of grassland community in Bundelkhand region (U.P.). *Bulletin of the National Institute of Ecology* **16**, 87–94 (2005).

156. Gupta, S. R. & Singh, J. S. Soil respiration in a tropical grassland. *Soil Biology and Biochemistry* **13**, 261–268 (1981).

157. Hamilton, J. G. *et al.* Forest carbon balance under elevated CO2. *Oecologia* **131**, 250–260 (2002).

158. Han, G. H. *et al.* Isotopic disequilibrium between carbon assimilated and respired in a rice paddy as influenced by methanogenesis from CO_2. *J. Geophys. Res.* **112**, 2006JG000219 (2007).

159. Han, G. *et al.* Soil temperature and biotic factors drive the seasonal variation of soil respiration in a maize (Zea mays L.) agricultural ecosystem. *Plant Soil* **291**, 15–26 (2007).

160. Hanson, P. J., Edwards, N. T., Tschaplinski, T. J., Wullschleger, S. D. & Joslin, J. D. Estimating the net primary and net ecosystem production of a southeastern upland Quercus forest from an 8-year biometric record. in *North american temperate deciduous forest responses to changing precipitation regimes.* (Springer New York, 2004).

161. *North American Temperate Deciduous Forest Responses to Changing Precipitation Regimes*. (Springer, New York, 2003).

162. Harmon, M. E. *et al.* Production, Respiration, and Overall Carbon Balance in an Old-growth Pseudotsuga-Tsuga Forest Ecosystem. *Ecosystems* (2004) doi:10.1007/s10021-004-0140-9.

163. Hartley, I. P., Hopkins, D. W., Sommerkorn, M. & Wookey, P. A. The response of organic matter mineralisation to nutrient and substrate additions in sub-arctic soils. *Soil Biology and Biochemistry* **42**, 92–100 (2010).

164. Hayes, D. C. & Seastedt, T. R. Root dynamics of tallgrass prairie in wet and dry years. *Can. J. Bot.* **65**, 787–791 (1987).

165. Heikkinen, J. E. P., Elsakov, V. & Martikainen, P. J. Carbon dioxide and methane dynamics and

annual carbon balance in tundra wetland in NE Europe, Russia. *Global Biogeochemical Cycles* **16**, (2002).

166. Heikkinen, J. E. P., Virtanen, T., Huttunen, J. T., Elsakov, V. & Martikainen, P. J. Carbon balance in East European tundra. *Global Biogeochemical Cycles* **18**, 2003GB002054 (2004).

167. Henry, G. H. R., Svoboda, J. & Freedman, B. Standing crop and net production of sedge meadows of an ungrazed polar desert oasis. *Canadian Journal of Botany* **68**, (1990).

168. Herbert, D. A. & Fownes, J. H. Phosphorus Limitation of Forest Leaf Area and Net Primary Production on a Highly Weathered Soil. *Biogeochemistry* **29**, 223–235 (1995).

169. Herbert, D. A. & Fownes, J. H. Forest Productivity and Efficiency of Resource Use Across a Chronosequence of Tropical Montane Soils. *Ecosystems* **2**, 242–254 (1999).

170. Hermle, S., Lavigne, M. B., Bernier, P. Y., Bergeron, O. & Pare, D. Component respiration, ecosystem respiration and net primary production of a mature black spruce forest in northern Quebec. *Tree Physiology* **30**, 527–540 (2010).

171. Hertel, D. *et al.* Below- and above-ground biomass and net primary production in a paleotropical natural forest (Sulawesi, Indonesia) as compared to neotropical forests. *Forest Ecology and Management* **258**, 1904–1912 (2009).

172. Hertel, D. & Leuschner, C. A comparison of four different fine root production estimates with ecosystem carbon balance data in a Fagus–Quercus mixed forest.

173. Hibbard, K. A., Law, B. E., Reichstein, M. & Sulzman, J. An analysis of soil respiration across northern hemisphere temperate ecosystems. *Biogeochemistry* **73**, 29–70 (2005).

174. Hicks Pries, C. E., Schuur, E. A. G. & Crummer, K. G. Thawing permafrost increases old soil and autotrophic respiration in tundra: Partitioning ecosystem respiration using δ^{13}C and Δ^{14}C. *Global Change Biology* **19**, 649–661 (2013).

175. Higgins, P. A. T., Jackson, R. B., Des Rosiers, J. M. & Field, C. B. Root production and demography in

a california annual grassland under elevated atmospheric carbon dioxide. *Global Change Biology* **8**, 841–850 (2002).

176. Hirayama, K. & Sakimoto, M. Spatial distribution of canopy and subcanopy species along a sloping topography in a cool-temperate conifer-hardwood forest in the snowy region of Japan. *Ecological Research* **18**, 443–454 (2003).

177. Hoeppner, S. S. & Dukes, J. S. Interactive responses of old-field plant growth and composition to warming and precipitation. *Global Change Biology* **18**, 1754–1768 (2012).

178. Högberg, P., Nordgren, A. & Ågren, G. I. Carbon allocation between tree root growth and root respiration in boreal pine forest. *Oecologia* **132**, 579–581 (2002).

179. Hoosbeek, M. R. *et al.* More new carbon in the mineral soil of a poplar plantation under Free Air Carbon Enrichment (POPFACE): Cause of increased priming effect? *Global Biogeochemical Cycles* **18**, 2003GB002127 (2004).

180. Howard, E. A., Gower, S. T., Foley, J. A. & Kucharik, C. J. Effects of logging on carbon dynamics of a jack pine forest in Saskatchewan, Canada. *Global Change Biology* **10**, 1267–1284 (2004).

181. Hu, R., Hatano, R., Kusa, K. & Sawamoto, T. Effect of nitrogen fertilization on methane flux in a structured clay soil cultivated with onion in Central Hokkaido, Japan. *Soil Science and Plant Nutrition* **48**, 797–804 (2002).

182. Hu, R., Hatano, R., Kusa, K. & Sawamoto, T. Soil respiration and net ecosystem production in an onion field in Central Hokkaido, Japan. *Soil Science and Plant Nutrition* **50**, 27–33 (2004).

183. Huasco, W. H. *et al.* Seasonal production, allocation and cycling of carbon in two mid-elevation tropical montane forest plots in the Peruvian Andes. *Plant Ecology & Diversity* **7**, 125–142 (2014).

184. Hui, D. & Jackson, R. B. Geographical and interannual variability in biomass partitioning in grassland ecosystems: a synthesis of field data. *New Phytologist* **169**, 85–93 (2006).

185. Hunt, H. W. *et al.* Simulation model for the effects of climate change on temperate grassland

ecosystems. *Ecological Modelling* **53**, 205–246 (1991).

186. Hussain, M. Z. *et al.* Summer drought influence on CO2 and water fluxes of extensively managed grassland in Germany. *Agriculture, Ecosystems & Environment* **141**, 67–76 (2011).

187. Illeris, L. *et al.* Growing-Season Carbon Dioxide Flux in a Dry Subarctic Heath: Responses to Long-term Manipulations. *Arctic, Antarctic, and Alpine Research* **36**, 456–463 (2004).

188. Inatomi, M., Ito, A., Ishijima, K. & Murayama, S. Greenhouse Gas Budget of a Cool-Temperate Deciduous Broad-Leaved Forest in Japan Estimated Using a Process-Based Model. *Ecosystems* **13**, 472–483 (2010).

189. Iost, S. Soil respiration, microbial respiration and mineralisation in soils of montane rainforests of Southern Ecuador: influence of altitude. Phd-thesis. (Technischen Universitat Dresden, 2007).

190. Ito, A. & Oikawa, T. A simulation model of the carbon cycle in land ecosystems (Sim-CYCLE): a description based on dry-matter production theory and plot-scale validation. *Ecological Modelling* **151**, 143–176 (2002).

191. Iversen, C. M., Bridgham, S. D. & Kellogg, L. E. Scaling plant nitrogen use and uptake efficiencies in response to nutrient addition in peatlands. *Ecology* **91**, 693–707 (2010).

192. Jackson, L. E., Strauss, R. B., Firestone, M. K. & Bartolome, J. W. Plant and soil nitrogen dynamics in California annual grassland. *Plant Soil* **110**, 9–17 (1988).

193. Janssens, I. A. *et al.* Above- and belowground phytomass and carbon storage in a Belgian Scots pine stand. *Ann. For. Sci.* **56**, 81–90 (1999).

194. Janssens, I. A., Crookshanks, M., Taylor, G. & Ceulemans, R. Elevated atmospheric CO_2 increases fine root production, respiration, rhizosphere respiration and soil CO_2 efflux in Scots pine seedlings. *Global Change Biology* **4**, 871–878 (1998).

195. Jarvis, P. & Linder, S. Constraints to growth of boreal forests. *Nature* **405**, 904–905 (2000).

196. Jenkinson, D. S. *et al.* Estimating net primary production from measurements made on soil organic

matter. **80**, (1999).

197. Jenkinson, D. S., Harkness, D. D., Vance, E. D., Adams, D. E. & Harrison, A. F. Calculating net primary production and annual input of organic matter to soil from the amount and radiocarbon content of soil organic matter. *Soil Biology and Biochemistry* **24**, 295–308 (1992).

198. Jia, S. & Akiyama, T. A precise, unified method for estimating carbon storage in cool-temperate deciduous forest ecosystems. *Agricultural and Forest Meteorology* **134**, 70–80 (2005).

199. Jiang, C. *et al.* Nutrient resorption of coexistence species in alpine meadow of the Qinghai-Tibetan Plateau explains plant adaptation to nutrient-poor environment. *Ecological Engineering* **44**, 1–9 (2012).

200. Jimenez, E. M. *et al*. Fine root dynamics for forests on contrasting soils in the Colombian Amazon. Biogeosciences **6**, 2809–2827. (2009).

201. Jimenez, K. L. *et al.* Carbon dioxide exchange rates from short- and long-hydroperiod Everglades freshwater marsh. *J. Geophys. Res.* **117**, 2012JG002117 (2012).

202. Jin, Z., Dong, Y. S., Qi, Y. C. & An, Z. S. Soil respiration and net primary productivity in perennial grass and desert shrub ecosystems at the Ordos Plateau of Inner Mongolia, China. *Journal of Arid Environments* **74**, 1248–1256 (2010).

203. Johnson, D. W., Cole, D. W., Horng, F. W., van Miegroet, H. & Todd, D. E. *Chemical Characteristics of Two Forested Ultisols and Two Forested Inceptisols Relevant to Anion Production and Mobility*. ORNL/TM-7646, 814591 http://www.osti.gov/servlets/purl/814591/ (1981) doi:10.2172/814591.

204. Johnson, D. W. & Hook, R. I. *Analysis of Biogeochemical Cycling Processes in Walker Branch Watershed*. (Springer New York, New York, NY, 1989).

205. Jordan, C. F. & Escalante, G. Root Productivity in an Amazonian Rain Forest. *Ecology* **61**, 14–18 (1980).

206. Jordan, C., Cuevas, E. & Medina, E. NPP Tropical Forest. Oak Ridge National Laboratory Distributed

Active Archive Center, Oak Ridge, Tennessee, U.S.A. (1999).

207. Jourdan, C. *et al.* Fine root production and turnover in Brazilian Eucalyptus plantations under contrasting nitrogen fertilization regimes. *Forest Ecology and Management* **256**, 396–404 (2008).

208. Kalyn, A. L. & Van Rees, K. C. J. Contribution of fine roots to ecosystem biomass and net primary production in black spruce, aspen, and jack pine forests in Saskatchewan. *Agricultural and Forest Meteorology* **140**, 236–243 (2006).

209. Kamnalrut, A. NPP Grassland: Klong Hoi Khong, Thailand, 1984-1990, R1. 0.009 MB (1996) doi:10.3334/ORNLDAAC/147.

210. Káplová, M., Edwards, K. R. & Květ, J. The effect of nutrient level on plant structure and production in a wet grassland: a field study. *Plant Ecol* **212**, 809–819 (2011).

211. Keith, H. *et al.* Multiple measurements constrain estimates of net carbon exchange by a Eucalyptus forest. *Agricultural and Forest Meteorology* **149**, 535–558 (2009).

212. Kerr, A. C. Soil nitrogen dynamics under simulated global changes in a California annual grassland. (Stanford University, 2002).

213. Keuper, F. *et al.* A frozen feast: thawing permafrost increases plant-available nitrogen in subarctic peatlands. *Global Change Biology* **18**, 1998–2007 (2012).

214. Khan, D., Faheemuddin, M., Shaukat, S. S. & Alam, M. M. Seasonal variation in structure, composition, phytomass, and net primary productivity in a Lasiurus scindicus Henr., and Cenchrus setigerus Vahl., dominated dry sandy desert site of Karachi. *Pakistan Journal of Botany* **32**, 171–210 (2000).

215. Kim, H. Y. *et al*. Growth and nitrogen uptake of CO2-enriched rice under field conditions. *New Phytologist* **150**: 223–229 (2001).

216. Kim, J. & Verma, S. B. Carbon dioxide exchange in a temperate grassland ecosystem. *Boundary-Layer Meteorol* **52**, 135–149 (1990).

217. Kim, J., Verma, S. B. & Clement, R. J. Carbon dioxide budget in a temperate grassland ecosystem. *J. Geophys. Res.* **97**, 6057–6063 (1992).

218. Kim, J., Verma, S. B. & Clement, R. J. Carbon dioxide budget in a temperate grassland ecosystem. *J. Geophys. Res.* **97**, 6057–6063 (1992).

219. Kimball, J. S., Thornton, P. E., White, M. A. & Running, S. W. Simulating forest productivity and surface-atmosphere carbon exchange in the BOREAS study region. *Tree Physiology* **17**, 589–599 (1997).

220. Kinyamario, J. I. NPP Grassland: Nairobi, Kenya, 1984-1994, R1. 0.015621 MB (1996) doi:10.3334/ORNLDAAC/151.

221. Kinyamario, J. I. ORNL DAAC NPP grassland: Nairobi, Kenya, 1984-1994. http://www.daac.ornl.gov (1996).

222. Kira, T., Manokaran, N. & Appanah, S. NPP Tropical Forest. Oak Ridge National Laboratory Distributed Active Archive Center, Oak Ridge, Tennessee, U.S.A. (1998).

223. Kjelvik, S. & Kärenlampi, L. Plant biomass and primary production of Fennoscandian subarctic and subalpine forests and of alpine willow and heath ecosystems. in *Fennoscandian Tundra Ecosystems. Part 1: Plants and Microorganisms* vol. 16 366 (Springer-Verlag, 1975).

224. Knapp, A. K., Conard, S. L. & Blair, J. M. Determinants of Soil CO 2 Flux from a Sub-Humid Grassland: Effect of Fire and Fire History. *Ecological Applications* **8**, 760 (1998).

225. Knohl, A., Schulze, E.-D., Kolle, O. & Buchmann, N. Large carbon uptake by an unmanaged 250-year-old deciduous forest in Central Germany. *Agricultural and Forest Meteorology* **118**, 151–167 (2003).

226. Koizumi, H., Usami, Y. & Satoh, M. Annual net primary production and efficiency of solar energy utilization in three double-cropping agro-ecosystems in Japan. *Agriculture, Ecosystems & Environment* **32**, 241–255 (1990).

227. Koizumi, H., Usami, Y. & Satoh, M. Carbon dynamics and budgets in three upland double-cropping agro-ecosystems in Japan. *Agriculture, Ecosystems & Environment* **43**, 235–244 (1993).

228. Konôpka, B., Pajtík, J., Noguchi, K. & Lukac, M. Replacing Norway spruce with European beech: A comparison of biomass and net primary production patterns in young stands. *Forest Ecology and Management* **302**, 185–192 (2013).

229. Konôpka, B., Yuste, J. C., Janssens, I. A. & Ceulemans, R. Comparison of Fine Root Dynamics in Scots Pine and Pedunculate Oak in Sandy Soil. *Plant Soil* **276**, 33–45 (2005).

230. Korrensalo, A. *et al.* Boreal bog plant communities along a water table gradient differ in their standing biomass but not their biomass production. *J Vegetation Science* **29**, 136–146 (2018).

231. Kosykh, N. P. *et al.* The Bogs in a Forest–Steppe Region of Western Siberia: Plant Biomass and Net Primary Production (NPP). *Water* **15**, 3526 (2023).

232. Koteen, L. E., Baldocchi, D. D. & Harte, J. Invasion of non-native grasses causes a drop in soil carbon storage in California grasslands. *Environ. Res. Lett.* **6**, 044001 (2011).

233. Kotowska, M. M., Leuschner, C., Triadiati, T., Meriem, S. & Hertel, D. Quantifying above- and belowground biomass carbon loss with forest conversion in tropical lowlands of S umatra (I ndonesia). *Global Change Biology* **21**, 3620–3634 (2015).

234. Kutsch, W. L., Staack, A., Wötzel, J., Middelhoff, U. & Kappen, L. Field measurements of root respiration and total soil respiration in an alder forest. *New Phytologist* **150**, 157–168 (2001).

235. Laclau, J.-P. *et al.* Biogeochemical cycles of nutrients in tropical Eucalyptus plantations. *Forest Ecology and Management* **259**, 1771–1785 (2010).

236. Laclau, J.-P., Sama-Poumba, W., Nzila, J. D. D., Bouillet, J.-P. & Ranger, J. Biomass and nutrient dynamics in a littoral savanna subjected to annual fires in Congo. *Acta Oecologica* **23**, 41–50 (2002).

237. Ladwig, L. M. *et al.* Above- and belowground responses to nitrogen addition in a Chihuahuan Desert grassland. *Oecologia* **169**, 177–185 (2012).

238. Lafleur, P. M., Roulet, N. T., Bubier, J. L., Frolking, S. & Moore, T. R. Interannual variability in the peatland-atmosphere carbon dioxide exchange at an ombrotrophic bog. *Global Biogeochemical Cycles* **17**, 2002GB001983 (2003).

239. Lagergren, F. *et al.* Net primary production and light use efficiency in a mixed coniferous forest in Sweden. *Plant Cell & Environment* **28**, 412–423 (2005).

240. Lagergren, F. *et al.* Thinning effects on pine-spruce forest transpiration in central Sweden. *Forest Ecology and Management* **255**, 2312–2323 (2008).

241. Lai, C., Katul, G., Butnor, J., Ellsworth, D. & Oren, R. Modelling night-time ecosystem respiration by a constrained source optimization method. *Global Change Biology* **8**, 124–141 (2002).

242. Larsen, K. S., Ibrom, A., Jonasson, S., Michelsen, A. & Beier, C. Significance of cold-season respiration and photosynthesis in a subarctic heath ecosystem in Northern Sweden. *Global Change Biology* **13**, 1498–1508 (2007).

243. Lauenroth, W. K. & Sala, O. E. Long-Term Forage Production of North American Shortgrass Steppe. *Ecological Applications* **2**, 397–403 (1992).

244. Lauenroth, W. K. *et al.* Uncertainty in Calculations of Net Primary Production for Grasslands. *Ecosystems* **9**, 843–851 (2006).

245. Law, B. E., Sun, O. J., Campbell, J., Van Tuyl, S. & Thornton, P. E. Changes in carbon storage and fluxes in a chronosequence of ponderosa pine. *Global Change Biology* **9**, 510–524 (2003).

246. Law, B. E. *et al.* Disturbance and climate effects on carbon stocks and fluxes across Western Oregon USA. *Global Change Biology* **10**, 1429–1444 (2004).

247. Law, B. E. *et al.* Environmental controls over carbon dioxide and water vapor exchange of terrestrial vegetation. *Agricultural and Forest Meteorology* **113**, 97–120 (2002).

248. Lawrence, B. A., Fahey, T. J. & Zedler, J. B. Root dynamics of Carex stricta-dominated tussock meadows. *Plant Soil* **364**, 325–339 (2013).

249. Leadley, P. W., Niklaus, P. A., Stocker, R. & Korner, C. A field study of the effects of elevated CO2 on plant biomass and community structure in a calcareous grassland. *Oecologia* **118**, 39–49 (1999).

250. Lee, H., Schuur, E. A. G. & Vogel, J. G. Soil CO_2 production in upland tundra where permafrost is thawing. *J. Geophys. Res.* **115**, 2008JG000906 (2010).

251. Leuschner, C. *et al.* Conversion of tropical moist forest into cacao agroforest: consequences for carbon pools and annual C sequestration. *Agroforest Syst* **87**, 1173–1187 (2013).

252. Li, C.-P. & Xiao, C.-W. Above- and belowground biomass of Artemisia ordosica communities in three contrasting habitats of the Mu Us desert, northern China. *Journal of Arid Environments* **70**, 195–207 (2007).

253. Li, C., Sun, O. J., Xiao, C. & Han, X. Differences in Net Primary Productivity Among Contrasting Habitats in Artemisia ordosica Rangeland of Northern China. *Rangeland Ecology & Management* **62**, 345–350 (2009).

254. Li, J., Lin, S., Taube, F., Pan, Q. & Dittert, K. Above and belowground net primary productivity of grassland influenced by supplemental water and nitrogen in Inner Mongolia. *Plant Soil* **340**, 253–264 (2011).

255. Li, T., Grant, R. F. & Flanagan, L. B. Climate impact on net ecosystem productivity of a semi-arid natural grassland: modelling and measurement. *Agricultural and Forest Meteorology* **126**, 99–116 (2004).

256. Li, X., Fu, H., Guo, D., Li, X. & Wan, C. Partitioning soil respiration and assessing the carbon balance in a Setaria italica (L.) Beauv. Cropland on the Loess Plateau, Northern China. *Soil Biology and Biochemistry* **42**, 337–346 (2010).

257. Li, Y., Zhou, L., Xu, Z. & Zhou, G. Comparison of water vapour, heat and energy exchanges over agricultural and wetland ecosystems. *Hydrological Processes* **23**, 2069–2080 (2009).

258. Li, Z.-G. *et al.* Carbon Dioxide Fluxes and Concentrations in a Cotton Field in Northwestern China:

Effects of Plastic Mulching and Drip Irrigation. *Pedosphere* **21**, 178–185 (2011).

259. Li, Z., Zhang, R., Wang, X., Chen, F. & Tian, C. Growing Season Carbon Dioxide Exchange in Flooded Non-Mulching and Non-Flooded Mulching Cotton. *PLoS ONE* **7**, e50760 (2012).

260. Liberloo, M. *et al.* Coppicing shifts CO_2 stimulation of poplar productivity to above-ground pools: a synthesis of leaf to stand level results from the POP/EUROFACE experiment. *New Phytologist* **182**, 331–346 (2009).

261. Lieth, H. & Werger, M. J. A. *Tropical Rain Forest Ecosystems: Biogeographical and Ecological Studies*. (Elsevier Science, Amsterdam, 2014).

262. Linder, S. NPP Boreal Forest. Oak Ridge National Laboratory Distributed Active Archive Center, Oak Ridge, Tennessee, U.S.A. (1998).

263. Linder, S. & Agren, G. I. NPP Boreal Forest. Oak Ridge National Laboratory Distributed Active Archive Center, Oak Ridge, Tennessee, U.S.A. (1998).

264. Lindroth, A., Klemedtsson, L., Grelle, A., Weslien, P. & Langvall, O. Measurement of net ecosystem exchange, productivity and respiration in three spruce forests in Sweden shows unexpectedly large soil carbon losses. *Biogeochemistry* **89**, 43–60 (2008).

265. Liu, S., Zhang, L., Liu, Q. & Zou, J. Fe(III) fertilization mitigating net global warming potential and greenhouse gas intensity in paddy rice-wheat rotation systems in China. *Environmental Pollution* **164**, 73–80 (2012).

266. Lock, J. M. The Effects of Hippopotamus Grazing on Grasslands. *The Journal of Ecology* **60**, 445 (1972).

267. Long, S. P. *et al.* Primary productivity of natural grass ecosystems of the tropics: A reappraisal. *Plant and Soil* **115**, 155–166 (1989).

268. Lukac, M., Calfapietra, C. & Godbold, D. L. Production, turnover and mycorrhizal colonization of root systems of three *Populus* species grown under elevated CO_2 (POPFACE). *Global Change*

Biology **9**, 838–848 (2003).

269. Lundin, L.-C. *et al.* Continuous long-term measurements of soil-plant-atmosphere variables at a forest site. *Agricultural and Forest Meteorology* **98–99**, 53–73 (1999).

270. Luo, Y., Jackson, R. B., Field, C. B. & Mooney, H. A. Elevated CO2 increases belowground respiration in California grasslands. *Oecologia* **108**, 130–137 (1996).

271. Maass, M. & Martinez-Yrizar, A. NPP Tropical Forest. Oak Ridge National Laboratory Distributed Active Archive Center, Oak Ridge, Tennessee, U.S.A. (2001).

272. Mack, M. C., Schuur, E. A. G., Bret-Harte, M. S., Shaver, G. R. & Chapin, F. S. Ecosystem carbon storage in arctic tundra reduced by long-term nutrient fertilization. *Nature* **431**, 440–443 (2004).

273. Maher, R. M., Asbjornsen, H., Kolka, R. K., Cambardella, C. A. & Raich, J. W. Changes in soil respiration across a chronosequence of tallgrass prairie reconstructions. *Agriculture, Ecosystems & Environment* **139**, 749–753 (2010).

274. Maier, C. A., Albaugh, T. J., Lee Allen, H. & Dougherty, P. M. Respiratory carbon use and carbon storage in mid-rotation loblolly pine (*Pinus taeda* L.) plantations: the effect of site resources on the stand carbon balance. *Global Change Biology* **10**, 1335–1350 (2004).

275. Majdi, H. Changes in fine root production and longevity in relation to water and nutrient availability in a Norway spruce stand in northern Sweden. *Tree Physiology* **21**, 1057–1061 (2001).

276. Major, J., Lehmann, J., Rondon, M. & Goodale, C. Fate of soil-applied black carbon: downward migration, leaching and soil respiration. *Global Change Biology* **16**, 1366–1379 (2010).

277. Malhi, Y., Baldocchi, D. D. & Jarvis, P. G. The carbon balance of tropical, temperate and boreal forests. *Plant Cell & Environment* **22**, 715–740 (1999).

278. Malhi, Y. *et al.* Comprehensive assessment of carbon productivity, allocation and storage in three Amazonian forests. *Global Change Biology* **15**, 1255–1274 (2009).

279. Malhi, Y. *et al.* The above-ground coarse wood productivity of 104 Neotropical forest plots. *Global*

Change Biology **10**, 563–591 (2004).

280. Malhi, Y. *et al.* Carbon dioxide transfer over a Central Amazonian rain forest. *J. Geophys. Res.* **103**, 31593–31612 (1998).

281. Malmer, N. & Wallen, B. Peat Formation and Mass Balance in Subarctic Ombrotrophic Peatland around Abisko, Northern Scandinavia. *Ecological Bulletins* **45**, 79–92 (1996).

282. Manies, K. L., Harden, J. W., Veldhuis, H. & Trumbore, S. E. *Soil Data from a Moderately Well and Somewhat Poorly Drained Fire Chroosequence near Thompson, Manitoba, Canada*. http://pubs.usgs.gov/of/2006/1291 (2006).

283. Mareschal, L. *et al.* Mineralogical and physico-chemical properties of Ferralic Arenosols derived from unconsolidated Plio-Pleistocenic deposits in the coastal plains of Congo. *Geoderma* **162**, 159–170 (2011).

284. Marsden, C. *et al.* Modifying the G'DAY process-based model to simulate the spatial variability of Eucalyptus plantation growth on deep tropical soils. *Forest Ecology and Management* **301**, 112–128 (2013).

285. Martinez-Mena, M., Lopez, J., Almagro, M., Boix-Fayos, C. & Albaladejo, J. Effect of water erosion and cultivation on the soil carbon stock in a semiarid area of South-East Spain. *Soil and Tillage Research* **99**, 119–129 (2008).

286. Martinez-Yrizar, A., Maass, J. M., Perez-Jimenez, L. A. & Sarukhan, J. Net Primary Productivity of a Tropical Deciduous Forest Ecosystem in Western Mexico. *Journal of Tropical Ecology* **12**, 169–175 (1996).

287. Matson, P., Johnson, L., Billow, C., Miller, J. & Pu, R. Seasonal Patterns and Remote Spectral Estimation of Canopy Chemistry Across the Oregon Transect. *Ecological Applications* **4**, 280–298 (1994).

288. McClaugherty, C. A., Aber, J. D. & Melillo, J. M. The Role of Fine Roots in the Organic Matter and

Nitrogen Budgets of Two Forested Ecosystems. *Ecology* **63**, 1481–1490 (1982).

289. Mcculley, R. L. & Jackson, R. B. Conversion of Tallgrass Prairie to Woodland: Consequences for Carbon and Nitrogen Cycling. *The American Midland Naturalist* **167**, 307–321 (2012).

290. McCulley, R. L. *et al.* Regional Patterns in Carbon Cycling Across the Great Plains of North America. *Ecosystems* **8**, 106–121 (2005).

291. McEwan, R. W., Muller, R. N., Arthur, M. A. & Housman, H. H. Temporal and Ecological Patterns of Flowering Dogwood Mortality in the Mixed Mesophytic Forest of Eastern Kentucky. *Journal of the Torrey Botanical Society* **127**, 221 (2000).

292. McNaughton, S. J. Structure and Function in California Grasslands. *Ecology* **49**, 962–972 (1968).

293. Menaut, J. C. & Cesar, J. Structure and Primary Productivty of Lamto Savannas, Ivory Coast. *Ecology* **60**, 1197–1210 (1979).

294. Merbold, L. *et al.* Precipitation as driver of carbon fluxes in 11 African ecosystems. *Biogeosciences* **6**, 1027–1041 (2009).

295. Metcalfe, D. B. *et al.* Shifts in plant respiration and carbon use efficiency at a large-scale drought experiment in the eastern Amazon. *New Phytologist* **187**, 608–621 (2010).

296. Michelsen, A., Schmidt, I. K., Jonasson, S., Quarmby, C. & Sleep, D. Leaf 15N abundance of subarctic plants provides field evidence that ericoid, ectomycorrhizal and non-and arbuscular mycorrhizal species access different sources of soil nitrogen. *Oecologia* **105**, 53–63 (1996).

297. Mielnick, P., Dugas, W. A., Mitchell, K. & Havstad, K. Long-term measurements of CO2 flux and evapotranspiration in a Chihuahuan desert grassland. *Journal of Arid Environments* **60**, 423–436 (2005).

298. Milchunas, D. G. & Lauenroth, W. K. Carbon Dynamics and Estimates of Primary Production by Harvest, ^(14)C Dilution, and ^(14)C Turnover. *Ecology* **73**, 593–607 (1992).

299. Milchunas, D. G. & Lauenroth, W. K. Belowground Primary Production by Carbon Isotope Decay and

Long-term Root Biomass Dynamics. *Ecosystems* **4**, 139–150 (2001).

300. Milchunas, D. G., Morgan, J. A., Mosier, A. R. & LeCain, D. R. Root dynamics and demography in shortgrass steppe under elevated CO_2, and comments on minirhizotron methodology. *Global Change Biology* **11**, 1837–1855 (2005).

301. Milchunas, D. G. Estimating Root Production: Comparison of 11 Methods in Shortgrass Steppe and Review of Biases. *Ecosystems* **12**, 1381–1402 (2009).

302. Misson, L., Tang, J., Xu, M., McKay, M. & Goldstein, A. Influences of recovery from clear-cut, climate variability, and thinning on the carbon balance of a young ponderosa pine plantation. *Agricultural and Forest Meteorology* **130**, 207–222 (2005).

303. Mitchell, J. E., West, N. E. & Miller, R. W. Soil Physical Properties in Relation to Plant Community Patterns in the Shadscale Zone of Northwestern Utah. *Ecology* **47**, 627–630 (1966).

304. Mollicone, D. *et al.* A Model-Based Approach for Estimation of Carbon Sinks in European Forests. in *Fluxes of carbon water and energy of European forests. Ecological Applications* vol. 163 179–206 (Springer-Verlag, 2003).

305. Moore, S. *et al.* Forest biomass, productivity and carbon cycling along a rainfall gradient in West Africa. *Global Change Biology* **24**, (2018).

306. Moore, T. R., Bubier, J. L., Frolking, S. E., Lafleur, P. M. & Roulet, N. T. Plant biomass and production and CO2 exchange in an ombrotrophic bog. *Journal of Ecology* **90**, 25–36 (2002).

307. Morel, A. C. *et al.* Carbon dynamics, net primary productivity and human-appropriated net primary productivity across a forest–cocoa farm landscape in West Africa. *Global Change Biology* **25**, 2661–2677 (2019).

308. Moscatelli, M. C., Lagomarsino, A., De Angelis, P. & Grego, S. Short- and medium-term contrasting effects of nitrogen fertilization on C and N cycling in a poplar plantation soil. *Forest Ecology and Management* **255**, 447–454 (2008).

309. Moser, G. *et al.* Elevation effects on the carbon budget of tropical mountain forests (S Ecuador): the role of the belowground compartment: elevation effects on forest carbon cycling. *Global Change Biology* **17**, 2211–2226 (2011).

310. Mosier, A. R., Pendall, E. & Morgan, J. A. Effect of water addition and nitrogen fertilization on the fluxes of CH_4, CO_2, NO_x, and N_2O following five years of elevated CO_2; in the Colorado Shortgrass Steppe. *Atmos. Chem. Phys.* **3**, 1703–1708 (2003).

311. Moureaux, C., Debacq, A., Bodson, B., Heinesch, B. & Aubinet, M. Annual net ecosystem carbon exchange by a sugar beet crop. *Agricultural and Forest Meteorology* **139**, 25–39 (2006).

312. Mu, Z., Kimura, S. D., Toma, Y. & Hatano, R. Evaluation of the soil carbon budget under different upland cropping systems in central Hokkaido, Japan. *Soil Science and Plant Nutrition* **54**, 650–661 (2008).

313. Murphy, M. T. & Moore, T. R. Linking root production to aboveground plant characteristics and water table in a temperate bog. *Plant Soil* **336**, 219–231 (2010).

314. Murthy, R., Dougherty, P. M., Zarnoch, S. J. & Allen, H. L. Effects of carbon dioxide, fertilization, and irrigation on photosynthetic capacity of loblolly pine trees. *Tree Physiology* **16**, 537–546 (1996).

315. Nadelhoffer, K. J., Johnson, L., Laundre, J., Giblin, A. E. & Shaver, G. R. Fine root production and nutrient content in wet and moist arctic tundras as influenced by chronic fertilization. *Plant and Soil* **242**, 107–113 (2002).

316. Naeth, M. A., Chanasyk, D. S. & Bailey, A. W. Applicability of the Kostiakov Equation to Mixed Prairie and Fescue Grasslands of Alberta. *Journal of Range Management* **44**, 18 (1991).

317. Natali, S. M., Schuur, E. A. G. & Rubin, R. L. Increased plant productivity in Alaskan tundra as a result of experimental warming of soil and permafrost. *Journal of Ecology* **100**, 488–498 (2012).

318. Natali, S. M. *et al.* Effects of experimental warming of air, soil and permafrost on carbon balance in Alaskan tundra: warming of Alaskan tundra. *Global Change Biology* **17**, 1394–1407 (2011).

319. Navarro, M. N. V. *et al.* Fruit development, not GPP, drives seasonal variation in NPP in a tropical palm plantation. *Tree Physiology* **28**, 1661–1674 (2008).

320. Nave, L. E., Vogel, C. S., Gough, C. M. & Curtis, P. S. Contribution of atmospheric nitrogen deposition to net primary productivity in a northern hardwood forest. *Can. J. For. Res.* **39**, 1108–1118 (2009).

321. Niklaus, P. A., *et al*. Nutrient relations in calcareous grassland under elevated CO 2. *Oecologia* **116**, 67–75 (1998).

322. Nilsson, L.-O. & Wiklund, K. Influence of nutrient and water stress on Norway spruce production in south Sweden - the role of air pollutants. *Plant Soil* **147**, 251–265 (1992).

323. Niu, S., Sherry, R. A., Zhou, X., Wan, S. & Luo, Y. Nitrogen regulation of the climate—carbon feedback: evidence from a long-term global change experiment. *Ecology* **91**, 3261–3273 (2010).

324. Notaro, M. *et al.* Complex seasonal cycle of ecohydrology in the Southwest United States. *J. Geophys. Res.* **115**, 2010JG001382 (2010).

325. Oechel, W. C. & Sveinbjornssön, B. Primary production processes in arctic bryophytes at Barrow, Alaska. in *Vegetation and production ecology of an Alaskan arctic tundra* 679 (Springer-Verlag, 1978).

326. Oechel, W. C., Vourlitis, G. L., Brooks, S., Crawford, T. L. & Dumas, E. Intercomparison among chamber, tower, and aircraft net CO_2 and energy fluxes measured during the Arctic System Science Land-Atmosphere-Ice Interactions (ARCSS-LAII) Flux Study. *J. Geophys. Res.* **103**, 28993–29003 (1998).

327. Ohtsuka, T. *et al.* Biometric based estimates of net primary production (NPP) in a cool-temperate deciduous forest stand beneath a flux tower. *Agricultural and Forest Meteorology* **134**, 27–38 (2005).

328. Ohtsuka, T., Mo, W., Satomura, T., Inatomi, M. & Koizumi, H. Biometric Based Carbon Flux

Measurements and Net Ecosystem Production (NEP) in a Temperate Deciduous Broad-Leaved Forest Beneath a Flux Tower. *Ecosystems* **10**, 324–334 (2007).

329. Ohtsuka, T., Negishi, M., Sugita, K., Iimura, Y. & Hirota, M. Carbon cycling and sequestration in a Japanese red pine (*Pinus densiflora*) forest on lava flow of Mt. Fuji. *Ecological Research* **28**, 855–867 (2013).

330. Ohtsuka, T., Saigusa, N. & Koizumi, H. On linking multiyear biometric measurements of tree growth with eddy covariance-based net ecosystem production. *Global Change Biology* **15**, 1015–1024 (2009).

331. Olefeldt, D. *et al.* Net carbon accumulation of a high-latitude permafrost palsa mire similar to permafrost-free peatlands. *Geophysical Research Letters* **39**, 2011GL050355 (2012).

332. Olefeldt, D. & Roulet, N. T. Effects of permafrost and hydrology on the composition and transport of dissolved organic carbon in a subarctic peatland complex. *J. Geophys. Res.* **117**, 2011JG001819 (2012).

333. Olofsson, J. Effects of Simulated Reindeer Grazing, Trampling, and Waste Products on Nitrogen Mineralization and Primary Production. *Arctic, Antarctic, and Alpine Research* **41**, 330–338 (2009).

334. Østebye. in *Structure and function of tundra ecosystems* (eds. Rosswall, T. & Heal, O. W.) (Stockholm, 1975).

335. Ostertag, R. Effects of nitrogen and phosphorus availability on fine-root dynamics in Hawaiian montane forests. **82**, (2001).

336. Owensby, C. E., Coyne, P. I., Ham, J. M., Auen, L. M. & Knapp, A. K. Biomass Production in a Tallgrass Prairie Ecosystem Exposed to Ambient and Elevated CO_2. *Ecological Applications* **3**, 644–653 (1993).

337. Owensby, C. E., Ham, Jay. M., Knapp, Alan. K. & Auen, Lisa. M. Biomass production and species composition change in a tallgrass prairie ecosystem after long-term exposure to elevated

atmospheric CO_2. *Global Change Biology* **5**, 497–506 (1999).

338. Pandey, C. B. & Singh, J. S. Rainfall and Grazing Effects on Net Primary Productivity in a Tropical Savanna, India. *Ecology* **73**, 2007–2021 (1992).

339. Paustian, K. *et al.* Carbon and Nitrogen budgets of four agro-ecosystems with annual and perennial crops, with and without N fertilization. *The Journal of Applied Ecology* **27**, 60 (1990).

340. Paw U, K. *et al.* Carbon dioxide exchange between an old-growth forest and the atmosphere. *Ecosystems* **7**, (2004).

341. Pearson, L. C. Primary production in grazed and ungrazed desert communities of eastern Idaho. *Ecology* **46**, 278–285 (1965).

342. Peichl, M., Arain, M. A., Ullah, S. & Moore, T. R. Carbon dioxide, methane, and nitrous oxide exchanges in an age-sequence of temperate pine forests. *Global Change Biology* **16**, 2198–2212 (2010).

343. Peichl, M., Brodeur, J. J., Khomik, M. & Arain, M. A. Biometric and eddy-covariance based estimates of carbon fluxes in an age-sequence of temperate pine forests. *Agricultural and Forest Meteorology* **150**, 952–965 (2010).

344. Pendall, E. *et al.* Elevated atmospheric CO_2 effects and soil water feedbacks on soil respiration components in a Colorado grassland. *Global Biogeochemical Cycles* **17**, 2001GB001821 (2003).

345. Peregon, A., Maksyutov, S., Kosykh, N. P. & Mironycheva-Tokareva, N. P. Map-based inventory of wetland biomass and net primary production in western Siberia. *J. Geophys. Res.* **113**, 2007JG000441 (2008).

346. Pérez, C. A. & Frangi, J. L. Grassland biomass dynamics along an altitudinal gradient in the Pampa. *Journal of Range Management* **53**, 410-414 (2000).

347. Petrie, M. D., Collins, S. L., Swann, A. M., Ford, P. L. & Litvak, M. E. Grassland to shrubland state transitions enhance carbon sequestration in the northern Chihuahuan Desert. *Global Change*

Biology **21**, 1226–1235 (2015).

348. Petrie, M. D., Collins, S. L., Swann, A. M., Ford, P. L. & Litvak, M. E. Grassland to shrubland state transitions enhance carbon sequestration in the northern Chihuahuan Desert. *Global Change Biology* **21**, 1226–1235 (2015).

349. Pilegaard, K., Hummelshøj, P., Jensen, N. O. & Chen, Z. Two years of continuous CO2 eddy-flux measurements over a Danish beech forest. *Agricultural and Forest Meteorology* **107**, 29–41 (2001).

350. Pinno, B. D. & Wilson, S. D. Ecosystem carbon changes with woody encroachment of grassland in the northern Great Plains. *Écoscience* **18**, 157–163 (2011).

351. Pinno, B. D. & Wilson, S. D. Fine root response to soil resource heterogeneity differs between grassland and forest. *Plant Ecol* **214**, 821–829 (2013).

352. Post, A. K. & Knapp, A. K. The importance of extreme rainfall events and their timing in a semi-arid grassland. *Journal of Ecology* **108**, 2431–2443 (2020).

353. Pucheta, E., Bonamici, I., Cabido, M. & Díaz, S. Below-ground biomass and productivity of a grazed site and a neighbouring ungrazed exclosure in a grassland in central Argentina. *Austral Ecology* **29**, 201–208 (2004).

354. Pucheta, E., Cabido, M., Díaz, S. & Funes, G. Floristic composition, biomass, and aboveground net plant production in grazed and protected sites in a mountain grassland of central Argentina. *Acta Oecologica* **19**, 97–105 (1998).

355. Quesada, C. A. *et al.* Variations in chemical and physical properties of Amazon forest soils in relation to their genesis. *Biogeosciences* **7**, 1515–1541 (2010).

356. Raich, J. W. & Nadelhoffer, K. J. Belowground Carbon Allocation in Forest Ecosystems: Global Trends. *Ecology* **70**, 1346–1354 (1989).

357. Ram, J., Singh, J. S. & Singh, S. P. Plant biomass, species diversity and net primary production in a central Himalayan high altitude grassland. *The Journal of Ecology* **77**, 456 (1989).

358. Ram, J., Singh, S. P. & Singh, J. S. Effect of fertilizer on plant biomass distribution and net accumulation rate in an alpine meadow in central Himalaya, India. *Journal of range management* **44**, 140-143 (1991).

359. Redente, E. F., Biondini, M. E. & Moore, J. C. Observations on Biomass Dynamics of a Crested Wheatgrass and Native Shortgrass Ecosystem in Southern Wyoming. *Journal of Range Management* **42**, 113 (1989).

360. *The True Prairie Ecosystem*. (Hutchinson Ross Pub. Co. ; distributed world wide by Academic Press, Stroudsburg, Pa. : [New York], 1981).

361. Rocha, A. V. & Goulden, M. L. Large interannual CO_2 and energy exchange variability in a freshwater marsh under consistent environmental conditions. *J. Geophys. Res.* **113**, 2008JG000712 (2008).

362. Rocha, W. *et al.* Ecosystem productivity and carbon cycling in intact and annually burnt forest at the dry southern limit of the Amazon rainforest (Mato Grosso, Brazil). *Plant Ecology & Diversity* **7**, 25–40 (2014).

363. Rose, K. K., Hild, A. L., Whitson, T. D., Koch, D. W. & Tassell, L. V. Competitive Effects of Cool-Season Grasses on Re-Establishment of Three Weed Species. *Weed Technology* **15**, 885–891 (2001).

364. Roupsard, O. *et al.* Partitioning energy and evapo-transpiration above and below a tropical palm canopy. *Agricultural and Forest Meteorology* **139**, 252–268 (2006).

365. Ruess, R. W. *et al.* Coupling fine root dynamics with ecosystem carbon cycling in black spruce forests of interior Alaska. *Ecological Monographs* **73**, 643–662 (2003).

366. Runyon, J., Waring, R. H., Goward, S. N. & Welles, J. M. Environmental Limits on Net Primary Production and Light-Use Efficiency Across the Oregon Transect. *Ecological Applications* **4**, 226–237 (1994).

367. Russell, A. E., Laird, D. A., Parkin, T. B. & Mallarino, A. P. Impact of Nitrogen Fertilization and Cropping System on Carbon Sequestration in Midwestern Mollisols. *Soil Science Soc of Amer J* **69**,

413–422 (2005).

368. Russell, A. E., Raich, J. W., Valverde-Barrantes, O. J. & Fisher, R. F. Tree Species Effects on Soil Properties in Experimental Plantations in Tropical Moist Forest. *Soil Science Soc of Amer J* **71**, 1389–1397 (2007).

369. Russell, A. E., Cambardella, C. A., Laird, D. A., Jaynes, D. B. & Meek, D. W. Nitrogen fertilizer effects on soil carbon balances in Midwestern U.S. agricultural systems. *Ecological Applications* **19**, 1102–1113 (2009).

370. Russell, A. E., Raich, J. W., Arrieta, R. B., Valverde-Barrantes, O. & González, E. Impacts of individual tree species on carbon dynamics in a moist tropical forest environment. *Ecological Applications* **20**, 1087–1100 (2010).

371. Ryan, M. G., Hubbard, R. M., Pongracic, S., Raison, R. J. & McMurtrie, R. E. Foliage, fine-root, woody-tissue and stand respiration in Pinus radiata in relation to nitrogen status. *Tree Physiology* **16**, 333–343 (1996).

372. Ryan, M. G., Lavigne, M. B. & Gower, S. T. Annual carbon cost of autotrophic respiration in boreal forest ecosystems in relation to species and climate. *J. Geophys. Res.* **102**, 28871–28883 (1997).

373. Saigusa, N., Yamamoto, S., Murayama, S., Kondo, H. & Nishimura, N. Gross primary production and net ecosystem exchange of a cool-temperate deciduous forest estimated by the eddy covariance method. *Agricultural and Forest Meteorology* **112**, 203–215 (2002).

374. Saigusa, N., Yamamoto, S., Murayama, S. & Kondo, H. Inter-annual variability of carbon budget components in an AsiaFlux forest site estimated by long-term flux measurements. *Agricultural and Forest Meteorology* **134**, 4–16 (2005).

375. Saito, M., Miyata, A., Nagai, H. & Yamada, T. Seasonal variation of carbon dioxide exchange in rice paddy field in Japan. *Agricultural and Forest Meteorology* **135**, 93–109 (2005).

376. Saitoh, T. M. *et al.* Carbon dioxide exchange in a cool-temperate evergreen coniferous forest over

complex topography in Japan during two years with contrasting climates. *J Plant Res* **123**, 473–483 (2010).

377. Saiz, G. *et al.* Seasonal and spatial variability of soil respiration in four Sitka spruce stands. *Plant Soil* **287**, 161–176 (2006).
378. San Jose, J., Montes, R., Grace, J. & Nikonova, N. Land-use changes alter CO2 flux patterns of a tall-grass Andropogon field and a savanna-woodland continuum in the Orinoco lowlands. *Tree Physiology* **28**, 437–450 (2008).
379. Sanderman, J. & Amundson, R. A comparative study of dissolved organic carbon transport and stabilization in California forest and grassland soils. *Biogeochemistry* **89**, 309–327 (2008).
380. Sanderman, J., Baldock, J. A. & Amundson, R. Dissolved organic carbon chemistry and dynamics in contrasting forest and grassland soils. *Biogeochemistry* **89**, 181–198 (2008).
381. Satomura, T., Hashimoto, Y., Koizumi, H., Nakane, K. & Horikoshi, T. Seasonal patterns of fine root demography in a cool-temperate deciduous forest in central Japan. *Ecological Research* **21**, 741–753 (2006).
382. Saxena, A. K., Rana, B. S., Rao, O. P. & Singh, B. P. Seasonal variation in biomass and primary productivity of para grass (Brachiaria mutica) under a mixed tree stand and in an adjacent open area in northern India. *Agroforest Syst* **33**, 75–85 (1996).
383. Schippi, B. & Korner, C. Growth responses of an alpine grassland to elevated CO2. *Oecologia* **105**, 43–52 (1996).
384. Schmidt, I. K., Jonasson, S., Shaver, G. R., Michelsen, A. & Nordin, A. Mineralization and distribution of nutrients in plants and microbes in four arctic ecosystems: responses to warming. *Plant and Soil* **242**, 93–106 (2002).
385. Schöning, I. & Kögel-Knabner, I. Chemical composition of young and old carbon pools throughout Cambisol and Luvisol profiles under forests. *Soil Biology and Biochemistry* **38**, 2411–2424 (2006).

386. *Carbon and Nitrogen Cycling in European Forest Ecosystems*. (Springer, Berlin ; New York, 2000).

387. Schulze, E.-D. *et al.* Interactions Between the Carbon and Nitrogen Cycles and the Role of Biodiversity: A Synopsis of a Study Along a North-South Transect Through Europe. in *Carbon and Nitrogen Cycling in European Forest Ecosystems* (ed. Schulze, E.-D.) vol. 142 468–491 (Springer Berlin Heidelberg, Berlin, Heidelberg, 2000).

388. Schuur, E. A. G., Crummer, K. G., Vogel, J. G. & Mack, M. C. Plant species composition and productivity following permafrost thaw and thermokarst in Alaskan tundra. *Ecosystems* **10**, 280–292 (2007).

389. Schwärzel, K. *et al.* Soil water content measurements deliver reliable estimates of water fluxes: A comparative study in a beech and a spruce stand in the Tharandt forest (Saxony, Germany). *Agricultural and Forest Meteorology* **149**, 1994–2006 (2009).

390. Scott, J., Stewart, D. & Metherell, A. Alteration of pasture root carbon turnover in response to superphosphate and irrigation at Winchmore New Zealand. *New Zealand Journal of Agricultural Research* **55**, 147–159 (2012).

391. Scurlock, J. M. O., Johnson, K. & Olson, R. J. Estimating net primary productivity from grassland biomass dynamics measurements. *Global Change Biology* **8**, 736–753 (2002).

392. Sellers, P. J. *et al.* BOREAS in 1997: Experiment overview, scientific results, and future directions. *J. Geophys. Res.* **102**, 28731–28769 (1997).

393. Senthilkumar, K., Manian, S., Udaiyan, K. & Paulsamy, S. Elevated biomass production in burned natural grasslands in southern India. *Tropical grasslands* **32**, 50–63 (1998).

394. Shankar, U., Pandey, H. N. & Tripathi, R. S. Phytomass dynamics and primary productivity in humid grasslands along altitudinal and rainfall gradients. *Acta Oecologica* **14**, 197–209 (1993).

395. Shaver, G. R. & Billings, W. D. Root Production and Root Turnover in a Wet Tundra Ecosystem, Barrow, Alaska. *Ecology* **56**, 401–409 (1975).

396. Shaver, G. R. *et al.* Biomass and CO_2 Flux in Wet Sedge Tundras: Responses to Nutrients, Temperature, and Light. *Ecological Monographs* **68**, 75 (1998).

397. Shaver, G. R. *et al.* Species composition interacts with fertilizer to control lon-term change in tundra productivity. *Ecology* **82**, 3163–3181 (2001).

398. Shaw, DavidC. *et al.* Ecological Setting of the Wind River Old-growth Forest. *Ecosystems* **7**, (2004).

399. Sherry, R. A. *et al.* Lagged effects of experimental warming and doubled precipitation on annual and seasonal aboveground biomass production in a tallgrass prairie. *Global Change Biology* **14**, 2923–2936 (2008).

400. Sims, P. L. & Bradford, J. A. Carbon dioxide fluxes in a southern plains prairie. *Agricultural and Forest Meteorology* **109**, 117-134 (2001).

401. Sims, P. L. & Singh, J. S. The Structure and Function of Ten Western North American Grasslands: III. Net Primary Production, Turnover and Efficiencies of Energy Capture and Water Use. *The Journal of Ecology* **66**, 573 (1978).

402. Sims, P. L., Singh, J. S. & Lauenroth, W. K. The Structure and Function of Ten Western North American Grasslands: I. Abiotic and Vegetational Characteristics. *The Journal of Ecology* **66**, 251 (1978).

403. Singh, J. S., Raghubanshi, A. S., Singh, R. S. & Srivastava, S. C. Microbial biomass acts as a source of plant nutrients in dry tropical forest and savanna. *Nature* **338**, 499–500 (1989).

404. Singh, J. S., Raghubanshi, A. S., Singh, R. S. & Srivastava, S. C. Microbial biomass acts as a source of plant nutrients in dry tropical forest and savanna. *Nature* **338**, 499–500 (1989).

405. Singh, J. S. & Yadava, P. S. Seasonal Variation in Composition, Plant Biomass, and Net Primary Productivity of a Tropical Grassland at Kurukshetra, India. *Ecological Monographs* **44**, 351–376 (1974).

406. Singh, R. S., Raghubanshi, A. S. & Singh, J. S. Nitrogen-mineralization in dry tropical savanna: Effects

of burning and grazing. *Soil Biology and Biochemistry* **23**, 269–273 (1991).

407. Snyman, H. A. Short-term response of rangeland botanical composition and productivity to fertilization (N and P) in a semi-arid climate of South Africa. *Journal of Arid Environments* **50**, 167–183 (2002).

408. Snyman, H. A. Rangeland degradation in a semi-arid South Africa—I: influence on seasonal root distribution, root/shoot ratios and water-use efficiency. *Journal of Arid Environments* **60**, 457–481 (2005).

409. Snyman, H. A. & Du Preez, C. C. Rangeland degradation in a semi-arid South Africa—II: influence on soil quality. *Journal of Arid Environments* **60**, 483–507 (2005).

410. Snyman, H. A. & Du Preez, C. C. Rangeland degradation in a semi-arid South Africa—II: influence on soil quality. *Journal of Arid Environments* **60**, 483–507 (2005).

411. Sonesson, M., Wielgolaski, F. E. & Kallio, P. Description of Fennoscandian Tundra Ecosystems. in *Fennoscandian Tundra Ecosystems* (ed. Wielgolaski, F. E.) vol. 16 3–28 (Springer Berlin Heidelberg, Berlin, Heidelberg, 1975).

412. Song, C., Xu, X., Tian, H. & Wang, Y. Ecosystem–atmosphere exchange of CH4 and N2O and ecosystem respiration in wetlands in the Sanjiang Plain, Northeastern China. *Global Change Biology* **15**, 692–705 (2009).

413. Sørensen, L. I., Kytöviita, M.-M., Olofsson, J. & Mikola, J. Soil feedback on plant growth in a sub-arctic grassland as a result of repeated defoliation. *Soil Biology and Biochemistry* **40**, 2891–2897 (2008).

414. Steele, S. J., Gower, S. T., Vogel, J. G. & Norman, J. M. Root mass, net primary production and turnover in aspen, jack pine and black spruce forests in Saskatchewan and Manitoba, Canada. *Tree Physiology* **17**, 577–587 (1997).

415. Steffens, M., Kölbl, A., Totsche, K. U. & Kögel-Knabner, I. Grazing effects on soil chemical and

physical properties in a semiarid steppe of Inner Mongolia (P.R. China). *Geoderma* **143**, 63–72 (2008).

416. Steinaker, D. F. & Wilson, S. D. Belowground Litter Contributions to Nitrogen Cycling at a Northern Grassland-Forest Boundary. *Ecology* **86**, 2825–2833 (2005).

417. Steltzer, H. & Bowman, W. D. Differential Influence of Plant Species on Soil Nitrogen Transformations Within Moist Meadow Alpine Tundra. *Ecosystems* **1**, 464–474 (1998).

418. Strugnell, R. G. & Pigott, C. D. Biomass, Shoot-Production and Grazing of Two Grasslands in the Rwenzori National Park, Uganda. *The Journal of Ecology* **66**, 73 (1978).

419. Sullivan, P. F., Arens, S. J. T., Chimner, R. A. & Welker, J. M. Temperature and Microtopography Interact to Control Carbon Cycling in a High Arctic Fen. *Ecosystems* **11**, 61–76 (2008).

420. Sullivan, P. F. *et al.* Climate and species affect fine root production with long-term fertilization in acidic tussock tundra near Toolik Lake, Alaska. *Oecologia* **153**, 643–652 (2007).

421. Sumiyoshi, Y. *et al.* Belowground impacts of perennial grass cultivation for sustainable biofuel feedstock production in the tropics. *GCB Bioenergy* **9**, 694–709 (2017).

422. Sun, O. J., Campbell, J., Law, B. E. & Wolf, V. Dynamics of carbon stocks in soils and detritus across chronosequences of different forest types in the Pacific Northwest, USA. *Global Change Biology* **10**, 1470–1481 (2004).

423. Sundaravalli, M. & Paliwal, K. Primary production and soil carbon dioxide emission in the semi-arid grazing lands of Modurai, India. *Tropical grasslands* **34**, 14–20 (2000).

424. Sundriyal, R. C. Structure, productivity and energy flow in an alpine grassland in the Garhwal Himalaya. *J Vegetation Science* **3**, 15–20 (1992).

425. Suyker, A. E. & Verma, S. B. Gross primary production and ecosystem respiration of irrigated and rainfed maize–soybean cropping systems over 8 years. *Agricultural and Forest Meteorology* **165**, 12–24 (2012).

426. Tan, Z. *et al.* Carbon balance of a primary tropical seasonal rain forest. *J. Geophys. Res.* **115**, 2009JD012913 (2010).

427. Tanabe, H., Abe, Y., Nakano, T. & Tange, T. Carbon and nitrogen changes in A0 horizons in a Pinus densiflora forest established on a Mt. Fuji lava flow. *Jpn.J.For.Environmen* **48**, 1–8 (2006).

428. Tang, J., Qi, Y., Xu, M., Misson, L. & Goldstein, A. H. Forest thinning and soil respiration in a ponderosa pine plantation in the Sierra Nevada. *Tree Physiology* **25**, 57–66 (2005).

429. Tang, J.-W., Cao, M., Zhang, J.-H. & Li, M.-H. Litterfall production, decomposition and nutrient use efficiency varies with tropical forest types in Xishuangbanna, SW China: a 10-year study. *Plant Soil* **335**, 271–288 (2010).

430. Tao, B., Song, C. & Guo, Y. Short-term Effects of Nitrogen Additions and Increased Temperature on Wetland Soil Respiration, Sanjiang Plain, China. *Wetlands* **33**, 727–736 (2013).

431. Tateno, R., Hishi, T. & Takeda, H. Above- and belowground biomass and net primary production in a cool-temperate deciduous forest in relation to topographical changes in soil nitrogen. *Forest Ecology and Management* **193**, 297–306 (2004).

432. Tateno, R. & Takeda, H. Forest structure and tree species distribution in relation to topography-mediated heterogeneity of soil nitrogen and light at the forest floor. *Ecological Research* **18**, 559–571 (2003).

433. Tha Paw U, K. *et al.* Carbon Dioxide Exchange between an Old-Growth Forest and the Atmosphere. *Ecosystems* **7**, 513–524 (2004).

434. Thomas, M. V. *et al.* Carbon dioxide fluxes over an ancient broadleaved deciduous woodland in southern England. *Biogeosciences* **8**, 1595–1613 (2011).

435. Tiedmann, A. R. & Klemmendson, J. O. The influence of western Juniper development on soil nutrient availability. *Nortwest Science* **69**, (1995).

436. Tieszen, L. L. The Seasonal Course of Aboveground Production and Chlorophyll Distribution in a

Wet Arctic Tundra at Barrow, Alaska. *Arctic and Alpine Research* **4**, 307–324 (1972).

437. *Vegetation and Production Ecology of an Alaskan Arctic Tundra*. vol. 29 (Springer New York, New York, NY, 1978).

438. Titlyanova, A. A., Romanova, I. P., Kosykh, N. P. & Mironycheva-Tokareva, N. P. Pattern and process in above-ground and below-ground components of grassland ecosystems. *J Vegetation Science* **10**, 307–320 (1999).

439. Tokida, T. *et al.* Effects of free-air CO2 enrichment (FACE) and soil warming on CH4 emission from a rice paddy field: impact assessment and stoichiometric evaluation. *Biogeosciences* **7**, 2639–2653 (2010).

440. Toma, Y. *et al.* Carbon budget and methane and nitrous oxide emissions over the growing season in a Miscanthus sinensis grassland in Tomakomai, Hokkaido, Japan: C budget in *M. Sinensis* grassland in Japan. *GCB Bioenergy* **3**, 116–134 (2011).

441. Trucco, C. *et al.* Seven-year trends of CO_2 exchange in a tundra ecosystem affected by long-term permafrost thaw. *J. Geophys. Res.* **117**, 2011JG001907 (2012).

442. Trujillo, W., Fisher, M. J. & Lal, R. Root dynamics of native savanna and introduced pastures in the Eastern Plains of Colombia. *Soil and Tillage Research* **87**, 28–38 (2006).

443. Trumbore, S. E., Bubier, J. L., Harden, J. W. & Crill, P. M. Carbon cycling in boreal wetlands: A comparison of three approaches. *J. Geophys. Res.* **104**, 27673–27682 (1999).

444. Turetsky, M. R., Wieder, R. K., Williams, C. J. & Vitt, D. H. Organic matter accumulation, peat chemistry, and permafrost melting in peatlands of boreal Alberta. *Écoscience* **7**, 115–122 (2000).

445. Urrutia-Jalabert, R., Malhi, Y. & Lara, A. The Oldest, Slowest Rainforests in the World? Massive Biomass and Slow Carbon Dynamics of *Fitzroya cupressoides* Temperate Forests in Southern Chile. *PLoS ONE* **10**, e0137569 (2015).

446. Urrutia-Jalabert, R., Malhi, Y. & Lara, A. Soil respiration and mass balance estimation of fine root

production in *Fitzroya cupressoides* forests of southern Chile. *Ecosphere* **8**, e01640 (2017).

447. Valentini, R. *et al.* Seasonal net carbon dioxide exchange of a beech forest with the atmosphere. *Global Change Biology* **2**, 199–207 (1996).

448. Valentini, R. *et al.* Respiration as the main determinant of carbon balance in European forests. *Nature* **404**, 861–865 (2000).

449. *Fluxes of Carbon, Water and Energy of European Forests*. vol. 163 (Springer Berlin Heidelberg, Berlin, Heidelberg, 2003).

450. Valentini, R., Gamon, J. A. & Field, C. B. Ecosystem Gas Exchange in a California Grassland: Seasonal Patterns and Implications for Scaling. *Ecology* **76**, 1940–1952 (1995).

451. van der Molen, M. K. *et al.* The growing season greenhouse gas balance of a continental tundra site in the Indigirka lowlands, NE Siberia. *Biogeosciences* **4**, 985–1003 (2007).

452. van der Valk, A. G. & Davis, C. B. Primary production of prairie glacial marshes. in *Freshwater wetlands: ecological processes and management potential* (Academic Press, New York San Francisco London, 1978).

453. Verlinden, M. S. *et al.* Net ecosystem production and carbon balance of an SRC poplar plantation during its first rotation. *Biomass and Bioenergy* **56**, 412–422 (2013).

454. Verma, S. B. *et al.* Annual carbon dioxide exchange in irrigated and rainfed maize-based agroecosystems. *Agricultural and Forest Meteorology* **131**, 77–96 (2005).

455. Vicca, S. *et al.* Fertile forests produce biomass more efficiently. *Ecology Letters* **15**, 520–526 (2012).

456. Vitousek, P. M., Walker, L. R., Whiteaker, L. D. & Matson, P. A. Nutrient Limitations to Plant Growth during Primary Succession in Hawaii Volcanoes National Park. *Biogeochemistry* **23**, 197–215 (1993).

457. Vogel, J., Schuur, E. A. G., Trucco, C. & Lee, H. Response of CO_2 exchange in a tussock tundra ecosystem to permafrost thaw and thermokarst development. *J. Geophys. Res.* **114**, 2008JG000901 (2009).

458. Vourlitis, G. L., Harazono, Y., Oechel, W. C., Yoshimoto, M. & Mano, M. Spatial and temporal variations in hectare-scale net CO_2 flux, respiration and gross primary production of Arctic tundra ecosystems. *Functional Ecology* **14**, 203–214 (2000).

459. Vourlitis, G. L. *et al.* Nutrient resorption in tropical savanna forests and woodlands of central Brazil. *Plant Ecol* **215**, 963–975 (2014).

460. Vourlitis, G. L., Jaureguy, J., Marin, L. & Rodriguez, C. Shoot and root biomass production in semi-arid shrublands exposed to long-term experimental N input. *Science of The Total Environment* **754**, 142204 (2021).

461. Vourlitis, G. L. & Oechel, W. C. Eddy Covariance Measurements of CO2 and Energy Fluxes of an Alaskan Tussock Tundra Ecosystem. *Ecology* **80**, 686–701 (1999).

462. Vourlitis, G. L. & Pasquini, S. C. Experimental dry-season N deposition alters species composition in southern Californian mediterranean-type shrublands. *Ecology* **90**, 2183–2189 (2009).

463. Vourlitis, G. L., Pasquini, S. C. & Mustard, R. Effects of Dry-Season N Input on the Productivity and N Storage of Mediterranean-Type Shrublands. *Ecosystems* **12**, 473–488 (2009).

464. Vourlitis, G. L. *et al.* Net primary production and ecosystem carbon flux of Brazilian tropical savanna ecosystems from Eddy covariance and inventory methods. *JGR Biogeosciences* **127**, e2021JG006780 (2022).

465. Vourlitis, G. L. *et al.* Variations in Stand Structure and Diversity along a Soil Fertility Gradient in a Brazilian Savanna (Cerrado) in Southern Mato Grosso. *Soil Science Soc of Amer J* **77**, 1370–1379 (2013).

466. Wakhid, N., Hirano, T., Dariah, A. & Agus, F. Net primary production of oil palm plantations on tropical peat. *Mires Peat* **28**, 1–12 (2022).

467. Walker, D. A. *et al.* Vegetation-soil-thaw-depth relationships along a low-arctic bioclimate gradient, Alaska: synthesis of information from the ATLAS studies. *Permafrost & Periglacial* **14**, 103–123

(2003).

468. Walkers, B. & Peters, T. Soils of Truelove Lowland and Plateau. in *Truelove Lowland, Devon Island, Canada: A High Arctic Ecosystem* (ed. Bliss, L. C.) (University of Alberta Press, 1987).

469. Wan, S., Hui, D., Wallace, L. & Luo, Y. Direct and indirect effects of experimental warming on ecosystem carbon processes in a tallgrass prairie. *Global Biogeochemical Cycles* **19**, 2004GB002315 (2005).

470. Wan, S., Xia, J., Liu, W. & work(s):, S. N. R. Photosynthetic Overcompensation under Nocturnal Warming Enhances Grassland Carbon Sequestration. *Ecology* **90**, 2700–2710 (2009).

471. Wang, J. & Epstein, H. E. Estimating carbon source-sink transition during secondary succession in a Virginia valley. *Plant Soil* **362**, 135–147 (2013).

472. Wang, J., Epstein, H. E. & Wang, L. Soil CO_2 flux and its controls during secondary succession. *J. Geophys. Res.* **115**, 2009JG001084 (2010).

473. Wang, J. *et al.* Asymmetry in above- and belowground productivity responses to N addition in a semi-arid temperate steppe. *Global Change Biology* **25**, 2958–2969 (2019).

474. Wang, L., Shaner, P. L. & Macko, S. Foliar δ^{15}N patterns along successional gradients at plant community and species levels. *Geophysical Research Letters* **34**, 2007GL030722 (2007).

475. Wang, Q., Watanabe, M. & Ouyang, Z. Simulation of water and carbon fluxes using BIOME-BGC model over crops in China. *Agricultural and Forest Meteorology* **131**, 209–224 (2005).

476. Wang, W., Guo, J. & Oikawa, T. Contribution of root to soil respiration and carbon balance in disturbed and undisturbed grassland communities, northeast China. *J Biosci* **32**, 375–384 (2007).

477. Wang, Z. *et al.* Impacts of mixed-grazing on root biomass and belowground net primary production in a temperate desert steppe. *R. Soc. open sci.* **6**, 180890 (2019).

478. Warembourg, F. R. & Paul, E. A. Seasonal transfers of assimilated 14C in grassland: Plant production and turnover, soil and plant respiration. *Soil Biology and Biochemistry* **9**, 295–301 (1977).

479. Waring, R. *et al.* Why is the productivity of Douglas-fir higher in New Zealand than in its native range in the Pacific Northwest, USA? *Forest Ecology and Management* **255**, 4040–4046 (2008).

480. Welker, J. M., Fahnestock, J. T., Henry, G. H. R., O'Dea, K. W. & Chimner, R. A. CO_2 exchange in three Canadian High Arctic ecosystems: response to long-term experimental warming. *Global Change Biology* **10**, 1981–1995 (2004).

481. White, S., Bork, E., Karst, J. & Cahill, J. Similarity between grassland vegetation and seed bank shifts with altered precipitation and clipping, but not warming. *Community Ecology* **13**, 129–136 (2012).

482. White, T. A., Johnson, I. R. & Snow, V. O. Comparison of outputs of a biophysical simulation model for pasture growth and composition with measured data under dryland and irrigated conditions in New Zealand. *Grass and Forage Science* **63**, 339–349 (2008).

483. Whittinghill, K. A. & Hobbie, S. E. Effects of Landscape Age on Soil Organic Matter Processing in Northern Alaska. *Soil Sci. Soc. Am. j.* **75**, 907–917 (2011).

484. Wieder, R. K. Past, Present, and Future Peatland Carbon Balance: An Empirical Model Based on 210 Pb-Dated Cores. *Ecological Applications* **11**, 327 (2001).

485. Wielgolaski, F. E. *Fennoscandian Tundra Ecosystems: Part 1 Plants and Microorganisms*. (Springer Berlin Heidelberg, Berlin, Heidelberg, 1975).

486. Wielgolaski, F. E. Primary productivity of alpine meadow communities. in *Fennoscandian Tundra Ecosystems. Part 1: Plants and Microorganisms* (ed. Wielgolaski, F. E.) vol. 16 366 (Springer-Verlag, 1975).

487. Wilcox, K. R., Von Fischer, J. C., Muscha, J. M., Petersen, M. K. & Knapp, A. K. Contrasting above- and belowground sensitivity of three Great Plains grasslands to altered rainfall regimes. *Global Change Biology* **21**, 335–344 (2015).

488. Williams, M. *et al.* Predicting Gross Primary Productivity in Terrestrial Ecosystems. *Ecological Applications* **7**, 882–894 (1997).

489. Wu, Y. *et al.* Comprehensive assessments of root biomass and production in a Kobresia humilis meadow on the Qinghai-Tibetan Plateau. *Plant Soil* **338**, 497–510 (2011).

490. Xiao, X., Peng, Y., Cui, X. & Ojima, D. S. Observation and Modeling of Plant Biomass of Meadow Steppe in Tumugi, Xingan League, Inner Mongolia, China. *Vegetatio* **127**, 191–201 (1996).

491. Xu, X. *et al.* Interannual variability in responses of belowground net primary productivity (NPP) and NPP partitioning to long-term warming and clipping in a tallgrass prairie. *Global Change Biology* **18**, 1648–1656 (2012).

492. Yamamoto, S., Murayama, S., Saigusa, N. & Kondo, H. Seasonal and inter-annual variation of CO_2 flux between a temperate forest and the atmosphere in Japan. *Tellus B: Chemical and Physical Meteorology* **51**, 402 (1999).

493. Yan, L., Chen, S., Huang, J. & Lin, G. Differential responses of auto- and heterotrophic soil respiration to water and nitrogen addition in a semiarid temperate steppe. *Global Change Biology* **16**, 2345–2357 (2010).

494. Yan, L., Chen, S., Huang, J. & Lin, G. Water regulated effects of photosynthetic substrate supply on soil respiration in a semiarid steppe: water regulated effects of substrate supply on soil respiration. *Global Change Biology* **17**, 1990–2001 (2011).

495. Yang, Y. *et al.* Warming of alpine tundra enhances belowground production and shifts community towards resource acquisition traits. *Ecosphere* **11**, e03270 (2020).

496. Yang, Y.-S., Chen, G.-S., Guo, J.-F., Xie, J.-S. & Wang, X.-G. Soil respiration and carbon balance in a subtropical native forest and two managed plantations. *Plant Ecol* **193**, 71–84 (2007).

497. Yang, Y.-S., Chen, G.-S., Lin, P., Xie, J.-S. & Guo, J.-F. Fine root distribution, seasonal pattern and production in four plantations compared with a natural forest in Subtropical China. *Ann. For. Sci.* **61**, 617–627 (2004).

498. Yashiro, Y. *et al.* Biometric-based estimation of net ecosystem production in a mature Japanese

cedar (Cryptomeria japonica) plantation beneath a flux tower. *J Plant Res* **123**, 463–472 (2010).

499. Yasuda, Y. Measurement of CO2 flux above a tropical rain forest at Pasoh in Peninsular Malaysia. *Agricultural and Forest Meteorology* **114**, 235–244 (2003).

500. Yazaki, Y., Mariko, S. & Koizumi, H. Carbon dynamics and budget in a *Miscanthus sinensis* grassland in Japan. *Ecological Research* **19**, 511–520 (2004).

501. Yu, J. *et al.* Estimating Net Primary Productivity and Nutrient Stock in Plant in Freshwater Marsh, Northeastern China. *CLEAN Soil Air Water* **38**, 1080–1086 (2010).

502. Yuste, J. C. *et al.* Contrasting net primary productivity and carbon distribution between neighboring stands of Quercus robur and Pinus sylvestris. *Tree Physiology* **25**, 701–712 (2005).

503. Zanotelli, D., Montagnani, L., Manca, G. & Tagliavini, M. Net primary productivity, allocation pattern and carbon use efficiency in an apple orchard assessed by integrating eddy covariance, biometric and continuous soil chamber measurements. *Biogeosciences* **10**, 3089–3108 (2013).

504. Zeglin, L. H., Stursova, M., Sinsabaugh, R. L. & Collins, S. L. Microbial responses to nitrogen addition in three contrasting grassland ecosystems. *Oecologia* **154**, 349–359 (2007).

505. Zeng, C., Wu, J. & Zhang, X. Effects of Grazing on Above- vs. Below-Ground Biomass Allocation of Alpine Grasslands on the Northern Tibetan Plateau. *PLoS ONE* **10**, e0135173 (2015).

506. Zenone, T. *et al.* CO2 fluxes of transitional bioenergy crops: effect of land conversion during the first year of cultivation: CO_2 fluxes of transitional bioenergy crops. *GCB Bioenergy* **3**, 401–412 (2011).

507. Zenone, T., Gelfand, I., Chen, J., Hamilton, S. K. & Robertson, G. P. From set-aside grassland to annual and perennial cellulosic biofuel crops: Effects of land use change on carbon balance. *Agricultural and Forest Meteorology* **182–183**, 1–12 (2013).

508. Zha, T. *et al.* Carbon sequestration in boreal jack pine stands following harvesting. *Global Change Biology* **15**, 1475–1487 (2009).

509. Zhang, P. *et al.* Characterization of CO2 flux in three Kobresia meadows differing in dominant

species. *Journal of Plant Ecology* **2**, 187–196 (2009).

510. Zheng, X. *et al.* Quantifying net ecosystem carbon dioxide exchange of a short-plant cropland with intermittent chamber measurements. *Global Biogeochemical Cycles* **22**, 2007GB003104 (2008).

511. Zimmermann, M., Meir, P., Bird, M. I., Malhi, Y. & Ccahuana, A. J. Q. Temporal variation and climate dependence of soil respiration and its components along a 3000 m altitudinal tropical forest gradient. *Global Biogeochemical Cycles* **24**, 2010GB003787 (2010).

512. Author(s): Jack A. Morgan, Arvin R. Mosier, Daniel G. Milchunas, Daniel R. LeCain, Jim A. Nelson and William J. Parton Reviewed work(s): *Ecological Applications* **14**, 208–219 (2004).
